# *Erica spiculifolia* Salisb. (Balkan Heath): A Focus on Metabolic Profiling and Antioxidant and Enzyme Inhibitory Properties

**DOI:** 10.3390/plants14111648

**Published:** 2025-05-28

**Authors:** Reneta Gevrenova, Anna Szakiel, Cezary Pączkowski, Gokhan Zengin, Inci Kurt-Celep, Alexandra Stefanova, Dimitrina Zheleva-Dimitrova

**Affiliations:** 1 Department of Pharmacognosy, Faculty of Pharmacy, Medical University-Sofia, 1000 Sofia, Bulgaria; dzheleva@pharmfac.mu-sofia.bg; 2 Department of Plant Biochemistry, Institute of Biochemistry, Faculty of Biology, University of Warsaw, 1 Miecznikowa Street, 02-096 Warsaw, Poland; a.szakiel@uw.edu.pl (A.S.); c.paczkowski@uw.edu.pl (C.P.); 3 Physiology and Biochemistry Research Laboratory, Department of Biology, Science Faculty, Selcuk University, Konya 42130, Turkey; gokhanzengin@selcuk.edu.tr; 4 Department of Biotechnology, Faculty of Pharmacy, İstanbul Okan University, Tuzla, İstanbul 34940, Turkey; inci.celep@okan.edu.tr; 5 Department of Pharmacology, Pharmacotherapy and Toxicology, Faculty of Pharmacy, Medical University-Sofia, 1000 Sofia, Bulgaria; astefanova22@gmail.com

**Keywords:** *Erica spiculifolia*, proantocyanidins, LC-MS, GC-MS, antioxidants, enzyme inhibitory potential

## Abstract

*Erica spiculifolia* Salisb. (formerly *Bruckenthalia spiculifolia* Benth.) (Balkan heath) is renowned for its traditional usage as a diuretic, anti-inflammatory and antioxidant agent. For the first time, acylquinic acids, flavonoids and numerous proanthocyanidin oligomers were annotated/dereplicated by liquid chromatography–high-resolution mass spectrometry in methanol–aqueous extracts from *E*. *spiculifolia* aerial parts harvested at the early and full flowering stage. Chlorogenic acid and proanthocyanidin tetra- and trimer A, B-type together with quercitrin and (+) catechin were the predominant compounds in the semi-quantitative analysis. Neutral triterpenoids, triterpenoid acids and phytosterols were determined in apolar extracts by gas chromatography–mass spectrometry. Triterpenoid acids accounted for 80% of the total triterpenoid content, dominated by ursolic and oleanolic acid, reaching up to 32.2 and 6.1 mg/g dw, respectively. Ursa/olean-2,12-dien-28-oic acids and 3-keto-derivatives together with α-amyrin acetate as a chemotaxonomic marker, α-amyrenone, α- and β-amyrin were evaluated. Total phenolic and flavonoid contents were 83.85 ± 0.89 mg gallic acid equivalents/g and 78.91 ± 0.41 mg rutin equivalents/g, respectively. The extract actively scavenged DPPH and ABTS radicals (540.01 and 639.11 mg Trolox equivalents (TE)/g), possessed high potential to reduce copper and iron ions (660.32 and 869.22 mg TE/g, respectively), and demonstrated high metal chelating capacity (15.57 Ethylenediaminetetraacetic acid equivalents/g). It exhibited prominent anti-lipase (18.32 mg orlistat equivalents/g) and anti-tyrosinase (71.90 mg kojic acid equivalents/g) activity. The extract inhibited α-glucoside (1.35 mmol acarbose equivalents/g) and acetylcholinesterase (2.56 mg galanthamin equivalents/g), and had moderate effects on α-amylase, elastase, collagenase and hyaluronidase. Balkan heath could be recommended for raw material production with antioxidant and enzyme inhibitory properties.

## 1. Introduction

The *Erica* L. genus, belonging to the Ericaceae family, consists of evergreen shrubs known for adapting to nutrient-poor soils [1]. *Erica spiculifolia* Salisb. (formerly *Bruckenthalia spiculifolia* (Salisb.) Reichb.) is native to Eastern Europe and Western Asia, from the northern Carpathians through the Balkans into northeastern Turkey [2,3]. The species is distributed on acid soils in the mountain shrublands from 1400 to 2500 m. *E. spiculifolia* is commonly referred to as Balkan heath. It is an evergreen shrub with small flowers (corolla 2.5–3 mm long), clustered in terminal inflorescences.

Thorough assessments of the secondary metabolites and pharmacological activity of *E. spiculifolia* are almost nonexistent. To the best of our knowledge, there are little data on the phytochemical composition of the species. Pavlović et al. (2009) have found that *E. spiculifolia* aboveground parts contain 4.67 ± 0.08% total polyphenols, 3.71 ± 0.05% flavonoids, 0.86 ± 0.06% non-tannins and 3.80 ± 0.06% tannins, while hydroquinone derivatives account for 0.5% [4]. Balkan heath roots accumulate 194.41 ± 5.74 mg catechin equivalents (CE)/g total polyphenols, 4.23 ± 12.29 mg CE/g non-tannins, 150.17 ± 7.52 mg CE/g total tannins and 3.5 ± 0.06 mg rutin equivalents/g total flavonoids [5]. Quercitrin, isoquercitrin and quercetin dominate the phenolic compounds, being present at 70.91, 32.50 and 3.77 mg/g dry extract, respectively [6]. Overall, 100 compounds have been reported in the essential oil which represents 0.034% of the dry mass from the aerial parts of *E. spiculifolia* with Bulgarian provenance [7]. Oxygenated monoterpenes reach high levels (45.3%). α-Terpineol (7.5%), *endo*-borneol (7.2%), pinocarveol (5.9%) and thymol (3.7%) dominate the essential oil. Oxygenated sesquiterpenes (20.3%) are the most abundant within the sesquiterpenoids—caryophyllene oxide, spathulenol and α-cadinol reach up to 5.0, 2.9 and 2.3%, respectively, along with the sesquiterpene hydrocarbon caryophyllene (4.2%).

As far as we know, the available literature data on the traditional usage of the Balkan heath are scarce. *E. spiculifolia* has been used along with *E. arborea*, *E. manipuloflora*, *E. boquetii* and *E. cicula-libanotica* in the ethnopharmacological approach as diuretic, antiseptic, wound healing and anti-inflammatory agents [1,8]. In Europe, the main use of the *Erica* species is to treat urinary tract infections, kidney stones and respiratory diseases [9]. The phytochemistry, ethnomedicinal use and pharmacological properties of the *Erica* genus are the subject of a review by Adu-Amankwaah et al. (2025) which delineates over 60 bioactive compounds found in the *Erica* species including phenolic acids, flavonoids and their acetyl/hydroxybenzoyl/hydroxycinnamoyl esters [1]. The authors highlighted the antioxidant and enzyme inhibitory potential of *E. arborea* extracts towards cholinesterases, tyrosinase, α-glucosidase and α-amylase, suggesting that they may be used to manage postprandial hyperglycemia. Indeed, Balkan heath ethanol–aqueous extracts exhibited dose-dependent activity in a β-carotene bleaching assay and against lipid peroxidation, reaching 96% inhibition at 125 μg/mL [4,6]. Significant advances have been made in exploring the *E. spiculifolia*-mediated effects on inflammation. Aerial part and root extracts exerted anti-inflammatory activity in an in vitro model of carrageenan-induced paw oedema comparable with indomethacin [5,10]. Indeed, both extracts inhibited the denaturation of bovine serum album by 50 and 87%; the activity was similar to that of diclofenac. In an in vivo model of surfactant-irritated skin, a gel containing 2% dry leaf extract from *E. spiculifolia* significantly reduced the degree of skin irritation [6]. The arachidonic acid metabolite pathway and the inhibition of nitric oxide production may be implicated in protection from inflammation [5,10,11]. The anti-inflammatory, antimicrobial and antioxidant activity of *E. spiculifolia* extracts sustain the traditional claims relating to their use for the mitigation of inflammatory issues and skin disorders.

In addition to evoking an anti-inflammatory response, some *Erica* species display cytotoxic activity towards tumor cell lines [1]. Despite the accumulating evidence that *E. manipuliflora, E. andevalensis* and *E. glabella* Thunb. extracts show anti-proliferative activity against hepatoblastoma, breast and renal adenocarcinoma, and melanoma cell lines, the mechanisms have not been thoroughly investigated. Promising cytotoxic activity was evidenced for ursolic acid, commonly found in the Ericaceae family [1]. Ericaceae provides a rich source of triterpenoids and proanthocyanidins (PACs), and the unique nature of the health-promoting benefits of *Vaccinium*-derived PACs has been associated with their antimicrobial, anti-inflammatory, antioxidant and neuroprotective effects [12]. In this regard, only the proanthocynidin trimer has been recently reported in *E. glabella* [13].

It is noteworthy that the aforementioned studies emphasize the antioxidant and anti-inflammatory effects of *E. spiculifolia* along with certain *Erica* species, while there is no comprehensive metabolite profiling of Balkan heath by means of both liquid chromatography– and gas chromatography–high-resolution mass spectrometry. Our objective was to examine whether the Balkan heath aerial parts contain PACs together with acylquinic acids, flavonoids, triterpenoids and phytosterols, and whether the methanol–aqueous extracts exhibit prominent activity via different mechanisms in antioxidant assays. Owing to the progress of technology platforms in hyphenated analytical methods, an exponential increase in analyses of PACs in *Vaccinium*, *Rubus* and *Filipendula* species has been observed [14,15,16,17]. Essential aspects in the high-level structural analysis of PACs in LC-MS have been recently emphasized in the valuable studies by Tang et al. (2022) and Symma and Henseld (2022) [12,18]. Collectively, the cited studies generate further interest in the Balkan heath and prompted us to undertake in-depth profiling of secondary metabolites in *E. spiculifolia* aerial parts by means of ultra-high-performance liquid chromatography coupled with hybrid quadrupole–Orbitrap high-resolution mass spectrometry and gas chromatography–mass spectrometry. This study was integrated with an assessment of antioxidant activity and enzyme inhibitory potential towards key targets in therapies including α-amylase, α-glucosidase, tyrosinase, cholinesterases, lipase, elastase, collagenase and hyaluronidase.

## 2. Results and Discussion

### 2.1. LC-HRMS Profiling of Secondary Metabolites in Erica spiculifolia Methanol–Aqueous Extracts (ES1 and ES2)

Balkan heath extracts were found to contain a highly complex mixture of acylquinic acids, proanthocyanidins and flavonoids; more than 100 individual phenolics were tentatively identified. Corresponding molecular formulas, their MS/MS fragmentation patterns, retention times and mass measurement errors are depicted in Table S1. None of the compounds except for quercitrin, isoquercitrin and quercetin have ever been reported in the Balkan heath. For comparative purposes, a semi-quantitative analysis of two plant samples ES1 and ES2 was performed. The results from the semi-quantitative analysis of the individual polyphenols are presented in Table S2.

Acylquinic acids (ACAs)

Overall, 10 monoAQAs and 1 diAQAs along with rosmarinic acid were identified or annotated in *E. spiculifolia* extracts. The fragmentation pattern of each subclass of AQAs is reported elsewhere [19,20]. Accordingly, **2**/**6**, **7**/**10** and **9** were ascribed to 5-caffeoyl, 5-*p*-coumaroyl and 5-feruloylquinic acid, respectively, as suggested by the base peak at *m/z* 191.055 [quinic acid-H]^−^ (Table 1 and Table S1). Compounds **1** and **4** were identified as neochlorogenic and 3-feruloylquinic acid, while **3** and **8** were assigned as 4-substituted derivatives on the base of the “dehydrated” ion of quinic acid at *m*/*z* 173.044. Neochlorogenic (**1**), chlorogenic (**2**), 3, 5-dicaffeoylquinic (**11**) and rosmarinic (**12**) acid were unambiguously identified by comparison with reference standards (Figures S2–S5). Within this group, the profile was dominated by chlorogenic acid (**2**) (43.29% in ES1 and 55.00% in ES2), followed by its isomer 4-caffeoylquinic acid (**3**) (20.72% in ES1 and 17.72% in ES2), and 5-*p*-coumaroylquinic acid (**7**) (9.60% in ES1 and 8.60% in ES2). Overall, acylquinic acids accounted for 9.74% (ES1) and 6.10% (ES2) of the assayed compounds.

Proanthocyanidins (PACs)

A variety of proanthocyanidins (PACs) including 8 dimers, 8 trimers, 10 tetramers, 11 pentamers, 1 hexamer and 1 heptamer were annotated in the assayed extracts (Table 1 and Table S1, Figure S1).

Depending on the linkage between the monomeric units, 16 of them could be identified as B-type characterized by a carbon–carbon interflavan bond [18]. An additional carbon–oxygen linkage and A-type PACs were established in 4 compounds, while 17 could be identified as hybrid A, B-type. The main criteria in the peak annotation of the PACs according to the ESI-MS/MS spectra were the neutral mass losses in the specific fragmentation reactions, i.e., heterocyclic ring fission (HRF, −126 Da) and Retro–Diels–Alder fission (RDA, −152 Da) along with the loss of an additional molecule of water (152 + 18 Da) and quinine methane or interflavan fission (QM, −288 or −290 Da), as reported in the literature [12,15,18]. Afterwards, the fragmentation patterns yielded the diagnostic fragment ion at *m*/*z* 289.072 consistent with the deprotonated molecule of (epi)catechin moiety (C). The MS/MS fragmentation pathway of (epi)catechin was delineated by the fragment ions at *m*/*z* 245.082 [C-H-CO_2_]^−^, 229.050 [C-H-H_2_O-C_2_H_2_O]^−^, 203.070 [C-H-CO_2_-C_2_H_2_O]^−^, 179.034 [C-H-C_6_H_6_O_2_]^−^ and 161.023 [C-H-C_6_H_6_O_2_-H_2_O]^−^ together with RDA ions at *m*/*z* 137.02023 (^1,3^A^−^), 125.023 (^1,4^A^−^) and 109.0279 (^1,3^A^−^/CO) (Table S1). Owing to the structural complexity, regio- and stereo-isomers occurred within the PAC class [18]. Most of the annotated PACs in Balkan heath extracts occurred in several isomeric forms along the chromatogram (Figure S1). Herein, the number of (epi)catechin units and the number of A-type and B-type linkages were evidenced using high-energy collision-induced dissociation in negative ion mode Orbitrap mass spectrometry and Table S1). (-) ESI-MS/MS allowed for rapid annotation of oligomeric PACs on the base of the deprotonated molecules/double-charged ions together with QM/RDA and HRF pathways. A- and B-type bonding between proanthocyanidin units was witnessed by the inspecting QM fragment ions along with the RDA fission.

**Figure 1:**
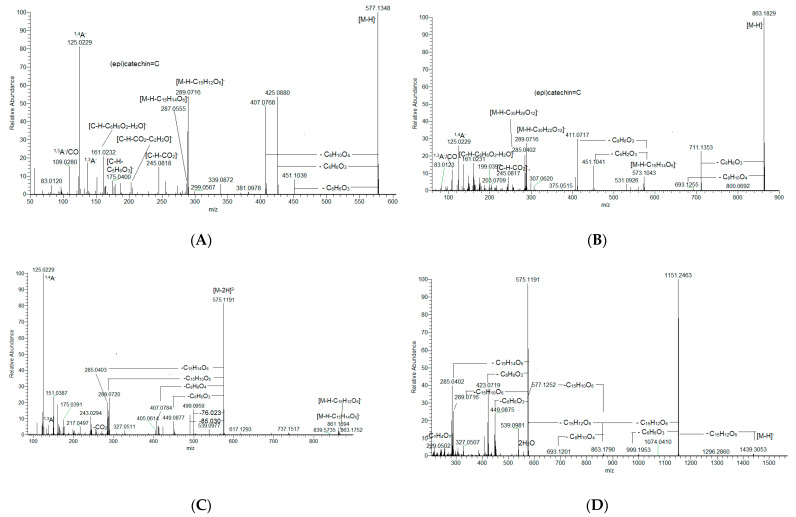
MS/MS spectra of proanthocyanidin dimer (**22**) (**A**), proanthocyanidin trimer (**26**) (**B**), proanthocyanidin tetramer (**38**) (**C**) and proanthocyanidin pentamer (**47**) (**D**).

Six isobars **14**, **15**, **18**, **22**, **30** and **44** shared the same [M-H]^−^ at *m*/*z* 577.135 consistent with C_30_H_25_O_12_ (Table 1 and Table S1, Figure 1A). The prominent QM ions at *m*/*z* 289.072 and 287.056 suggested the losses of 288 Da (C_15_H_12_O_6_) and 290 Da (C_15_H_14_O_6_), as has been observed in B-type proanthocyanidin dimers [15,18]. This assumption was in line with the presence of an HRF ion at *m*/*z* 451.103 [M-H-C_6_H_6_O_3_]^−^ and RDA ions at *m*/*z* 425.088 [M-H-C_8_H_8_O_3_]^−^and 407.077 [M-H-(C_8_H_8_O_3_ + H_2_O]^−^. The isomeric pair **37** and **49** with [M-H]^−^ at *m*/*z* 575.135 (consistent with C_30_H_23_O_12_) afforded indicative QM ions at *m*/*z* 289.072 (16%) and 285.040 (27%), pointing out the presence of C-O linkage in the dimers (Figure S6). Accordingly, the aforementioned compounds were ascribed as A-type dimers.

MS/MS spectra of a series of trimers (**17**, **21, 27**, **33** and **43**) with [M-H]^−^ at *m*/*z* 865.199 consistent with C_45_H_37_O_18_ were acquired (Table 1 and Table S1). Their structures were deduced from the transitions *m*/*z* 865.199 → 577.134 → 289.072 resulting from the consequent loss of 288 Da (QM fragmentation). A pair of RDA ions for each B-type linkage was registered at *m*/*z* 713.151 [M-H-C_8_H_8_O_3_]^−^/695.143 [M-H-(C_8_H_8_O_3_ + H_2_O]^−^ and 425.090/407.078 (**17**) (Table S1, Figure S7). Consequently, the aforementioned trimers were ascribed to B-type PACs.

Exemplified by **25**, **26** and **48** with [M-H]^−^at *m*/*z* 863.183 (C_45_H_37_O_18_), hybrid A, B-type trimers were evidenced (Table 1 and Table S1, Figure 1B). RDA ions at *m*/*z* 711.136 and 693.126 pointed out the B-type linkage. QM ions at *m*/*z* 289.072 and 285.040 indicated A-type linkage corroborated by HRF ions at *m*/*z* 451.103 and 411.072, as has been previously observed for mixed-type trimers [15].

Compounds **13**, **20** and **29** afforded [M-H]^−^ at *m*/*z* 1151.247 (C_60_H_47_O_24_), indicating tetramers. Their assignment was based on the QM ions at *m*/*z* 863.183 (ΔM 288, 18.4%), 573.104 (ΔM 290, 4%) and 285.040 (ΔM 288, 15.4%) (Table S1, Figure S8). Two pairs of RDA ions at *m*/*z* 999.200/981.196 and 711.125/693.125 suggested the presence of two B-type linkages, while the third one was ascribed to A-type (see dimers A-type). It is noteworthy that several tetra- and pentamers were detected through their double-charged ions [M-2H]^2−^, which were confirmed by the 0.5 Da mass differences between the peaks in monoisotopic patterns. The selected resolution of 70,000 allowed for the determination of the charge state with high accuracy. Compounds **38**, **41** and **46** yielded double-charged ions at *m*/*z* 575.119 [M-2H]^2−^ (C_60_H_46_O_24_). RDA ions at *m*/*z* 499.096 [M-2H-76.023]^2−^ and 490.089 [M-2H-85.030]^2−^ along with an HRF ion at *m*/*z* 449.0877 [M-2H-126.031]^2−^ indicated a B-type linkage, while A-type was evidenced by the QM ions at *m*/*z* 289.073 and 285.040 (Table S1, Figure 1C and Figure S9A). Accordingly, the aforementioned compounds were ascribed to hybrid A, B-type.

On the other hand, the double-charged ion at *m*/*z* 576.127 [M-2H]^2−^ (consistent with C_60_H_48_O_24_) (**23** and **35**) underwent losses of 76.023 and 85.028 Da at *m*/*z* 500.104 and 491.099, respectively, according to the B-type RDA fragmentation (Figures S9B and S10). Moreover, the precursor ion yielded QM ions at *m*/*z* 289.072 and 287.056. Thus, **23** and **35** were assigned as B-type tetramers. In the same way, a B-type tetramer was proposed for **31** with [M-H]^−^at *m*/*z* 1153.261 consistent with C_60_H_49_O_24_, as has been previously observed for B-type tetramers [15,17].

The pentamer **47** (consistent with C_75_H_60_O_30_) was deduced from the QM transitions *m*/*z* 1439.305 → 1151.246 → 863.179 → 575.119 → 289.072/285.040, corroborated by the HRF ion at 449.088 and RDA ions at *m*/*z* 423.072 and 407.077 (Table S1, Figure 1D). Thus, B-type was tentatively ascribed to three linkages, while the last one was unambiguously assigned as A-type.

Six compounds **16**, **19**, **24**, **36**, **40** and **42** share the same doubly charged ion at *m*/*z* 719.151 [M-2H]^2−^ (Table 1 and Table S1, Figure S11). A B-type linkage was discernable by the HRF ion at *m*/*z* 656.136 [M-2H-63]^2−^, RDA ions at *m*/*z* 643.128 [M-2H-76]^2−^ and 634.122 [M-2H-85]^2−^ and QM at *m*/*z* 575.120 [M-2H-144]^2−^ (Table S1). QM ions at *m*/*z* 285.040 and 289.072 supported by the HRF ion at *m*/*z* 411.072 suggested the terminal A-type linkage. Accordingly, the aforementioned pentamers were assigned as hybrid A, B-type. In the same way, B-type linkages were annotated in **32** and **34** (C_75_H_62_O_30_) at *m*/*z* 720.159 [M-2H]^2−^, while **45** and **51** (C_75_H_58_O_30_) at *m*/*z* 718.144 [M-2H]^2−^were assigned as A-type PACs (Figures S12 and S13). A higher degree of polymerization was established in compound **28** at *m*/*z* 863.182 [M-2H]^2−^ (hexamer, C_90_H_72_O_36_) and **39** at *m*/*z* 1008.724 [M-2H]^2−^ (heptamer, C_105_H_87_O_42_), both ascribed to hybrid A, B-type (Table S1, Figures S14 and S15).

Herein, all PACs are reported for the first time in the genus *Erica*. Among these PACs, being present at 14.86% (ES1) and 9.76% (ES2) of the studied compounds, proanthocyanidin tetramer and pentamer A, B-type (**46** and **47**) (23.10% in ES1 and 43.69% in ES2), together with proanthocyanidin trimer A, B-type (**26**) (10.40% in ES1 and 10.81% in ES2), proanthocyanidin dimer A-type (**37**) (9.60% in ES1) and proanthocyanidin dimer B-type (**22**) (5.27% in ES2), appeared to be dominant for the studied extracts.

Flavonoids

The approach for flavonoid annotation has been delineated elsewhere [19,21]. Concerning **54**, a typical di*C*-hexosyl flavonoid pattern was generated including the fragment ions at *m*/*z* 415.104 [M-H-60-120]^−^, 385.093 [M-H-120-90]^−^ and 355.082 [M-H-120-120]^−^. The aglycone naringenin was evidenced by the prominent fragment ions at *m*/*z* 271.062 corroborated by the RDA ions at *m*/*z* 151.002 (^1,3^A^−^), 119.049 (^1,3^B^−^) and 107.012 (^0,4^A^−^) (Table S1). Accordingly, **54** was assigned as naringenin 6, 8-diC-hexoside. A *C*, *O*-dihexoside (**65**) was discernable by the concomitant losses of (162 + 90 Da) at *m*/*z* 341.068 and (162 + 120 Da) at *m*/*z* 311.056. In addition, fragment ions at *m*/*z* 269.046 and 117.033 (^1,3^B^−^) pointed out the aglycone apigenin. Thus, apigenin 6-*C*-8-*O*-diglucoside (saponarin) was unambiguously identified by comparison with a standard reference. A variety of flavonol- hexosides and pentosides were identified/annotated including quercetin (**74**, **77**, **81**, **82**, **84**), myricetin (**67**, **68**, **70**, **71**), gossypetin (**56**, **60**–**62, 66**) and isorhamnetin (**83**) glycosides. Compounds **75, 78** and **90** were strongly related with the same fragmentation pathway yielding [M-H-176]^−^, which is consistent with the hexuronide of quercetin, luteolin and apigenin, respectively. Hydroxycinnamoyl moieties were discernable in quercetin–glycosides **93**, **103** and **105** by the characteristic transitions 625.120 → 463.088 → 301.035/300.027 (**93**) and 593.130 → 301.034/300.027 (**103** and **105**). Thus, the aforementioned compounds were ascribed to caffeoylhexosyl- and cinnamoylhexosyl-quercetin. Flavonoid diglycosides were delineated by the rutinosides (**58, 72, 76** and **85**), quercetin–pendosylhexoside (**73**) and dihexoside (**64**).

(+) Catechin (**53**), rutin (**72**), isoquercetin (**74**), hyperoside (**77**), kaempferol 3-*O*-rutinoside (**83**), isorhamnetin 3-*O*-rutinoside (**85**), quercitrin (**86**), isorhamnetin 3-*O*-glucoside (**87**), apigenin 7-*O*-glucoside (**89**), luteolin (**98**), quercetin (**99**), apigenin (**100**), kaempferol (**101**), hispidulin (**102**) and chrysoeriol (**104**) were unambiguously identified by comparison with reference standards. The (-) ESI-MS/MS spectra of the identified flavonoids are depicted in Figures S16–S33. Overall, quercitrin (**86**) (14.48% in ES1 and 25.21% in ES2), epicatechin (**57**) (13.91%in ES1 and 12.15% in ES2) and quercetin *O*-pentoside (**84**) (9.91% in ES1 and 10.27 in ES2) were found to be the predominant flavonoids, followed by catechin (**53**) (8.31% in ES1 and 5.56% in ES2) and isoquercitrin (**74**) (7.59% in ES1 and 7.89% in ES2).

Owing to the fact that the ES2 methanol–aqueous extract contained a higher level of flavonoids (84.18%) than ES1 (75.53%), and considering the relative content of chlorogenic acid (**2**) and proanthocyanidin tetra- and pentamers (**46** and **47**), it was selected for further assessment of the total bioactive compounds, as well as antioxidant and enzyme inhibitory properties.

### 2.2. GC-MS Analysis

GC-MS analysis of triterpenoids and steroids occurring in *E. spiculifolia* revealed the presence of compounds typical of these groups. In all analyzed samples (including mixed aerial parts, flowers and leaves), a group of oleanane- and ursane-type triterpenoid acids were identified, ursolic and oleanolic acids, along with their derivatives featuring an additional double bond—namely ursa- and olean-2,12-dien-28-oic acids—and keto-derivatives such as 3-oxo-ursolic and 3-oxo-oleanolic acids (Table S3). The primary acids (oleanolic and ursolic) were identified by comparing their mass spectra with data from the Wiley-NIST library and by matching their retention times and corresponding mass spectra with those of authentic standards. The derivatives with an additional double bond, as well as keto-derivatives, were identified based on comparisons with library spectra and data from previous reports. A compound referred to as “unsaturated ursolic acid” (likely ursa-2,12-dien-28-oic acid) was previously identified in the stems and leaves of *Calluna vulgaris*, a plant belonging to the Ericaceae family [22]. The presence of the two 3-oxo acids was also reported in another study on *Calluna vulgaris* flower and leaflet cuticular waxes, where their identification was further confirmed by comparing retention times and mass spectra with those of oxidized authentic standards of oleanolic and ursolic acid methyl esters [23]. Ursolic and oleanolic acid derivatives have also been previously reported in other plant species known for their rich content of triterpenoid acids [24]. Additionally, in the fractions of methylated triterpenoid acids from all analyzed samples of *E. spiculifolia*, another unidentified triterpenoid acid was detected in significant amounts, and it was tentatively identified on the basis of the literature as micromeric acid (3β –hydroxyursa-12,20(30)-dien-28-oic acid) methyl ester (Figure S34), previously reported in another Erica species, *E. erigena* [25]. The neutral triterpenoid fraction of *E. spiculifolia* consisted of α-amyrin (urs-12-en-3β-ol) and β-amyrin (olean-12-en-3β-ol), α-amyrenone (urs-12-en-3-one), α-amyrin acetate, germanicol (olean-18-en-3β-ol), taraxasterol (taraxast-20(30)-en-3β-ol), erythrodiol (olean-12-ene-2,28-diol) and uvaol (urs-12-ene-3,28-diol), as well as oleanolic aldehyde (3β-hydroxy-olean-12-en-28-al) and ursolic aldehyde (3β-hydroxy-urs-12-en-28-al) (Table S3). These compounds were identified by comparing their mass spectra with the Wiley-NIST library, and in the case of both amyrins and uvaol, identification was additionally confirmed by matching their retention times and corresponding mass spectra with those of authentic standards. Overall, the triterpenoid profile of *E. spiculifolia* closely resembles that of other members of the Ericaceae family [26], including the presence of α-amyrin acetate, which is considered a biomarker for *Erica* species [27]. However, the absence of lupane- and taraxerane-type triterpenoids suggests a certain degree of taxonomic distinctiveness for this plant.

The steroid fraction was composed of two typical phytosterols, sitosterol (stigmast-5-en-3β-ol), accompanied by its saturated form, sitostanol, as well as campesterol (24*R*-ergost-5-en-3β-ol), one steroid ketone tremulone (stigmasta-3,5-dien-7-one) and 24-methylenecycloartanol (9 β-24-methylene-9,19-cyclolanostane). The identification of campesterol and sitosterol was confirmed by comparison with authentic standards, while the remaining steroids were identified based on their mass spectra using Wiley-NIST library data.

The content of triterpenoids and steroids determined in *E. spiculifolia* samples collected in July and August is presented in Table 2. The results indicate that the aerial parts of the plant (a mixture of flowers, leaves and stems), as well as separated flowers and leaves, are rich sources of these compounds, reaching up to nearly 4.3% of the dry weight of the aerial parts and 5.3% of the dry weight of the separated leaves. Separated flowers contained 41% fewer triterpenoids than the leaves.

Similar to other representatives of the *Erica* species [26,27], *E. spiculifolia* was found to be particularly rich in triterpenoid acids. These compounds accounted for approximately 80% of the total triterpenoid content in flowers and mixed aerial parts, and up to 85% in leaves. The predominant compound was ursolic acid, comprising up to 65% of the total acids in flowers and aerial parts, and 71% in leaves, followed by oleanolic acid, which made up approximately 15% of the total acid content. Ursane-type triterpenoids generally predominated in *E. spiculifolia,* as evidenced by the high content of α-amyrin (about 43% of the neutral triterpenoid fraction) and the greater abundance of uvaol and ursolic aldehyde compared to the oleane-type compounds erythrodiol and oleanolic aldehyde. Among the steroids, sitosterol was the most abundant compound, constituting up to 75% of the steroid fraction.

### 2.3. Total Bioactive Compounds and Antioxidant and Enzyme Inhibitory Properties of E. spiculifolia Methanol–Aqueous Extract (ES2)

The total phenolic and flavonoid contents were assessed using spectrophotometric methods (Table 3). Our results reveal a higher total phenolic content (83.85 ± 0.89 mg GAE/g) and flavonoid content (78.91 ± 0.41 mg RE/g) in comparison with those previously reported by Pavlovic et al. (2009), where the aboveground parts contain 4.67 ± 0.08% polyphenols and 3.71 ± 0.05% flavonoids [4]. Recently, lower levels of total flavonoids (3.5 ± 0.06 mg RE/g) along with abundant total polyphenols (194.41 ± 5.74 mg CE/g) have been determined in *E. spiculifolia* roots [5]. Our data are in line with most reports on the genus *Erica*, where the flavonoids are the predominant group of bioactive compounds [10,28,29].

The antioxidant properties of plant extracts are closely associated with their capacity to defend against free radical assaults. Consequently, the assessment of a plant’s antioxidant properties can indicate its health-promoting benefits [30]. In this sense, we examined the antioxidant properties of *E. spiculifolia* methanol–aqueous extract by utilizing different chemical methods. The results are summarized in Table 3. The radical quenching capacity of the examined extract was assessed using DPPH and ABTS assays. The studied extract showed significant radical scavenging activity on DPPH (540.01 mg TE/g) and ABTS (639.11 mg TE/g). Reducing power serves as an indicator of antioxidant qualities and reflects the electron-donating capacity of antioxidants. To this end, CUPRAC and FRAP assays were performed; they include the transformation of Cu^2+^ to Cu^+^ and Fe^3+^ to Fe^2+^, respectively. The phosphomolybdenum assay, also considered a total antioxidant assay, involves the transformation of Mo (VI) to Mo (V) by antioxidants. The chelation of transition metals regulates hydroxyl radical formation in the Fenton reaction, hence serving as a significant antioxidant mechanism. As can be seen in Table 3, the studied extract had a strong reducing power in CUPRAC (869.22 mg TE/g) and FRAPS (660.32 mg TE/g) assays, as well as a metal chelating capacity of 15.57 mg EDTAE/g. The radical scavenging activity of *E. spiculifolia* has been previously reported [4,10]. The ethanol extract from aerial parts possessed antioxidant activity in the DPPH assay with IC_50_ 10.22 ± 0.74 µg/mL and showed pronounced activity against lipid peroxidation, reaching 96% inhibition in the β-carotene bleaching assay [4]. Furthermore, the *E. spiculifolia* extract exerted the strongest dose-dependent activity in the later assay among a few Ericaceae species [6]. Recently, Dragićević et al. (2024) reported that ethanolic root extract showed antioxidant activity with IC_50_ 2.59 ± 0.10 μg/mL and 9.10 ± 0.81 μg/mL in DPPH and β-carotene bleaching assays, respectively [5]. The antioxidant activity was ascribed to the high levels of polyphenols and tannins in the roots.

In the current study, the obtained antioxidant effects can be attributed to the presence of some biologically active compounds. For example, as can be seen from Table 1 and Table S2, the presence of chlorogenic acid, rosmarinic acid, quercetin, catechin and myricetin was observed and their antioxidant properties have been reported in earlier studies. Owing to the fact that chlorogenic and neochlorogenic acid dominated the acylquinic acids, it is worth noting that the caffeic acid conjugate possessed higher antiradical activity towards superoxide and hydroxyl radicals than caffeic acid [31]. For instance, chlorogenic acid has two hydroxyl groups in the aromatic ring which can act as hydrogen donors to free radicals [32]. Numerous investigations have demonstrated that proanthocyanidins, reported for the first time in Balkan heath, exhibit strong free radical scavenging activity owing to the presence of a large number of phenyl hydroxyl groups and their unique molecular stereochemistry [33]. In vivo studies showed that PACs mitigate oxidative stress injury, enhancing the antioxidant enzymes [34]. Indeed, proanthocyanidins with higher molecular weights are most effective when oxidation is initiated in the lipid domains, probably because the molecules insert into the lipid membrane and show better protection against oxidative stress in both hydrophobic and hydrophilic domains. In addition to evoking a nuclear factor erythroid 2-related factor (Nrf2)-dependent antioxidant response, PACs also display antiatherosclerotic and cardioprotective effects, which generates further interest in PACs as herbal drugs [33].

In addition, catechin and quercetin are considered effective antioxidants (dye to hydrogen donation) due to the ortho-dihydroxy groups in the B ring [35,36]. Based on the GC-MS results, the tested extracts were rich in terms of triterpenoids (oleanolic acid, ursolic acid, α/β-amyrin, uvaol, etc.), which contributed to the observed antioxidant effects. For example, Sunil et al. (2014) isolated β-amyrin from *Symplocos cochinchinensis*, and it exhibited great radical scavenging ability in the DPPH assay [37]. In another study by Karen Cardoso et al. (2020), α and β-amyrin were isolated from *Myrcianthes pungens* and they showed antioxidant effects in DPPH and β-carotene/linoleic acid bleaching assays [38]. As a structure–ability insight on amyrin derivatives, the presence of an –OH group attached at the C-3 carbon of the A ring can contribute to their antioxidant properties. Furthermore, in a study by Allouche et al. (2010), the antioxidant potential of several pentacyclic triterpenes, including uvaol, erythrodiol, oleanolic acid, and maslinic acid, was assessed; uvaol and erythrodiol demonstrated significant antioxidant activity [39].

Enzymes are potent agents for addressing significant health issues alongside their catalytic roles. Recently, most treatments have been focused on targeting key enzymes, and their inhibition might mitigate pathological symptoms [40]. Amylase and glucosidase are primary enzymes involved in carbohydrate hydrolysis, and their inhibition can regulate blood glucose levels in diabetes patients [41,42]. Cholinesterase inhibition can elevate acetylcholine levels in the synaptic cleft, enhancing cognitive function in people with Alzheimer’s disease [43]. In the pharmaceutical industry, certain compounds have been developed as enzyme inhibitors; nonetheless, the majority exhibit undesirable side effects. Therefore, it is essential to identify effective and safe inhibitors derived from natural resources. In this context, we evaluated the enzyme inhibitory capabilities of the tested extract against various enzymes, and the findings are displayed in Table 3. The tested extract exhibited AChE inhibitory effects with 2.56 mg GALAE/g, while it was not active on BChE. The tested extract inhibited the action of tyrosinase with 71.90 mg KAE/g. The amylase and glucosidase inhibition abilities were found to be 0.36 mmol ACAE/g and 1.35 mmol ACAE/g, respectively. Nowadays, one of the most remarkable and popular areas of study is skin aging, a part of the aging process, and humanity has turned to natural resources to delay aging [44]. When the anti-collagenase, anti-elastase and anti-hyaluronidase IC_50_ values of the tested extract were compared, the highest inhibitory effect (lowest IC_50_) was observed on the elastase enzyme (16.49 ± 1.6 µg/mL). The IC_50_ value for collagenase was determined as 17.18 ± 1.61 µg/mL and for hyaluronidase as 22.83 ± 2.04 µg/mL. Compared with control compounds, EGCG had a stronger inhibitory effect on collagenase (11.31 ± 2.06 µg/mL) and elastase (10.77 ± 1.85 µg/mL), while tannic acid provided significant inhibition on hyaluronidase (10.93 ± 2.11 µg/mL).

As highlighted in a recent review and research articles, the Ericaceae family provides a rich source of bioactive constituents and its health-promoting benefits include anti-inflammatory, antioxidant, anti-cancer, anti-diabetic and anti-bacterial properties [1,44,45]. Based on Table 3, it is seen that the plant is rich in total phenolic compounds and flavonoids. In particular, compounds such as chlorogenic acid, rosmarinic acid, proanthocyanidins (proanthocyanidin dimers, trimers and tetramers), catechins and myricetin and quercetin derivatives have the potential to inhibit amylase [46,47,48], cholinesterase [49] and matrix metalloproteinase enzymes and may suppress the activity of enzymes associated with skin aging [1,45,50]. It has been reported that type A oligomeric proanthocyanidins from litchi pericarp and type B oligomeric proanthocyanidins from lotus seedpod improve glucose homeostasis in diabetic mice by activating a series of enzymes related to glucose metabolism [51]. It is noteworthy that in an obesity and type 2 diabetes mouse model, oligomeric cocoa proanthocyanidins have been shown to be the most effective compounds in improving glucose tolerance and insulin resistance in comparison with monomeric or polymeric proanthocyanidins [33]. Apple extract, which is rich in proanthocyanidins, exhibits anti-obesity effects by inhibiting pancreatic lipase in vitro, while other investigated polyphenols such as chalcones, catechins and phenolic acids exhibit weak inhibitory activity on pancreatic lipase [52]. As the degree of polymerization increases, the anti-obesity activity of proanthocyanidins increases from dimers to pentamers. In addition to phenolics, the presence of triterpenoids in the tested extract can be attributed to their enzyme inhibitory effects. For example, ursolic and oleanolic acids exhibited substantial inhibitory effects in previous studies [53,54,55].

## 3. Materials and Methods

### 3.1. Plant Material

*Erica spiculifolia* aerial parts were collected at the locality “Kamen del”, Vitosha Mt. (1860 m. a.s.l., 42.61195270° N, 23.27660140° E), Bulgaria, during the beginning of flowering in July 2024 (ES1) and at the full flowering stage in August 2024 (ES2). The species taxonomic identity was confirmed by a member of our research team (D. Zheleva) according to https://www.worldfloraonline.org/ [56]. A voucher specimen was deposited at Herbarium of the Institute of Biodiversity and Ecosystem Research, Bulgarian Academy of Sciences (SOM) (Voucher specimen No. 179374). The plant material was dried at room temperature.

### 3.2. Chemicals

The chemicals were purchased from Sigma-Aldrich (Darmstadt, Germany). They were 2,2′-azino-bis(3-ethylbenzothiazoline-6-sulphonic acid (ABTS), 1,1-diphenyl-2-picrylhydrazyl (DPPH), gallic acid, rutin, caffeic acid, electric eel acetylcholinesterase (AChE) (type-VI-S, EC 3.1.1.7, Cat No:A3389), horse serum butyrylcholinesterase (BChE) (EC 3.1.1.8, Cat No:C7512), galantamine, acetylthiocholine iodide (ATChI), butyrylthiocholine chloride (BTChI) 5,5-dithio-bis(2-nitrobenzoic) acid (DTNB), tyrosinase (EC1.14.18.1, mushroom, Cat No: T3824), glucosidase (EC. 3.2.1.20, from Saccharomyces cerevisiae, Cat No: G5003), amylase (EC. 3.2.1.1, from porcine pancreas, Cat No: A3176), sodium molybdate, sodium nitrate, sodium carbonate, Folin–Ciocalteu reagent, hydrochloric acid, sodium hydroxide, trolox, ethylenediaminetetraacetate (EDTA), neocuproine, cupric chloride, ammonium acetate, ferric chloride, 2,4,6-Tris(2-pyridyl)-s-triazine (TPTZ), ammonium molybdate, ferrozine, ferrous sulphate hexahydrate, kojic acid and acarbose. All chemicals were of analytical grade. Collagenase from Clostridium histolyticum for anti-collagenase activity was supplied by Sigma Aldrich (Cat No: C0130 and EC.3.4.23.3), and the substrate N-[3-(2-Furyl)acryloyl]-Leu-Gly-Pro-Ala (Cat No: F5135) was also purchased from the same company. Elastase was supplied by Sigma Aldrich (Cat No: E1250 and EC: 3.4.21.36), and the substrate N-Succinyl-Ala-Ala-Ala-p-nitroanilide (Cat No: S4760) was also purchased from the same company. Hyaluronidase enzyme (Cat No: H3506 and EC.3.2.1.35) and hyaluronic acid (Cat No: 924474) were purchased commercially from Sigma Aldrich.

The reference standards for TLC and GC-MS were purchased from Roth (Karlsruhe, Germany) (α-amyrin, and ursolic acid methyl ester) and Sigma-Aldrich (Steinheim, Germany) (β-amyrin, uvaol, oleanolic acid, campesterol, sitosterol and stigmasterol). All solvents used for extraction and analysis (diethyl ether, methanol, chloroform) were provided by POCH (Gliwice, Poland) and were of analytical grade.

### 3.3. Sample Extraction

*E. spiculifolia* aerial parts were powdered by using a grinder (Rohnson, R-942, 220–240 V, 50/60 Hz, 200 W, Prague, Czech Republic). Powdered plant material (50 g) was extracted with 80% MeOH (1:20 w/v) by sonication (100 kHz, ultra-sound bath Biobase UC-20C, Biobase, Jinan, Shandong, China) for 15 min (×2) at room temperature. The methanol was evaporated in vacuo (40 °C) and water residues were lyophilized (lyophilizer Biobase BK-FD10P, Biobase, Jinan, Shandong, China; −65 °C) to yield 5.6 g of crude extract ES1 and 4.9 g ES2. Then, the lyophilized extracts were dissolved in 80% methanol (0.1 mg/mL) and filtered through a 0.45 μm syringe filter (Polypure II, Alltech, Lokeren, Belgium), and an aliquot (2 mL) of each solution was subjected to LC–HRMS analyses. The ES2 extract was used for in vitro antioxidant and enzymatic capacity tests.

For steroid and triterpenoid GC-MS analysis, the dried samples of *E. spiculifolia* were prepared for extraction either by separating them into flowers and leaves (for ES1) or by using the entire aerial part (a mixture of flowers, leaves and stems for ES1 and ES2). Extraction was performed with diethyl ether in Soxhlet apparatus for 10 h. The extracts were then evaporated using a vacuum rotary evaporator (Laborota 4000, Heidolph, Scientific Products, Schwabach, Germany).

### 3.4. LC-HRMS

The LC-HRMS analyses were performed as previously described [19] on a Q Exactive Plus mass spectrometer (ThermoFisher Scientific, Inc., Waltham, MA, USA) with a heated electrospray ionization (HESI-II) probe (Thermo-Scientific). The equipment was operated in negative mode within the *m*/*z* range of 150 to 1500. Chromatographic separation was achieved on a Kromasil Eter-nityXT C18 (1.8 µm, 2.1 × 100 mm) reversed-phase column at 40 °C. The LC analyses were run with a mobile phase consisting of 0.1% formic acid (A) and 0.1% formic acid in acetonitrile (B). The run time was 33 min and the flow rate was 0.3 mL/min. The used gradient elution program was as follows: 0–1 min, 0–5% B; 1–20 min, 5–30% B; 20–25 min, 30–50% B; 25–30 min, 50–70% B; 30–33 min, 70–95%; 33–34 min 95–5% B. The injection volume was 1 µL, and the flow rate was 300 µL/min. Data were processed by using the Xcalibur 4.2 (ThermoScientific, Waltham, MA, USA) instrument control/data handling software. As no standards are available for the majority of the annotated compounds, the peak area of each signal in the mass chromatogram was used to provide semi-quantitative information for comparison purposes. MZmine 2 software was applied to the UHPLC–HRMS raw files of the studied methanol–aqueous extracts for the semi-quantitative analysis. The results are expressed as the % peak area of the compound to the total peak areas of the corresponding group of secondary metabolites and all metabolites.

### 3.5. Assay for Total Phenolic and Flavonoid Contents

According to the methods specified by Zengin and Aktumsek [57], total phenolics and flavonoids were quantified. The extract was prepared at a concentration of 2 mg/mL. Gallic acid (GA) and rutin (R) were used as positive equivalents in the assays, and the results were reported as gallic acid equivalents (GAEs) and rutin equivalents. The values of the calibration curves are as follows: for the total phenolic content, absorbance = 0.268 [μg gallic acid] (R2: 0.9988; concentration range: 0–3 μg gallic acid); for total flavonoid content, absorbance = 0.1274 [μg rutin] + 0.0506 (R2: 0.9968; concentration range: 0–20 μg rutin). The experimental details are presented in the Supplementary Materials.

### 3.6. Assays for In Vitro Antioxidant Capacity

According to the methods provided by Zengin et al. [58], antioxidant tests were executed. The extract was prepared at concentrations of 0.1–2 mg/mL. The DPPH, ABTS radical scavenging, CUPRAC and FRAP results were expressed as milligrams of Trolox equivalents (TE) per gram of extract. The antioxidant potential determined by the phosphomolybdenum (PBD) assay was presented in millimoles of Trolox equivalents (TE) per gram of extract. Metal chelating activity (MCA) was calculated as milligrams of disodium edetate equivalents (EDTAEs) per gram of extract. The experimental details are presented in the Supplementary Materials.

### 3.7. Inhibitory Effects Against Some Key Enzymes

Enzyme inhibition experiments on the samples were carried out following established protocols [58]. The extract was prepared at a concentration of 0.1–2 mg/mL. Amylase and glucosidase inhibition were expressed as acarbose equivalents (ACAEs) per gram of extract, while acetylcholinesterase (AChE) and butyrylcholinesterase (BChE) inhibition were evaluated as milligrams of galanthamine equivalents (GALAEs) per gram of extract. Tyrosinase inhibition was evaluated in milligrams of kojic acid equivalents (KAEs) per gram of extract. Lipase inhibition was measured as the equivalent of orlistat (OE) per gram of extract. The experimental details are presented in the Supplementary Materials.

The IC_50_ value was determined for the collagenase, elastase and hyaluronidase inhibitory activity experiment by increasing the concentration of the extract from 62.5 μg/mL to 1000 μg/mL. Tannic acid was selected to test hyaluronidase enzyme inhibition. On the other hand, epigallocatechin gallate (EGCG) was used as the positive control for collagenase and elastase. The negative control was distilled water. The experimental details are presented in the Supplementary Materials.

### 3.8. Fractionation of Extracts by Thin-Layer Adsorption Chromatography (TLC)

The 20 cm × 20 cm glass plates were defatted with acetone and manually coated with a 0.25 mm layer of silica gel 60G (Roth, Karlsruhe, Germany). Dried extracts were dissolved in appropriate volumes of diethyl ether and applied linearly onto the silica gel using a glass capillary column. Standard solutions of oleanolic acid, sitosterol and α-amyrin were applied on the side of the plate parallel to the extract. The plates were developed in chromatographic chambers in the solvent system CHCl_3_/MeOH (97:3, *v/v*).

The individual fractions were localized on the plates by comparison with standards, visualized by spraying the relevant part of the plate with 50% H_2_SO_4_, followed by heating with a hot air stream. The gel was divided into two fractions: (i) free (non-esterified) steroids and neutral triterpenoids (alcohols, aldehydes and ketones) and (ii) triterpenoid acids. Fractions were eluted from the gel in diethyl ether using at least 10 volumes of the solvent relative to the volume of the scrapped gel. The fractions containing free neutral triterpenes and steroids (*R*_F_ 0.3–0.9) were analyzed directly by GC-MS, while the fractions containing triterpene acids (*R*_F_ 0.2–0.3) were first methylated with diazomethane.

### 3.9. Methylation of Triterpenoid Acids

Nitrosomethylurea (2.06 g) was added to a mixture of 100 mL of diethyl ether and 6 mL of 25% aqueous KOH; the organic layer was washed with water (3 × 50 mL) and separated from the aqueous layer. Samples containing triterpenoid acids were dissolved in 5 mL of the obtained solution of diazomethane in diethyl ether and held at 2 °C for 24 h.

### 3.10. Identification and Quantification of Steroids and Triterpenoids by Gas Chromatography–Mass Spectrometry (GC-MS)

An Agilent Technologies 7890A gas chromatograph equipped with a 5975C mass spectrometric detector was used for both qualitative and quantitative analyses. Samples dissolved in diethyl ether/methanol (5:1, *v*/*v*) were applied (in volumes of 1–4 μL) using a 1:10 split injection. All samples were analyzed in triplicate. The column used was 30 m − 0.25 mm, i.d., 0.25 μm, HP-5MS UI (Agilent Technologies, Santa Clara, CA, USA). Helium was used as the carrier gas at a flow rate of 1 mL/min. Separation was carried out using the following temperature program: an initial temperature of 160 °C held for 2 min, then increased to 280 °C at a rate of 5 °C/1 min and the final temperature of 280 °C held for an additional 44 min.

The other employed parameters were as follows: inlet and FID (flame ionization detector) temperature of 290 °C; MS transfer line temperature of 275 °C; quadrupole temperature of 150 °C; ion source temperature of 230 °C; EI 70 eV; *m*/*z* range of 33–500; FID gas (H_2_) flow of 30 mL·min^−1^ (hydrogen generator); and air flow of 400 mL·min^−1^. Individual compounds were identified by comparing their mass spectra with library data from Wiley 9th ED. and NIST 2008 Lib. SW Version 2010, or previously reported data, as well as via comparison of their retention times and corresponding mass spectra with those of authentic standards when available. Quantitation was performed using an external standard method based on calibration curves determined for compounds belonging to representative triterpenoid classes: α-amyrin for triterpene alcohols, oleanolic acid methyl ester for triterpene acid methyl esters and sitosterol for steroids. Chromatograms were processed with the use of Agilent G1701EA GC/MSD ChemStation software E.02.01 2010.

### 3.11. Statistical Analysis

The experiments for the evaluation of total phenolic and flavonoid contents and antioxidant and enzyme inhibitory capacity were performed in triplicate and the results are presented as the mean and standard deviation. GraphPad 9.1 was used to evaluate the obtained results [59,60].

The IC50 value was determined for each enzyme inhibitory activity experiment by increasing the concentration of different extracts from 62.5 ug/mL to 1000 ug/mL. Tannic acid was selected to test hyaluronidase enzyme inhibition. On the other hand, epigallocatechin gallate was used as the positive control for collagenase and elastase. The negative control was distilled water.

## 4. Conclusions

Herein, more than 100 secondary metabolites, including 11 acylquinic acids, 39 oligomeric proanthocyanidins (PACs) and 51 flavonoids (flavones, flavonols and flavanols), were annotated/dereplicated in Balkan heath methanol–aqueous extracts for the first time. HRMS/MS fragmentation patterns of proanthocyanidin di-, tri-, tetra- and pentamers were proposed. Comprehensive profiling of PACs revealed mainly hybrid A, B-types. Triterpenoid acids dominated the apolar extract composition, reaching up to 80% of the total triterpenoids. It is noteworthy that ursane-type triterpenoids were the most abundant among the triterpenoids, evidenced by the ursolic acid, 3-oxo-ursolic acid, α-amyrin, uvaol and ursolic aldehyde contents. According to this first attempt to delineate the chemical profile of the polar and apolar extracts, the presence of proanthocyanidins A, B-type and ursane-type triterpenoids (ursolic acid, α-amyrin, α-amyrin acetate, uvaol) could be considered to hold chemophenetic importance for the species. Chlorogenic acid, tetra- and pentamer proanthocyanidins, catechin and quercetin–glycosides, along with α- and β-amyrin, uvaol and erythrodiol, hold significance for the strong antioxidant capacity in DPPH, ABTS, CUPRAC and FRAP assays. Indeed, polyphenols and triterpenoids are the main classes in the Balkan heath with a marked impact on cholinesterases and tyrosinase.

Proanthocyanidins with a higher degree of polymerization (penta-, hexa- and heptamers) together with acylquinic acids accounted for the α-glucosidase inhibitory activity. In addition to evoking an antioxidant response, the Balkan heath methanol–aqueous extract exerted in vitro inhibitory activity towards key enzymes of carbohydrate and lipid metabolism and melanin biosynthesis, which evokes further interest in the plant species as a potential candidate for corresponding issues. The present study argues for in vivo experiments with *E. spiculifolia* extracts designed to assess antioxidant and lipid status and glucose homeostasis in metabolic disorders, as well as conditions associated with skin aging.

## Figures and Tables

**Table 1:** Secondary metabolites in *E. spiculifolia* methanol–aqueous extracts (aerial parts).

№	Identified/Tentatively Annotated Compound	Molecular Formula	Exact Mass [M-H]^−^	t_R_ (min)	Distribution
**Acylquinic acids**					
1.	neochlorogenic acid ^a^	C_16_H_18_O_9_	353.0878	2.36	ES1, ES2
2.	chlorogenic acid ^a^	C_16_H_18_O_9_	353.0878	3.18	ES1, ES2
3.	4-caffeoylquinic acid	C_16_H_18_O_9_	353.0878	3.35	ES1, ES2
4.	3-feruloylquinic acid	C_17_H_20_O_9_	367.1035	3.44	ES1, ES2
5.	4-*p*-coumaroylquinic acid	C_16_H_18_O_8_	337.0929	3.79	ES1, ES2
6.	5-caffeoylquinic acid isomer	C_16_H_18_O_9_	353.0878	3.89	ES1, ES2
7.	5-*p*-coumaroylquinic acid	C_16_H_18_O_8_	337.0929	3.95	ES1, ES2
8.	4-*p*-coumaroylquinic acid isomer	C_16_H_18_O_8_	337.0929	4.03	ES1, ES2
9.	5-feruloylquinic acid	C_17_H_20_O_9_	367.1035	4.39	ES1, ES2
10.	5-*p*-coumaroylquinic acid isomer	C_16_H_18_O_8_	337.0929	4.61	ES1, ES2
11.	3, 5-dicaffeoylquinic acid	C_25_H_24_O_12_	515.1195	5.85	ES1, ES2
12.	rosmarinic acid ^a^	C_18_H_16_O_8_	359.0772	6.33	ES1, ES2
**Proanthocyanidin oligomers (PACs)**					
13.	proanthocyanidin tetramer A, B-type	C_60_H_48_O_24_	1151.2463	2.72	ES1, ES2
14.	proanthocyanidin dimer B-type	C_30_H_26_O_12_	577.1351	2.77	ES1, ES2
15.	proanthocyanidin dimer B-type	C_30_H_26_O_12_	577.1351	2.94	ES1, ES2
16.	proanthocyanidin pentamer A, B-type	C_75_H_60_O_30_	719.1509 [M-2H]^2−^	3.27	ES1
17.	proanthocyanidin trimer B-type	C_45_H_38_O_18_	865.1985	3.25	ES1
18.	proanthocyanidin dimer B-type	C_30_H_26_O_12_	577.1351	3.45	ES1, ES2
19.	proanthocyanidin pentamer A, B-type	C_75_H_60_O_30_	719.1509 [M-2H]^2−^	3.47	ES1, ES2
20.	proanthocyanidin tetramer A, B-type	C_60_H_48_O_24_	1151.2463	3.53	ES1, ES2
21.	proanthocyanidin trimer B-type	C_45_H_38_O_18_	865.1985	3.57	ES1, ES2
22.	proanthocyanidin dimer B-type	C_30_H_26_O_12_	577.1351	3.58	ES1, ES2
23.	proanthocyanidin tetramer B-type	C_60_H_50_O_24_	576.1273 [M-2H]^2−^	3.79	ES1, ES2
24.	proanthocyanidin pentamer A, B-type	C_75_H_60_O_30_	719.1512 [M-2H]^2−^	3.84	ES1, ES2
25.	proanthocyanidin trimer A, B-type	C_45_H_36_O_18_	863.1829	3.88	ES1, ES2
26.	proanthocyanidin trimer A, B-type	C_45_H_36_O_18_	863.1829	4.11	ES1, ES2
27.	proanthocyanidin trimer B-type	C_45_H_38_O_18_	865.1985	4.18	ES1, ES2
28.	proanthocyanidin hexamer A, B-type	C_90_H_72_O_36_	863.1829 [M-2H]^2−^	4.27	ES1
29.	proanthocyanidin tetramer A, B-type	C_60_H_48_O_24_	1151.2462	4.32	ES1
30.	proanthocyanidin dimer B-type	C_30_H_26_O_12_	577.1351	4.38	ES1, ES2
31.	proanthocyanidin tetramer B-type	C_60_H_50_O_24_	1153.2619	4.38	ES1, ES2
32.	proanthocyanidin pentamer B-type	C_75_H_62_O_30_	720.1590 [M-2H]^2−^	4.46	ES1, ES2
33.	proanthocyanidin trimer B-type	C_45_H_38_O_18_	865.1985	4.47	ES1, ES2
34.	proanthocyanidin pentamer B-type	C_75_H_62_O_30_	720.1590 [M-2H]^2−^	4.59	ES1, ES2
35.	proanthocyanidin tetramer B-type	C_60_H_50_O_24_	576.1273 [M-2H]^2−^	4.60	ES1, ES2
36.	proanthocyanidin pentamer A, B-type	C_75_H_60_O_30_	719.1512 [M-2H]^2−^	4.68	ES1, ES2
37.	proanthocyanidin dimer A-type	C_30_H_24_O_12_	575.1195	4.74	ES1, ES2
38.	proanthocyanidin tetramer A, B-type	C_60_H_48_O_24_	575.1195 [M-2H]^2−^	4.83	ES1, ES2
39.	proanthocyanidin heptamer A, B-type	C_105_H_86_O_42_	1008.7241 [M-2H]^2−^	4.90	ES1, ES2
40.	proanthocyanidin pentamer A, B-type	C_75_H_60_O_30_	719.1512 [M-2H]^2−^	4.97	ES1, ES2
41.	proanthocyanidin tetramer A, B-type	C_60_H_48_O_24_	575.1195 [M-2H]^2−^	4.97	ES1, ES2
42.	proanthocyanidin pentamer A, B-type	C_75_H_60_O_30_	719.1512 [M-2H]^2−^	5.05	ES1, ES2
43.	proanthocyanidin trimer B-type	C_45_H_38_O_18_	865.1985	5.17	ES1, ES2
44.	proanthocyanidin dimer B-type	C_30_H_26_O_12_	577.1351	5.20	ES1, ES2
45.	proanthocyanidin pentamer A-type	C_75_H_58_O_30_	718.1434 [M-2H]^2−^	5.23	ES1, ES2
46.	proanthocyanidin tetramer A, B-type	C_60_H_48_O_24_	575.1195 [M-2H]^2−^	5.36	ES1, ES2
47.	proanthocyanidin pentamer A, B-type	C_75_H_60_O_30_	1439.3096	5.36	ES1, ES2
48.	Proanthocyanidin trimer A, B-type	C_45_H_36_O_18_	863.1829	5.58	ES1, ES2
49.	proanthocyanidin dimer A-type	C_30_H_24_O_12_	575.1195	5.83	ES1, ES2
50.	proanthocyanidin tetramer A, B-type	C_60_H_46_O_24_	1149.2306	6.22	ES1, ES2
51.	proanthocyanidin pentamer A-type	C_75_H_58_O_30_	718.1434 [M-2H]^2−^	6.36	ES1, ES2
**Flavonoids**					
52.	gallocatechin	C_15_H_14_O_7_	305.0667	1.79	ES1, ES2
53.	(+) catechin	C_15_H_14_O_6_	289.0718	3.12	ES1, ES2
54.	naringenin 6, 8 di*C*-hexoside	C_27_H_32_O_15_	595.1678	3.64	ES1, ES2
55.	eryodictiol *O*-hexoside 1	C_21_H_22_O_11_	449.1089	3.73	ES1, ES2
56.	gossypetin *O*-hexoside 1	C_21_H_20_O_13_	479.0831	3.90	ES1, ES2
57.	epicatechin	C_15_H_14_O_6_	289.0718	3.90	ES1, ES2
58.	gossypetin *O*-rutinoside	C_27_H_30_O_17_	625.1410	4.01	ES1, ES2
59.	eryodictiol *O*-hexoside 1	C_21_H_22_O_11_	449.1089	4.02	ES1, ES2
60.	gossypetin *O*-hexoside 2	C_21_H_20_O_13_	479.0831	4.04	ES1, ES2
61.	gossypetin *O*-pentoside 1	C_20_H_18_O_12_	449.0725	4.18	ES1, ES2
62.	gossypetin *O*-pentoside 2	C_20_H_18_O_12_	449.0725	4.24	ES1, ES2
63.	galangin methyl ether *O*-hexoside	C_23_H_24_O_12_	491.1195	4.36	ES1, ES2
64.	quercetin *O*-dihexoside	C_27_H_30_O_17_	625.1410	4.40	ES1, ES2
65.	saponarin ^a^	C_27_H_30_O_15_	593.1512	4.40	ES1, ES2
66.	gossypetin *O*-pentoside 3	C_20_H_18_O_12_	449.0725	4.50	ES1, ES2
67.	myricetin O-hexoside 1	C_21_H_20_O_13_	479.0831	4.51	ES1, ES2
68.	myricetin *O*-hexoside 2	C_21_H_20_O_13_	479.0831	4.59	ES1, ES2
69.	luteolin *O*-hexosyl-*O*-hexuronide	C_27_H_28_O_17_	623.1254	4.62	ES1, ES2
70.	myricetin *O*-pentoside 1	C_20_H_18_O_12_	449.0725	4.98	ES1, ES2
71.	myricetin *O*-pentoside 2	C_20_H_18_O_12_	449.0725	5.03	ES1, ES2
72.	rutin ^a^	C_27_H_30_O_16_	609.1464	5.08	ES1, ES2
73.	quercetin *O*-pentosylhexoside	C_26_H_28_O_16_	595.1305	5.11	ES1
74.	isoquercitrin ^a^	C_21_H_20_O_12_	463.0886	5.18	ES1, ES2
75.	quercetin *O*-hexuronide	C_21_H_18_O_13_	477.0675	5.22	ES1, ES2
76.	luteolin 7-*O*-rutinside ^a^	C_27_H_30_O_15_	593.1512	5.26	ES1, ES2
77.	hyperoside ^a^	C_21_H_20_O_12_	463.0887	5.29	ES1, ES2
78.	luteolin *O*-hexuronide	C_21_H_18_O_12_	461.0725	5.39	ES1, ES2
79.	luteolin 7-*O*-glucoside ^a^	C_21_H_20_O_11_	447.0933	5.39	ES1, ES2
80.	luteolin *O*-deoxyhexosyl-*O*-hexoside	C_27_H_30_O_15_	593.1512	5.44	ES1, ES2
81.	quercetin 3-*O*-pentoside 1	C_20_H_18_O_11_	433.0776	5.52	ES1, ES2
82.	quercetin 3-O-pentoside 2	C_20_H_18_O_11_	433.0776	5.63	ES1, ES2
83.	kaempferol 3-*O*-rutinoside ^a^	C_27_H_30_O_15_	593.1512	5.63	ES1, ES2
84.	quercetin *O*-pentoside 3	C_20_H_18_O_11_	433.0776	5.74	ES1, ES2
85.	isorhamnetin 3-*O*-rutinoside ^a^	C_28_H_32_O_16_	623.1618	5.80	ES1, ES2
86.	quercitrin ^a^	C_21_H_20_O_11_	447.0933	5.93	ES1, ES2
87.	Isorhamnetin 3-*O*-glucoside ^a^	C_22_H_22_O_12_	477.1044	6.02	ES1, ES2
88.	luteolin *O*-hexoside	C_21_H_20_O_11_	447.0933	6.06	ES1, ES2
89.	apigenin 7-*O*-glucoside ^a^	C_21_H_20_O_10_	431.0983	6.09	ES1, ES2
90.	apigenin *O*-hexuronide	C_21_H_18_O_11_	445.0776	6.13	ES1, ES2
91.	gossypetin	C_15_H_10_O_8_	317.0303	6.19	ES1, ES2
92.	chrysoeriol *O*-hexoside	C_22_H_22_O_11_	461.1089	6.31	ES1, ES2
93.	quercetin *O*-caffeoylhexoside	C_30_H_26_O_15_	625.1199	6.44	ES1, ES2
94.	kaempferol *O*-deoxyhexoside	C_21_H_20_O_11_	431.0983	6.61	ES1, ES2
95.	isorhamnetin *O*-deoxyhexoside	C_22_H_22_O_11_	461.1089	6.75	ES1, ES2
96.	luteolin *O*-acetylhexoside	C_23_H_22_O_12_	489.1038	6.87	ES1, ES2
97.	quercetin *O*-pentoside 4	C_20_H_18_O_11_	433.0776	7.04	ES1, ES2
98.	luteolin ^a^	C_15_H_10_O_6_	285.0405	7.58	ES1, ES2
99.	quercetin ^a^	C_15_H_10_O_7_	301.0354	7.62	ES1, ES2
100.	apigenin ^a^	C_15_H_10_O_5_	269.0457	8.63	ES1, ES2
101.	kaempferol ^a^	C_15_H_9_O_7_	285.0406	8.83	ES1, ES2
102.	hispidulin (scutellarein-6-methyl ether) ^a^	C_16_H_12_O_6_	299.0563	8.85	ES1, ES2
103.	quercetin O-cinnamoylhexoside1	C_30_H_26_O_13_	593.1301	8.86	ES1, ES2
104.	chrysoeriol ^a^	C_16_H_12_O_6_	299.0562	8.93	ES1, ES2
105.	quercetin O-cinnamoylhexoside2	C_30_H_26_O_13_	593.1301	9.13	ES1, ES2

^a^ identified by comparison with an authentic standard; ES1-*E. spiculifolia* collected in July; ES2-*E. spiculifolia* collected in August.

**Table 2:** The content of steroids and triterpenoids (µg/g dry weight) in *E. spiculifolia* diethyl ether extracts.

Compound	Flowers (ES1)	Leaves (ES2)	Aerial Part (ES1)	Aerial Part (ES2)
***steroids***				
campesterol	62.48 ± 4.42	32.31 ± 3.05	22.39 ± 2.11	17.28 ± 2.04
sitosterol	625.86 ± 58.04	652.60 ± 62.18	804.05 ± 76.60	728.58 ± 70.64
sitostanol	45.12 ± 5.36	50.04 ± 5.58	69.02 ± 6.80	62.18 ± 5.92
tremulone	71.88 ± 7.04	73.33. ± 6.95	97.45 ± 10.03	91.96 ± 9.28
24-methylenecycloartanol	142.66 ± 12.86	127.20 ± 10.64	160.75 ± 15.50	148.01 ± 12.55
*sum*	*948.00*	*935.48*	*1084.64*	*1048.02*
***neutral triterpenoids***				
α-amyrin	2732.80 ± 252.64	3297.49 ± 310,50	3333.86 ± 296.12	3291.04 ± 315.68
α-amyrenone	1305.13 ± 108.90	1436.92 ± 140.24	1890.60 ± 166.48	1670.49 ±150.05
α-amyrin acetate	175.12 ± 15.48	151.44 ± 14.80	193.21 ± 20.50	216.66 ± 20.82
β-amyrin	1339.84 ± 128.16	1326.26 ± 122.38	1428.05 ± 140.10	1603.28 ± 155.86
germanicol	88.09 ± 7.50	75.16 ± 6.94	91.50 ± 9.01	84.41 ± 7.55
taraxasterol	94.71 ± 9.33	90.44 ± 8.86	98.32 ± 9.64	84.76 ±8.06
erythrodiol	18.45 ± 1.70	19.89 ± 2.05	23.12 ± 2.26	20.42 ± 2.18
uvaol	47.59 ± 4.11	50.21 ± 4.95	75.32 ± 7.18	62.41 ± 6.03
oleanolic aldehyde	91.01 ± 8.15	75.44 ± 7.08	98.32 ± 10.46	95.48 ± 9.60
ursolic aldehyde	487.05 ± 44.10	422.91 ± 38.15	468.39 ± 42.50	455.02 ± 41.98
*sum*	*6379.79*	*6946.16*	*7700.69*	*7583.97*
***triterpenoid acids***				
olean-2,12-dien-28-oic acid	115.84 ± 10.62	132.87 ± 12.85	106.73 ± 9.40	126.39 ± 10.55
ursa-2,12-dien-28-oic acid	471.10 ± 45.38	500.27 ± 48.05	579.38 ± 55.14	679.12 ± 63.08
3-oxo-oleanolic acid	65.41 ± 6.55	86.98 ± 7.92	96.63 ± 9.01	105.89 ± 9.45
3-oxo-ursolic acid	493.35 ± 45.70	693.93 ± 66.05	625.39 ± 58.91	703.78 ± 66.22
oleanolic acid	4783.35 ± 420.50	6138.27 ± 562.33	4717.10 ± 458.02	5508.94 ± 533.46
ursolic acid	19,428.66 ± 1859.12	32,214.84 ± 3006.28	20,911.64 ± 1894.92	21,546.75 ± 1909.15
unidentified acid *	3054.65 ± 282.01	5672.02 ± 508.14	4825.00 ± 456.86	5558.69 ± 527.50
*sum*	*30,412.36*	*45,439.18*	*31,861.87*	*34,229.56*
**Total**	**37,740.15**	**53,320.82**	**40,647.20**	**42,861.55**

The results are in reference to dry weight and expressed as the mean ± SD of three samples. *—compound tentatively identified; ES1—*E. spiculifolia* collected in July; ES2—*E. spiculifolia* collected in August.

**Table 3:** Total bioactive compounds and antioxidant and enzyme inhibitory properties of *E. spiculifolia* methanol–aqueous extract ES2.

***Total Bioactive Compounds***	
Total phenolic content (mg GAE/g)	83.85 ± 0.89
Total flavonoid content (mg RE/g)	78.91 ± 0.41
***Antioxidant Properties***	
DPPH scavenging ability (mg TE/g)	540.01 ± 9.68
ABTS scavenging ability (mg TE/g)	639.11 ± 8.51
CUPRAC (mg TE/g)	869.22 ± 25.02
FRAP (mg TE/g)	660.32 ± 17.15
Metal chelating (mg EDTAE/g)	15.57 ± 1.44
Phosphomolybdenum (mmol TE/g)	2.52 ± 0.03
***Enzyme Inhibitory Properties***	
AChE inhibition (mg GALAE/g)	2.56 0.06
BChE inhibition (mg GALAE/g)	n.a.
Tyrosinase inhibition (mg KAE/g)	71.90 ± 1.50
Amylase inhibition (mmol ACAE/g)	0.36 ± 0.002
Glucosidase inhibition (mmol ACAE/g)	1.35 ± 0.004
Lipase inhibition (mg OE/g)	53.26 ± 4.44
Collagenase inhibition (IC_50_ μg/mL)	17.18 ± 1.61
Elastase inhibition (IC_50_ μg/mL)	16.49 ± 1.60
Hyaluronidase inhibition (IC_50_ μg/mL)	22.83 ± 2.04

Values are reported as the mean ± SD of three parallel measurements. GAE: gallic acid equivalent; RE: rutin equivalent; TE: Trolox equivalent; EDTAE: EDTA equivalent; GALAE: galanthamine equivalent; KAE: kojic acid equivalent; ACAE: acarbose equivalent; OE: orlistat equivalent; n.a.: not active.

## Data Availability

The original contributions presented in this study are included in the article; further inquiries can be directed to the corresponding author.

## Supplementary Materials

The supporting information can be downloaded at https://www.mdpi.com/article/10.3390/plants14111648/s1. (See Refs. [59,60,61,62,63,64]).

## Abbreviations

ABTS	2,2′-azino-bis(3-ethylbenzothiazoline-6-sulfonic acid)
ACAE	Acarbose equivalent
ACAs	Acylquinic acids
AChE	Acetylcholinesterase
BChE	Butyrylcholinesterase
C	(Epi)catechin moiety
CE	Catechin equivalent
CUPRAC	CUPric Reducing Antioxidant Capacity
diAQAs	Diacylquinic acids
DPPH	2,2-Diphenyl-1-picrylhydrazyl
EDTAE	Ethylenediaminetetraacetic acid equivalent
EGCG	Epigallocatechin galate
ES1	Plant material from the beginning of flowering in July
ES2	Plant material from the full flowering stage in August
FRAP	Ferric Reducing Antioxidant Power
GAE	Gallic acid equivalent
GALAE	Galanthamine equivalent
GC-MS	Gas chromatography–mass spectrometry
HRF	Heterocyclic ring fission
IC_50_	The concentration required for 50% inhibition
KAE	Kojic acid equivalent
LC-HRMS	Liquid chromatography–high-resolution mass spectrometry
LC-MS	Liquid chromatography–mass spectrometry
monoAQAs	Monoacylquinic acids
Nrf2	Nuclear factor erythroid 2-related factor
OE	Orlistat equivalent
PACs	Proanthocyanidins
QM	Quinine methane or interflavan fission
RDA	Retro–Diels–Alder fission
RE	Rutin equivalent
SD	Standard deviation
TE	Trolox equivalent
TLC	Thin-layer adsorption chromatography
