# Supplementary material for: Erica spiculifolia Salisb. (Balkan Heath): A Focus on Metabolic Profiling and Antioxidant and Enzyme Inhibitory Properties
Source: Plants (Basel). 2025 May 28;14(11):1648. doi: 10.3390/plants14111648 (PMC12157037; doi:10.3390/plants14111648)
Supplement: Supplementary file 1 [file plants-14-01648-s001.zip › plants-3643880-supplementary.pdf]

Erica spiculifolia Salisb. (Balkan heath): Focus on Metabolic Profiling and Antioxidant and Enzyme Inhibitory Properties

Reneta Gevrenova[1] *, Anna Szakiel[2], Cezary Pączkowski[2], Gokhan Zengin[3], Inci Kurt-Celep[4], Alexandra Stefanova[5] and Dimitrina Zheleva-Dimitrova[1]

[1]Department of Pharmacognosy, Faculty of Pharmacy, Medical University-Sofia, 1000 Sofia, Bulgaria; rgevrenova@pharmfac.mu-sofia.bg (R.G.); dzheleva@pharmfac.mu-sofia.bg (D.Z)
[2]Department of Plant Biochemistry, Institute of Biochemistry, Faculty of Biology, University of Warsaw, 1 Miecznikowa Street, 02-096, Warsaw, Poland; a.szakiel@uw.edu.pl (A.S.); c.paczkowski@uw.edu.pl (C.P.)
[3]Physiology and Biochemistry Research Laboratory, Department of Biology, Science Faculty, Selcuk University, Konya 42130, Turkey; gokhanzengin@selcuk.edu.tr (G.Z.)
[4]İstanbul Okan University, Faculty of Pharmacy, Department of Biotechnology, 34940, Tuzla, İstanbul, Turkey, inci.celep@okan.edu.tr (IKC)
[5]Department of Pharmacology, Pharmacotherapy and Toxicology, Faculty of Pharmacy, Medical University-Sofia, Sofia 1000, Bulgaria; astefanova22@gmail.com (A.S.)

Supplemental material

Assays for Total Phenolic and Flavonoid Contents

The total phenolic content was determined by employing the methods given in the literature with some modification. Sample solution (50 µL) was mixed with diluted Folin–Ciocalteu reagent (100 µL, 1:9, v/v) and shaken vigorously. After 3 min, Na_2CO_3 solution (75 µL, 1%) was added and the sample absorbance was read at 760 nm after a 2 h incubation at room temperature. The total phenolic content was expressed as milligrams of gallic acid equivalents (mg GAE/g extract)[1].

The total flavonoid content was determined using the $AlCl_3$ method. Briefly, sample solution (100 µL) was mixed with the same volume of aluminum trichloride (2%) in methanol. Similarly, a blank was prepared by adding sample solution (100 µL) to methanol (100 µL) without $AlCl_3$. The sample and blank absorbances were read at 415 nm after a 10 min incubation at room temperature. The absorbance of the blank was subtracted from that of the sample. Rutin was used as a reference standard and the total flavonoid content was expressed as milligrams of rutin equivalents (mg RE/g extract) [1].

Determination of Antioxidant and Enzyme Inhibitory Effects

Antioxidant (DPPH and ABTS radical scavenging, reducing power (CUPRAC and FRAP), phosphomolybdenum and metal chelating (ferrozine method)) and enzyme inhibitory activities (cholinesterase (Elmann's method), tyrosinase (dopachrome method), α-amylase (iodine/potassium iodide method), α -glucosidase (chromogenic PNPG method) and pancreatic lipase (*p*-nitrophenyl butyrate (p-NPB) method) were determined using the methods previously described by Uysal et al. [1] and Grochowski et al. [2]

For the DPPH (1,1-diphenyl-2-picrylhydrazyl) radical scavenging assay: Sample solution (50 µL) was added to 150 µL of a 0.004% methanol solution of DPPH. The sample absorbance was read at 517 nm after a 30 min incubation at room temperature in the dark. DPPH radical scavenging activity was expressed as milligrams of trolox equivalents (mg TE/g extract).

For ABTS (2,2′-azino-bis(3-ethylbenzothiazoline) 6-sulfonic acid) radical scavenging assay: Briefly, ABTS+ was produced directly by reacting 7 mM ABTS solution with 2.45 mM potassium persulfate and allowing the mixture to stand for 12–16 in the dark at room temperature. Prior to beginning the assay, ABTS solution was diluted with methanol to an absorbance of 0.700 ± 0.02 at 734 nm. Sample solution (25 μL) was added to ABTS solution (200 μL) and mixed. The sample absorbance was read at 734 nm after a 30 min incubation at room temperature. The ABTS radical scavenging activity was expressed as milligrams of trolox equivalents (mg TE/g extract).

For CUPRAC (cupric ion reducing activity) activity assay: Sample solution was added to premixed reaction mixture containing $CuCl_2$ (10 mM), neocuproine (7.5 mM) and NH_4Ac buffer (1 M, pH 7.0). Similarly, a blank was prepared by adding sample solution (25 μL) to premixed reaction mixture (200 μL) without $CuCl_2$. Then, the sample and blank absorbances were read at 450 nm after a 30 min incubation at room temperature. The absorbance of the blank was subtracted from that of the sample. CUPRAC activity was expressed as milligrams of trolox equivalents (mg TE/g extract).

For FRAP (ferric reducing antioxidant power) activity assay: Sample solution (25 μL) was added to premixed FRAP reagent (200 μL) containing acetate buffer (0.3 M, pH 3.6), 2,4,6-tris(2-pyridyl)-S-triazine (TPTZ) (10 mM) in 40 mM HCl and ferric chloride (20 mM) in a ratio of 10:1:1 (v/v/v). Then, the sample absorbance was read at 593 nm after a 30 min incubation at room temperature. FRAP activity was expressed as milligrams of trolox equivalents (mg TE/g extract).

For phosphomolybdenum method: Sample solution was combined with 3 mL of reagent solution (0.6 M sulfuric acid, 28 mM sodium phosphate and 4 mM ammonium molybdate). The sample absorbance was read at 695 nm after a 90 min incubation at 95 °C. The total antioxidant capacity was expressed as millimoles of trolox equivalents (mmol TE/g extract).

For metal chelating activity assay: Briefly, sample solution (100 μL) was added to $FeCl_2$ solution (50 μL, 2 mM). The reaction was initiated by the addition of 5 mM ferrozine (100 μL). Similarly, a blank was prepared by adding sample solution (100 μL) to $FeCl_2$ solution (50 μL, 2 mM) and water (100 μL) without ferrozine. Then, the sample and blank absorbances were read at 562 nm after 10 min incubation at room temperature. The absorbance of the blank was sub-tracted from that of the sample. The metal chelating activity was expressed as milligrams of EDTA (disodium edetate) equivalents (mg EDTAE/g extract).

For Cholinesterase (ChE) inhibitory activity assay: Sample solution (was mixed with DTNB (5,5-dithio-bis(2-nitrobenzoic) acid, Sigma, St. Louis, MO, USA) (125 μL) and AChE (acetylcholinesterase (Electric ell acetylcholinesterase, Type-VI-S, EC 3.1.1.7,Sigma)), or BChE (butyrylcholinesterase (horse serum butyrylcholinesterase, EC 3.1.1.8, Sigma)) solution (25 μL) in Tris–HCl buffer (pH 8.0) in a 96-well microplate and incubated for 15 min at 25 °C. The reaction was then initiated with the addition of acetylthiocholine iodide (ATCI, Sigma) or butyrylthiocholine chloride (BTCl, Sigma) (25 μL). Similarly, a blank was prepared by adding sample solution to all reaction reagents without enzyme (AChE or BChE) solution. The sample and blank absorbances were read at 405 nm after 10 min incubation at 25 °C. The absorbance of the blank was subtracted from that of the sample and the cholinesterase inhibitory activity was expressed as galanthamine equivalents (mgGALAE/g extract).

For Tyrosinase inhibitory activity assay: Sample solution was mixed with tyrosinase solution (40 μL, Sigma) and phosphate buffer (100 μL, pH 6.8) in a 96-well microplate and incubated for 15 min at 25 °C. The reaction was then initiated with the addition of L-DOPA (40 μL, Sigma). Similarly, a blank was prepared by adding sample solution to all reaction reagents without enzyme (tyrosinase) solution. The

sample and blank absorbances were read at 492 nm after a 10 min incubation at 25 °C. The absorbance of the blank was subtracted from that of the sample and the tyrosinase inhibitory activity was expressed as kojic acid equivalents (mgKAE/g extract).

For α-amylase inhibitory activity assay: Sample solution was mixed with α-amylase solution (ex-porcine pancreas, EC 3.2.1.1, Sigma) (50 µL) in phosphate buffer (pH 6.9 with 6 mM sodium chloride) in a 96-well microplate and incubated for 10 min at 37 °C. After pre-incubation, the reaction was initiated with the addition of starch solution (50 µL, 0.05%). Similarly, a blank was prepared by adding sample solution to all reaction reagents without enzyme (α-amylase) solution. The reaction mixture was incubated 10 min at 37 °C. The reaction was then stopped with the addition of HCl (25 µL, 1 M). This was followed by addition of the iodine-potassium iodide solution (100 µL). The sample and blank absorbances were read at 630 nm. The absorbance of the blank was subtracted from that of the sample and the α-amylase inhibitory activity was expressed as acarbose equivalents (mmol ACE/g extract).

For α-glucosidase inhibitory activity assay: Sample solution was mixed with glutathione (50 µL), α-glucosidase solution (from Saccharomyces cerevisiae, EC 3.2.1.20, Sigma) (50 µL) in phosphate buffer (pH 6.8) and PNPG (4-N-trophenyl-α-D-glucopyranoside, Sigma) (50 µL) in a 96-well microplate and incubated for 15 min at 37 °C. Similarly, a blank was prepared by adding sample solution to all reaction reagents without enzyme (α-glucosidase) solution. The reaction was then stopped with the addition of sodium carbonate (50 µL, 0.2 M). The sample and blank absorbances were read at 400 nm. The absorbance of the blank was subtracted from that of the sample and the α-glucosidase inhibitory activity was expressed as acarbose equivalents (mmol ACE/g extract).

Porcine pancreatic lipase (type-II) activity was performed using p-nitrophenyl butyrate (p-NPB) as substrate [3]. Enzyme solution (1 mg/mL) was prepared in 50 mM Tris-HCl (pH 8.0). Fraction solution (25 µL) was mixed with a lipase solution (50 µL) in a 96-well microplate and incubated for 20 min at 25 °C. The reaction was initiated with the addition of p-NPB (5 mM, 50 µL). Similarly, a blank sample (prepared in the same manner but without the extract) was prepared for each of the samples and analysed according to this procedure. Milligrams of orlistat equivalents per gram of dry extract (OEs/g extract) were the measurement unit.

Anti-collagenase enzyme assay was performed to determine the inhibitory effect of *Erica bruckenthalia* plant on collagen activity and the results were calculated as IC50 value. Methanol - aqueous extract obtained from *Erica bruckenthalia* plant was prepared at different concentrations (62.5-1000 µg/mL) and incubated with collagenase enzyme (EC.3.4.23.3) and 50ug/ml concentration FALGPA (Cat No: F5135) used as the substrate of the enzyme in Tris-HCl buffer (50 mM, pH 7.4). 62.5-1000 µg/mL EGCG was used as positive control and a mixture not containing the plant extract was used as negative control. After 1 hour incubation at 37°C, it was measured spectrophotometrically at 340 nm for the determination of anti-collagenase activity. IC50 value was calculated using the dose-response curve from the % inhibition graph. All measurements were repeated three times [4, 5].

Anti-elastase test was applied to determine the elastase enzyme inhibition potential of methanol-aqueous extract obtained from *Erica bruckenthalia* plant [4, 5]. Porcine pancreatic elastase (EC: 3.4.21.36) was supplied by Sigma-Aldrich as the elastase enzyme source and was prepared in 0.1 M Tris-HCl buffer (pH 8.0) at 37 °C. 1.6 mM of AAAPVN (Cat No: S4760) was used as the substrate. In the study, plant samples were prepared at increasing concentrations (62.5-1000 µg/mL) and mixed with enzyme and substrate. The extract and enzyme-substrate mixture were measured spectrophotometrically at 410 nm wavelength 20 min after mixing. While a solution containing only enzyme and substrate was used as a negative control,

EGCG, known to inhibit elastase, was used at increasing concentrations as a positive control (62.5-1000 µg/mL). IC50 was calculated according to the dose-response curve obtained after the measurement [5].

Anti-hyaluronidase test was applied to determine the potential of methanol-aqueous extract obtained from *Erica bruckenthalia* plant to inhibit hyaluronidase enzyme (EC.3.2.1.35). After dissolving hyaluronidase enzyme in 0.1 M acetate buffer (pH 4.5), hyaluronidase enzyme was incubated with increasing concentrations (62.5-1000 µg/mL) of *Erica bruckenthalia* plant and tannic acid used as positive control for 20 minutes at 37°C. After the incubation period, 0.03% (w/v) hyaluronic acid substrate (Cat No: 924474) was added to the mixture and incubated again at 37°C for 20 minutes and then spectrophotometric measurement was made at 600 nm. IC50 was calculated according to the dose-response curve obtained after the measurement [5, 6].

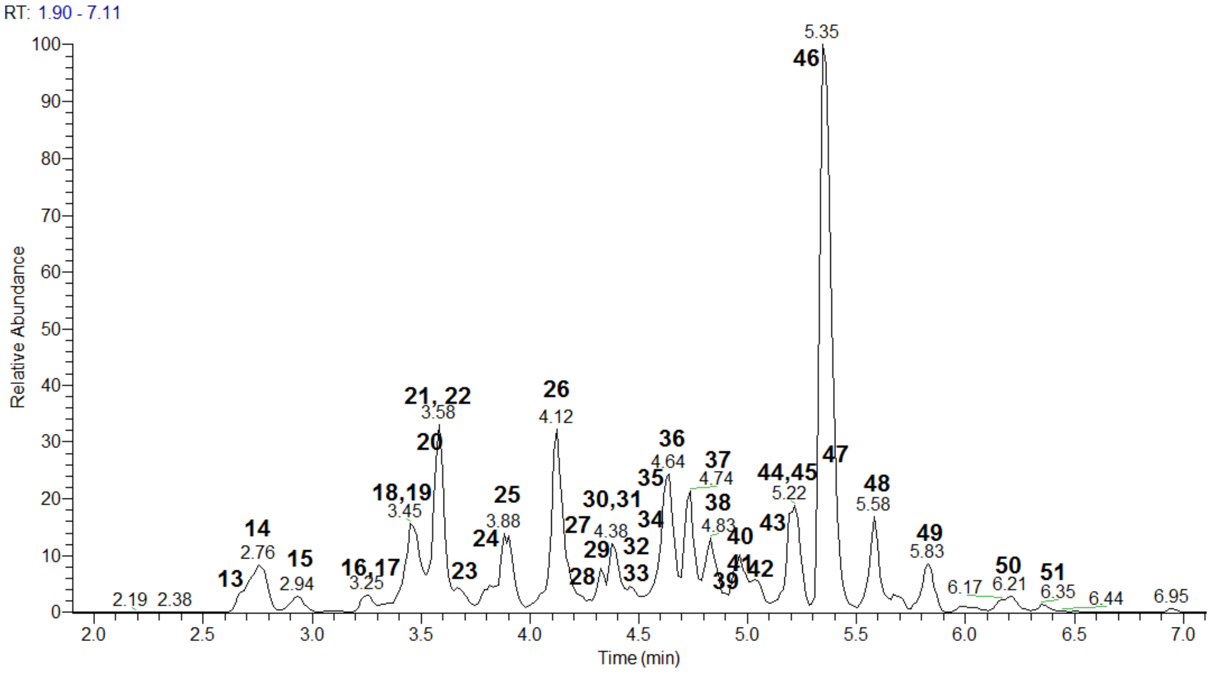

Figure S1. Extracted ion chromatogram of proanthocyanidins (PACs) (for numbers and fragmentation patterns, see Table S1).

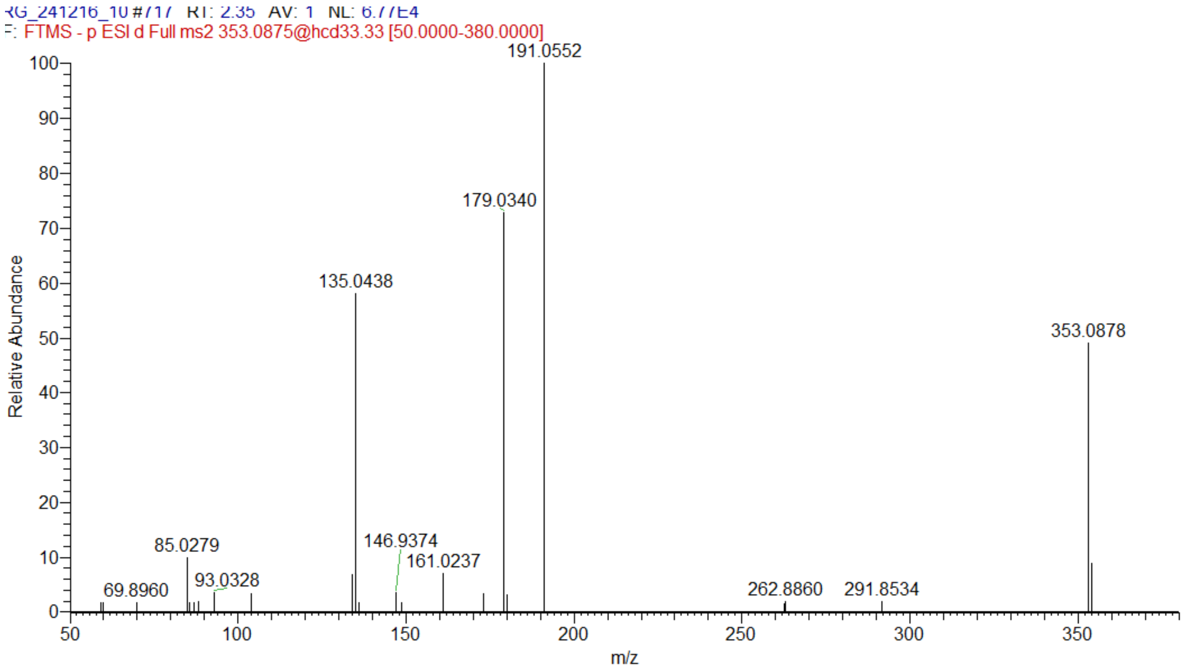

Figure S2. (-) ESI-MS/MS spectrum of neochlorogenic acid (**1**) at *m*/*z* 353.0878 (353.0860-353.0896) (mass tolerance 5 ppm) (for numbers and fragmentation patterns, see Table S1).

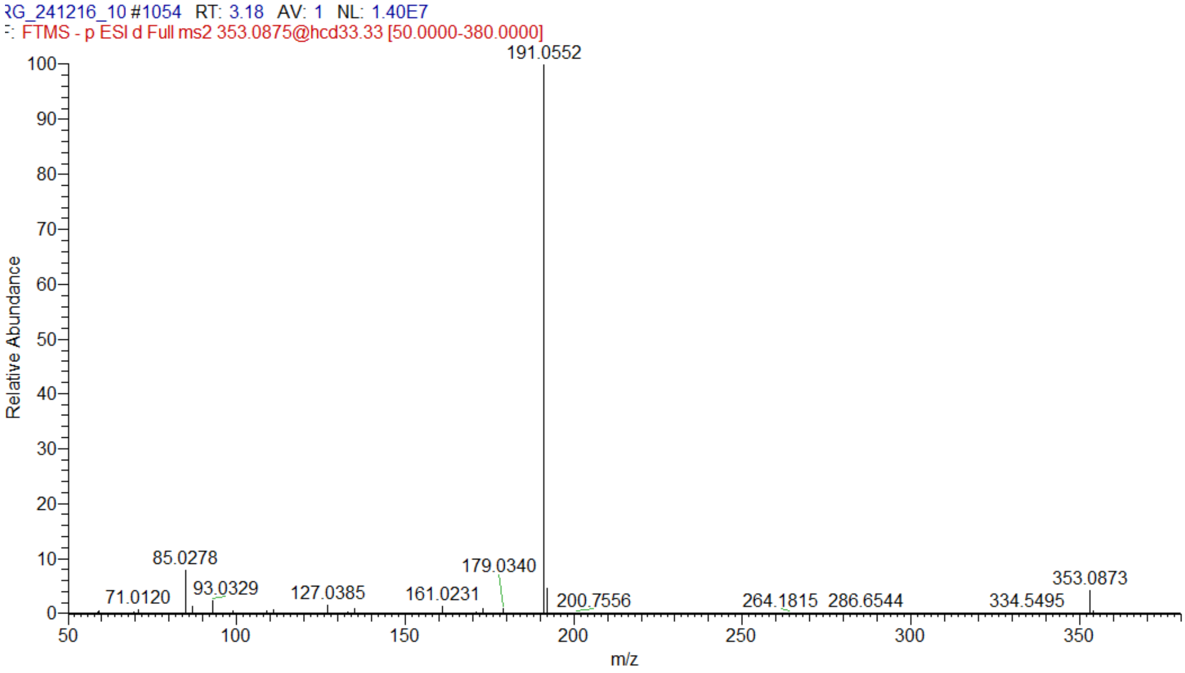

Figure S3. (-) ESI-MS/MS spectrum of chlorogenic acid (**2**) at *m*/*z* 353.0878 (353.0860-353.0896) (mass tolerance 5 ppm) (for numbers and fragmentation patterns, see Table S1).

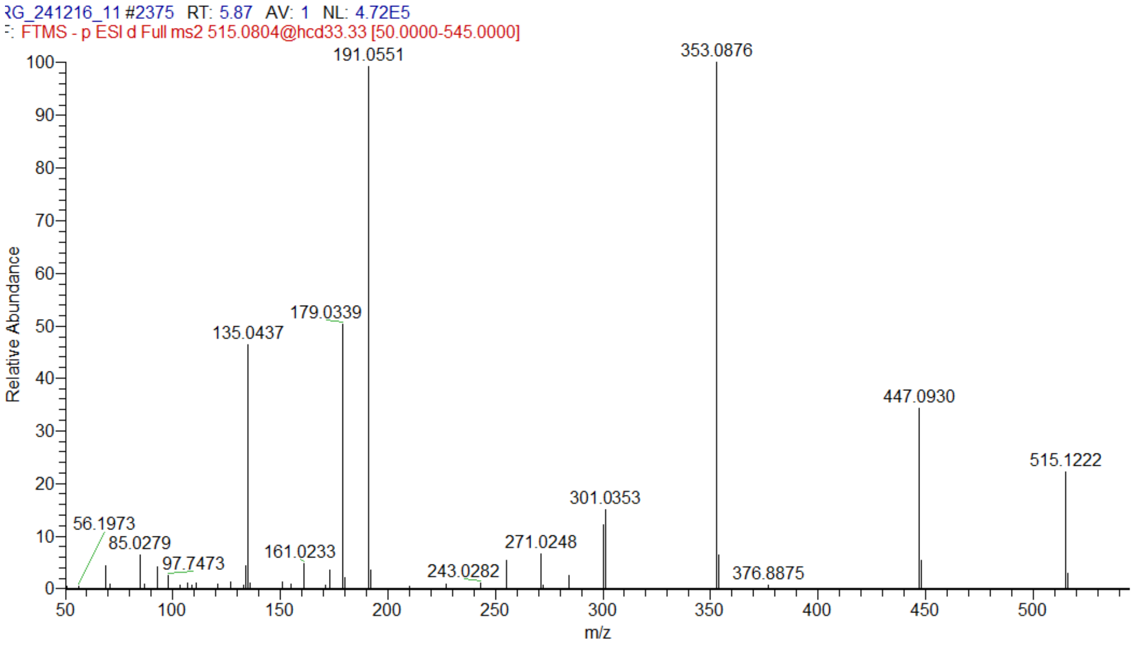

Figure S4. (-) ESI-MS/MS spectrum of 3, 5-dicaffeoylquinic acid (**11**) at *m/z* 515.1195 (515.1195-515.1221) (mass tolerance 5 ppm) (for numbers and fragmentation patterns, see Table S1).

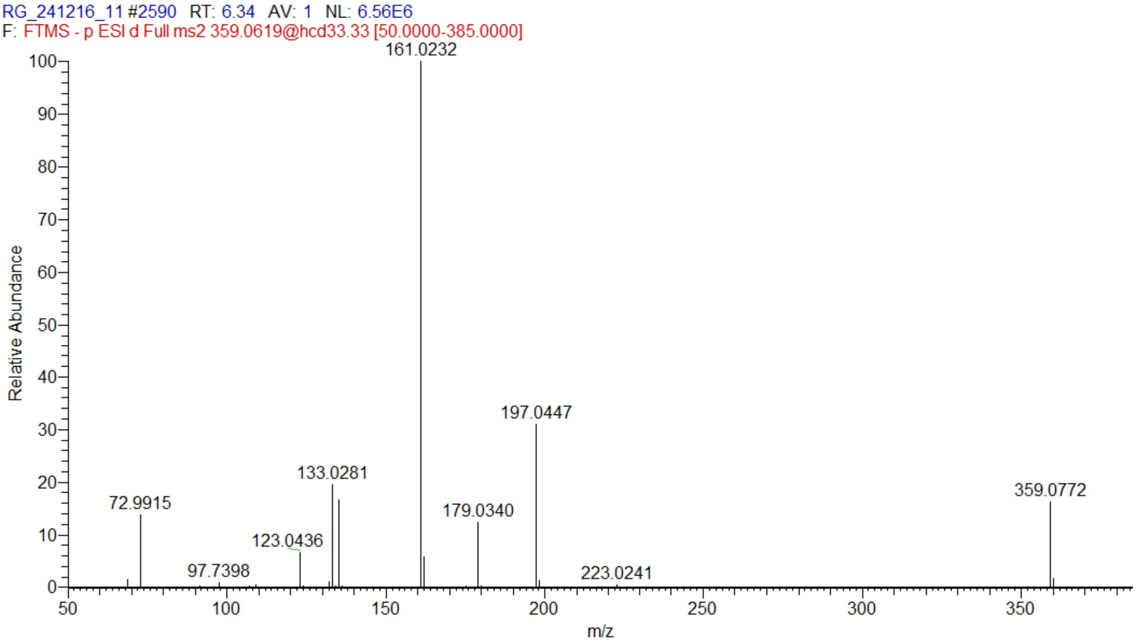

Figure S5. (-) ESI-MS/MS spectrum of rosmarinic acid (**12**) at *m/z* 359.0772 (359.0754-359.0790) (mass tolerance 5 ppm) (for numbers and fragmentation patterns, see Table S1).

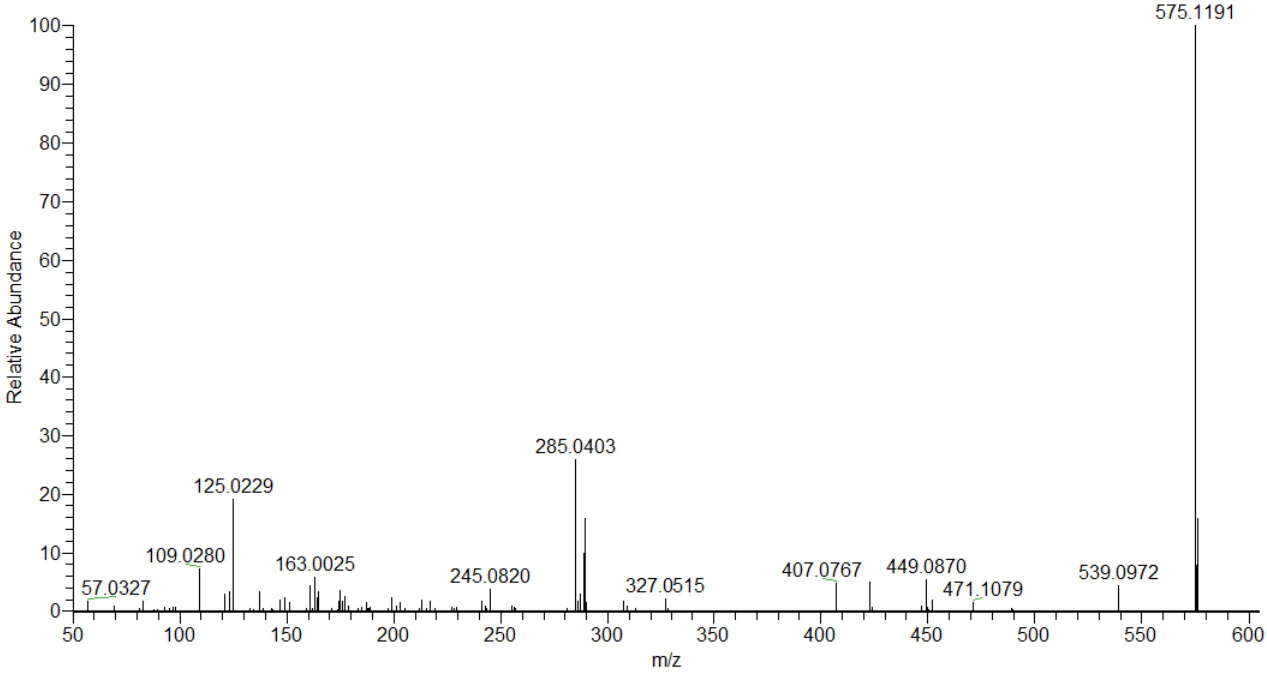

Figure S6. (-) ESI-MS/MS spectrum of proanthocyanidin dimer A-type (**37**) (for numbers and fragmentation patterns, see Table S1).

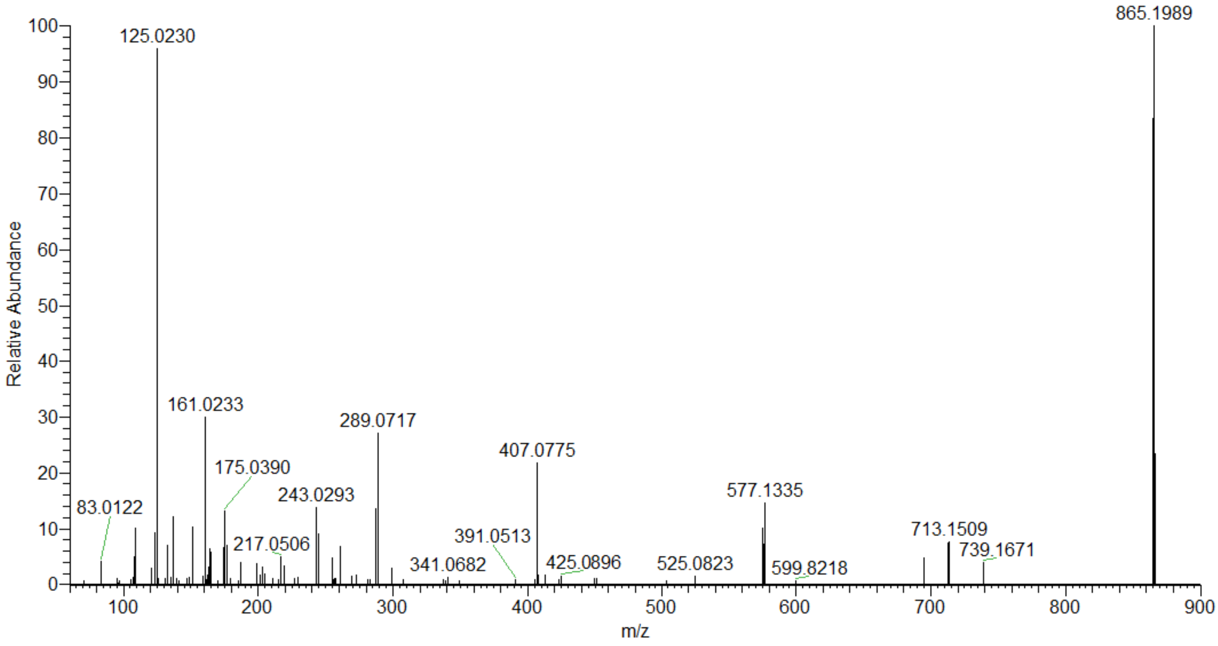

Figure S7. (-) ESI-MS/MS spectrum of proanthocyanidin trimer B-type (**17**) (for numbers and fragmentation patterns, see Table S1).

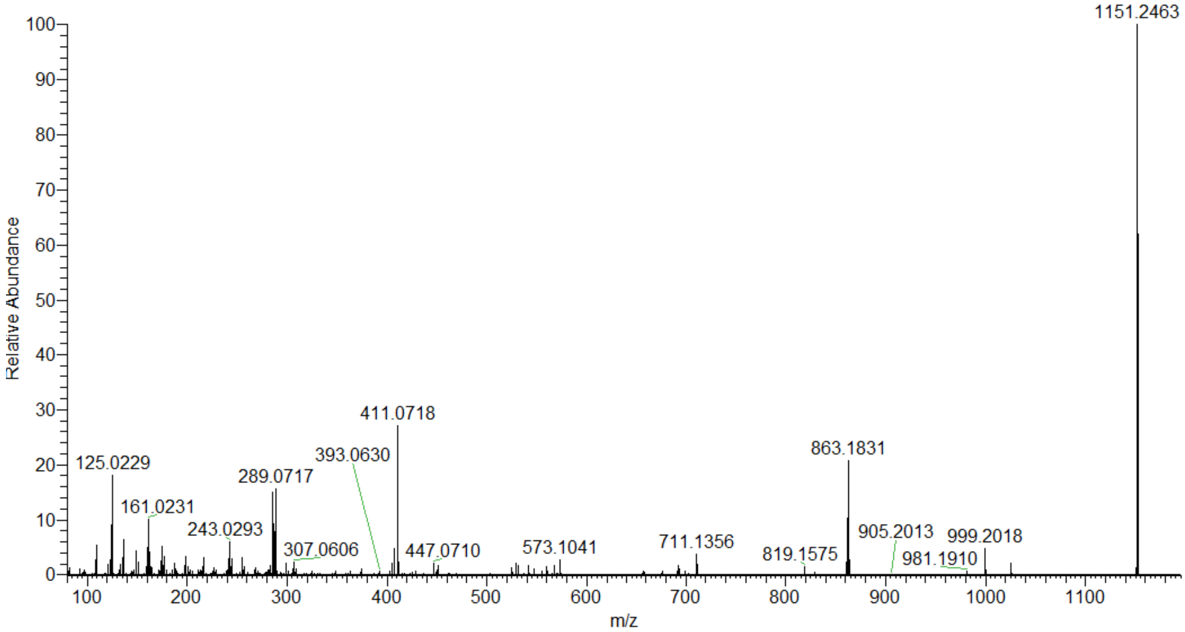

Figure S8. (-) ESI-MS/MS spectrum of proanthocyanidin tetramer A, B-type (**20**) (for numbers and fragmentation patterns, see Table S1).

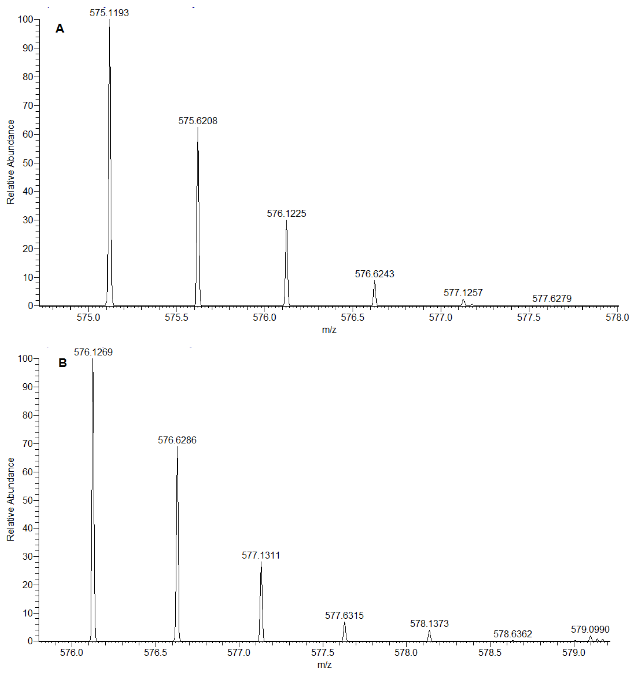

Figure S9. Monoisotopic peak patterns showing the doubly-charged ions of proanthocyanidin tetramers at *m/z* 575.119 (A) and at *m/z* 576.126 (B).

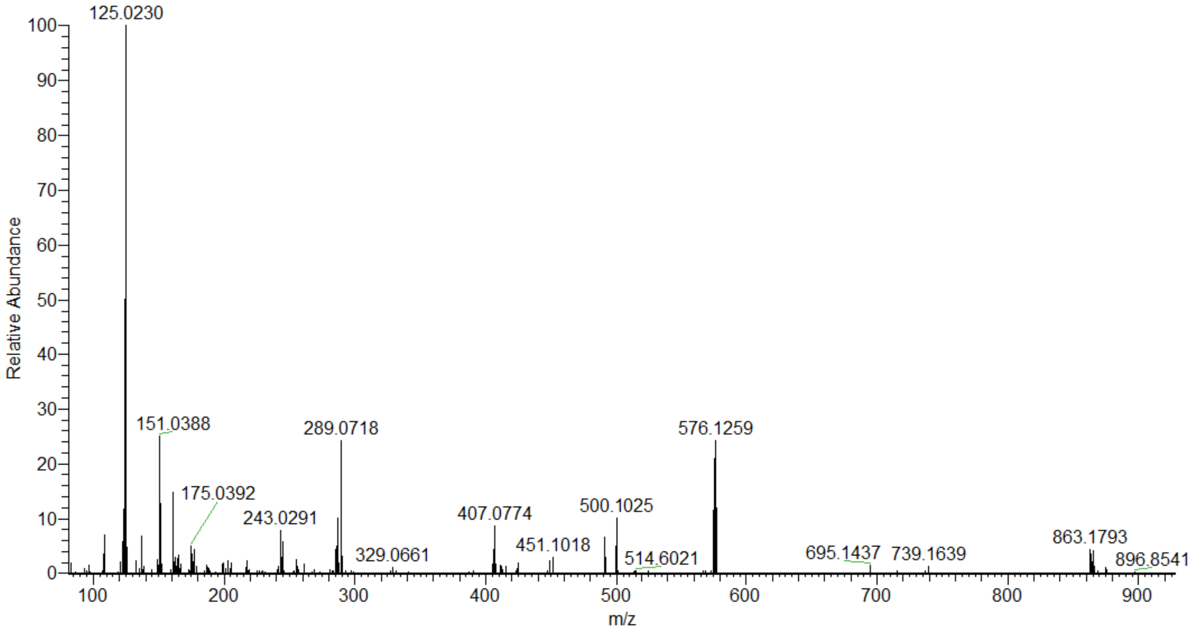

Figure S10. (-) ESI-MS/MS spectrum of proanthocyanidin tetramer B-type (**35**) (for numbers and fragmentation patterns, see Table S1).

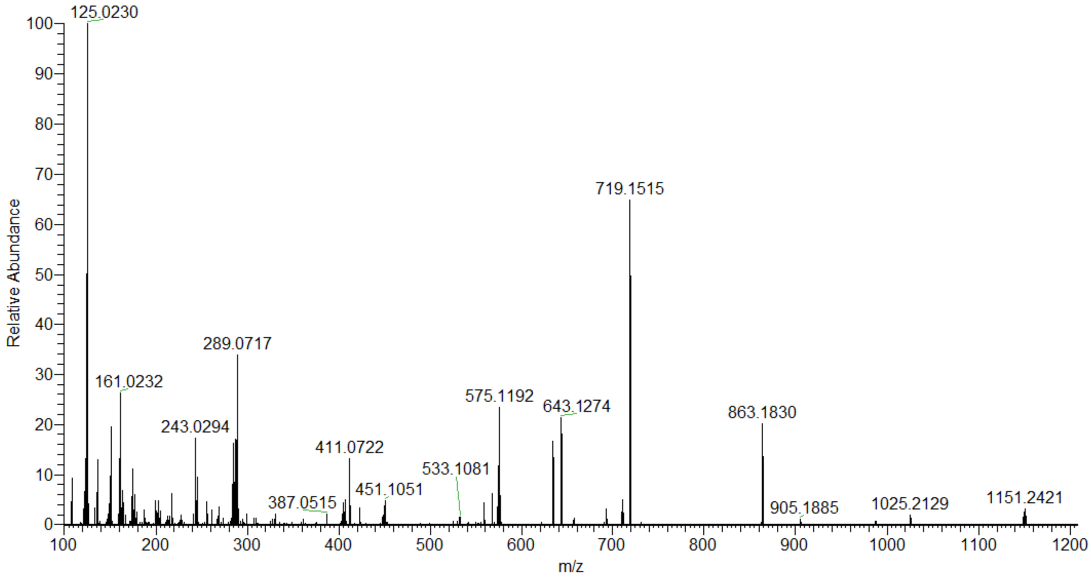

Figure S11. (-) ESI-MS/MS spectrum of proanthocyanidin pentamer A, B-type (**19**) (for numbers and fragmentation patterns, see Table S1).

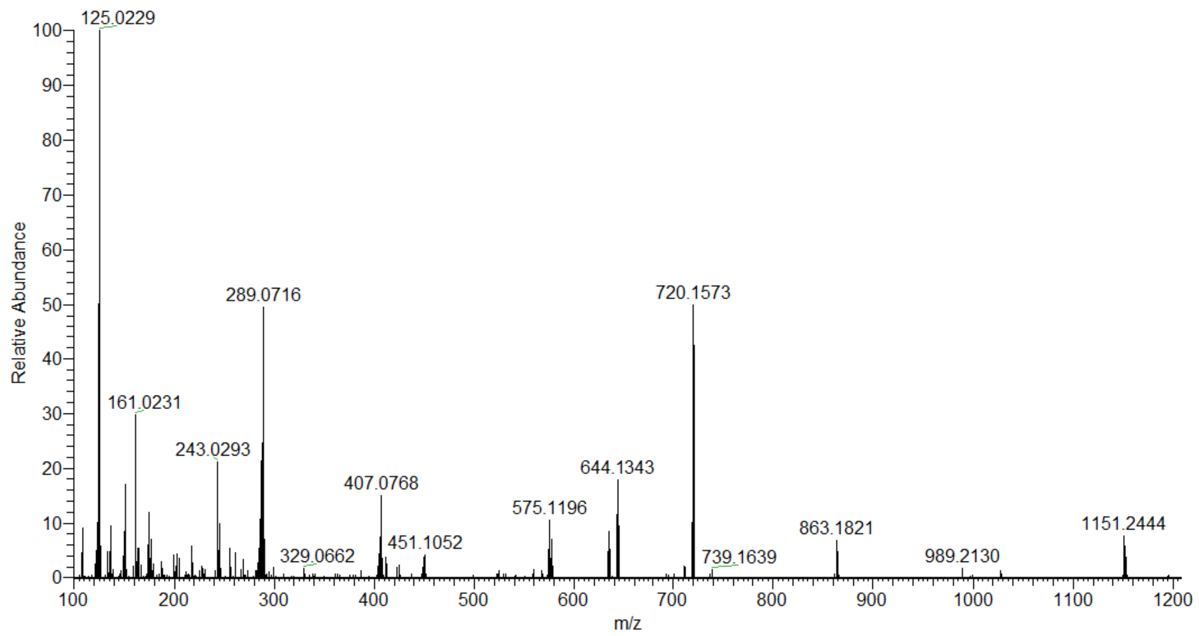

Figure S12. (-) ESI-MS/MS spectrum of proanthocyanidin pentamer B-type (**32**) (for numbers and fragmentation patterns, see Table S1).

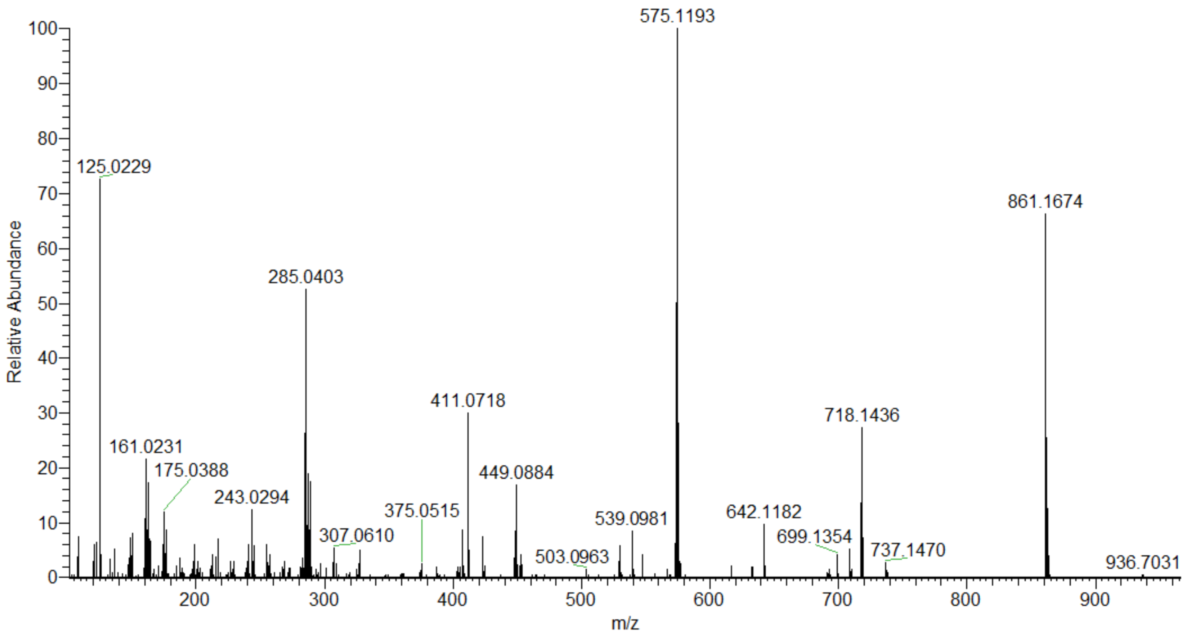

Figure S13. (-) ESI-MS/MS spectrum of proanthocyanidin pentamer A-type (**45**) (for numbers and fragmentation patterns, see Table S1).

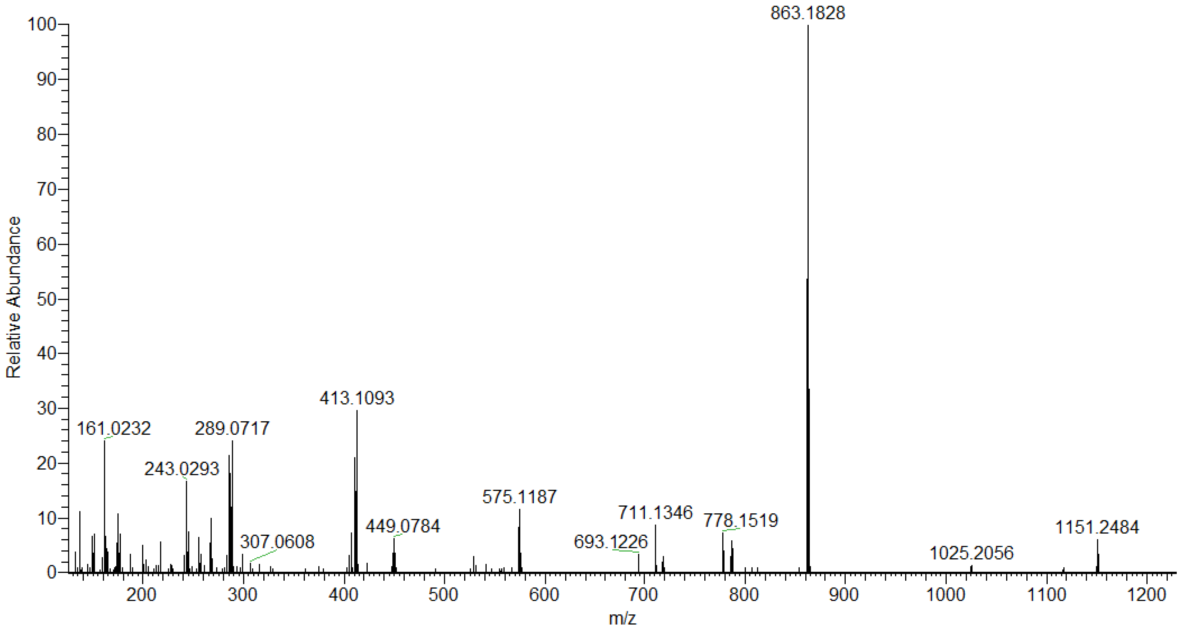

Figure S14. (-) ESI-MS/MS spectrum of proanthocyanidin hexamer A, B-type (**28**) (for numbers and fragmentation patterns, see Table S1).

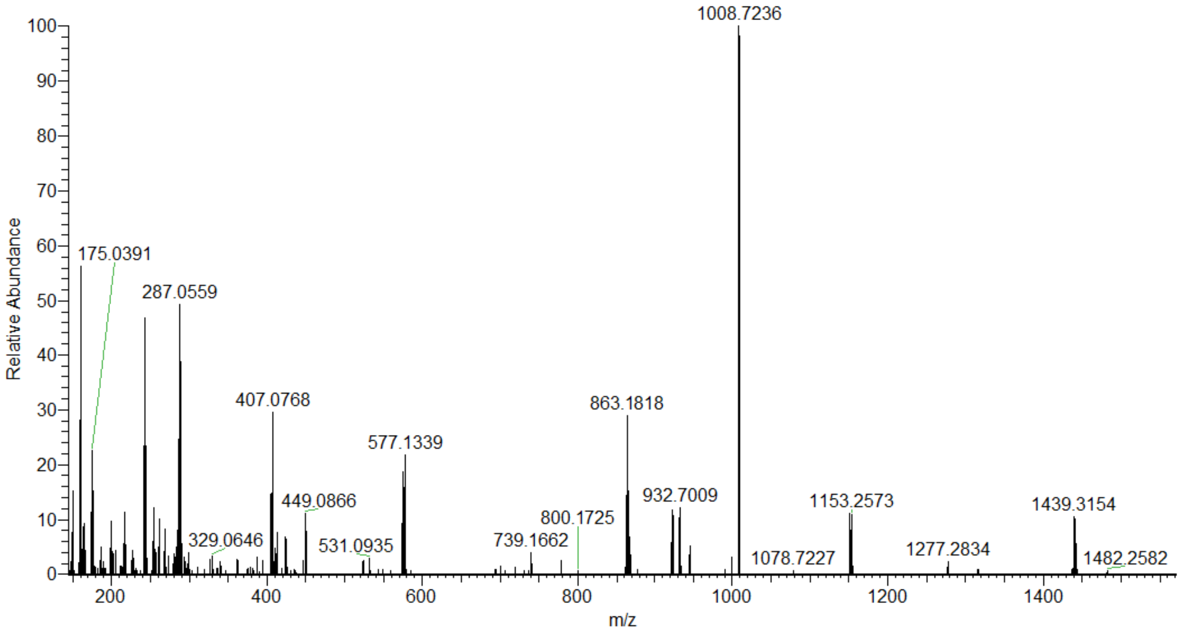

Figure S15. (-) ESI-MS/MS spectrum of proanthocyanidin heptamer A, B-type (**39**) (for numbers and fragmentation patterns, see Table S1).

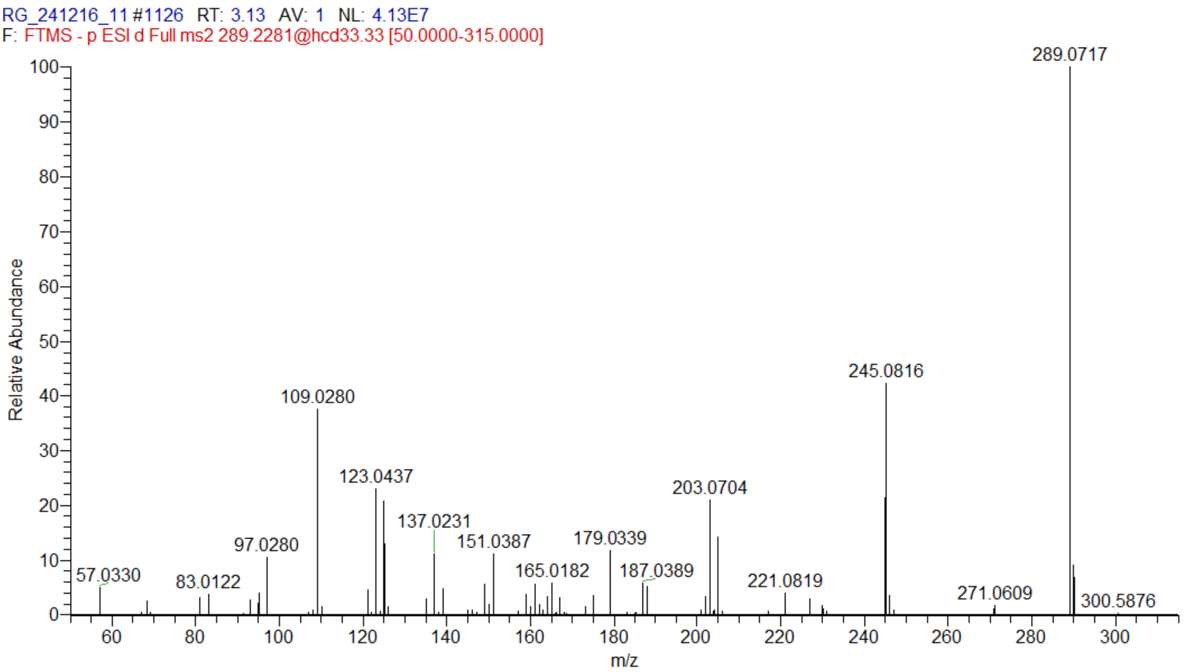

Figure S16. (-) ESI-MS/MS spectrum of (+) catechin (**53)** at *m*/*z* 289.0718 (289.0704-287.0732) (mass tolerance 5 ppm) (for numbers and fragmentation patterns, see Table S1).

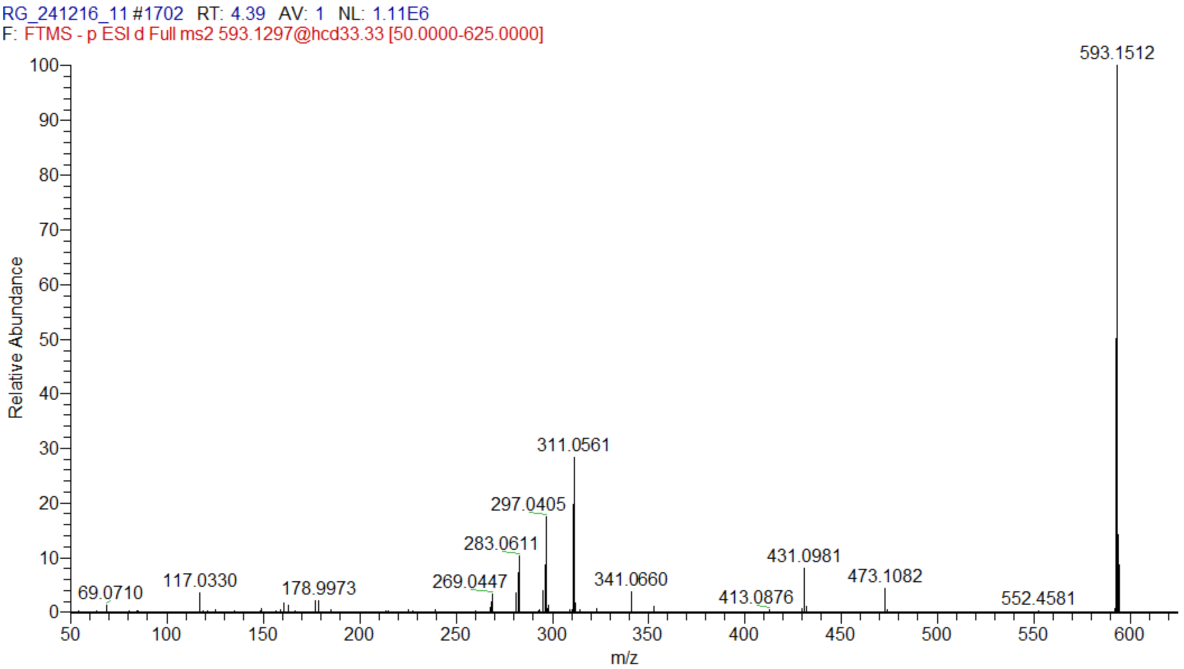

Figure S17. (-) ESI-MS/MS spectrum of saponarin (**53)** at *m*/*z* 593.1512 (593.1482-593.1542) (mass tolerance 5 ppm) (for numbers and fragmentation patterns, see Table S1).

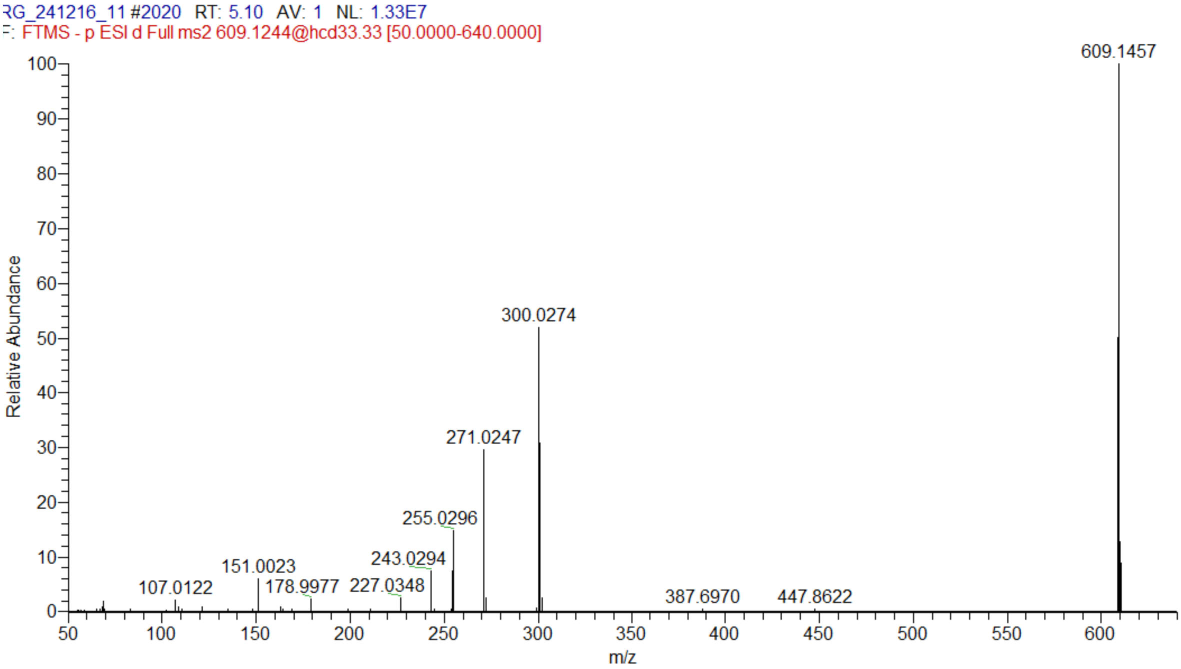

Figure S18. (-) ESI-MS/MS spectrum of rutin (**72)** at *m/z* 609.1464 (609.1434-609.1494) (mass tolerance 5 ppm) (for numbers and fragmentation patterns, see Table S1).

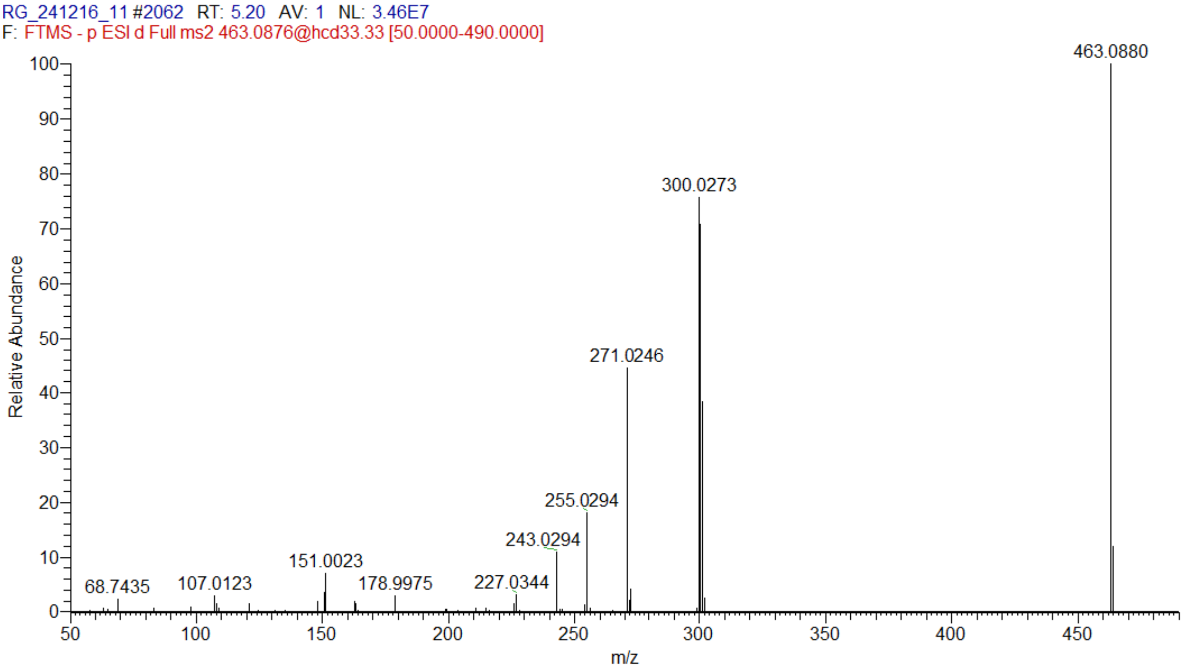

Figure S19. (-) ESI-MS/MS spectrum of isoquercitrin (**74)** at *m/z* 463.0886 (463.0886-463.0909) (mass tolerance 5 ppm) (for numbers and fragmentation patterns, see Table S1).

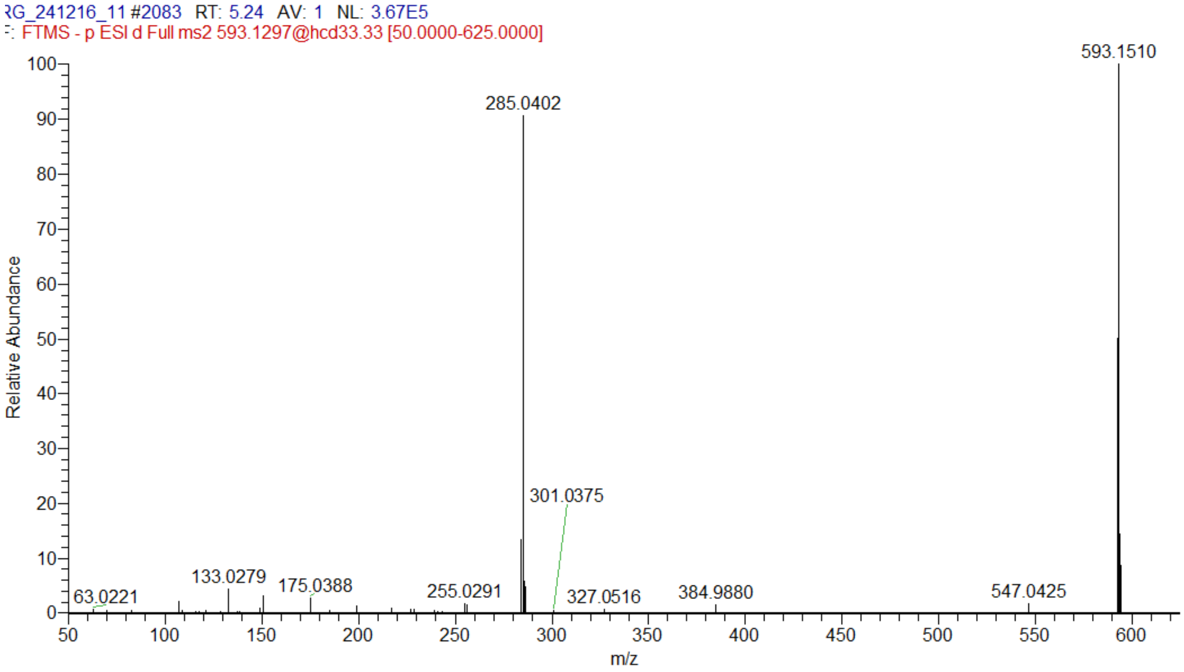

Figure S20. (-) ESI-MS/MS spectrum of saponarin (**76**) at *m/z* 593.1512 (593.1482-593.1542) (mass tolerance 5 ppm) (for numbers and fragmentation patterns, see Table S1).

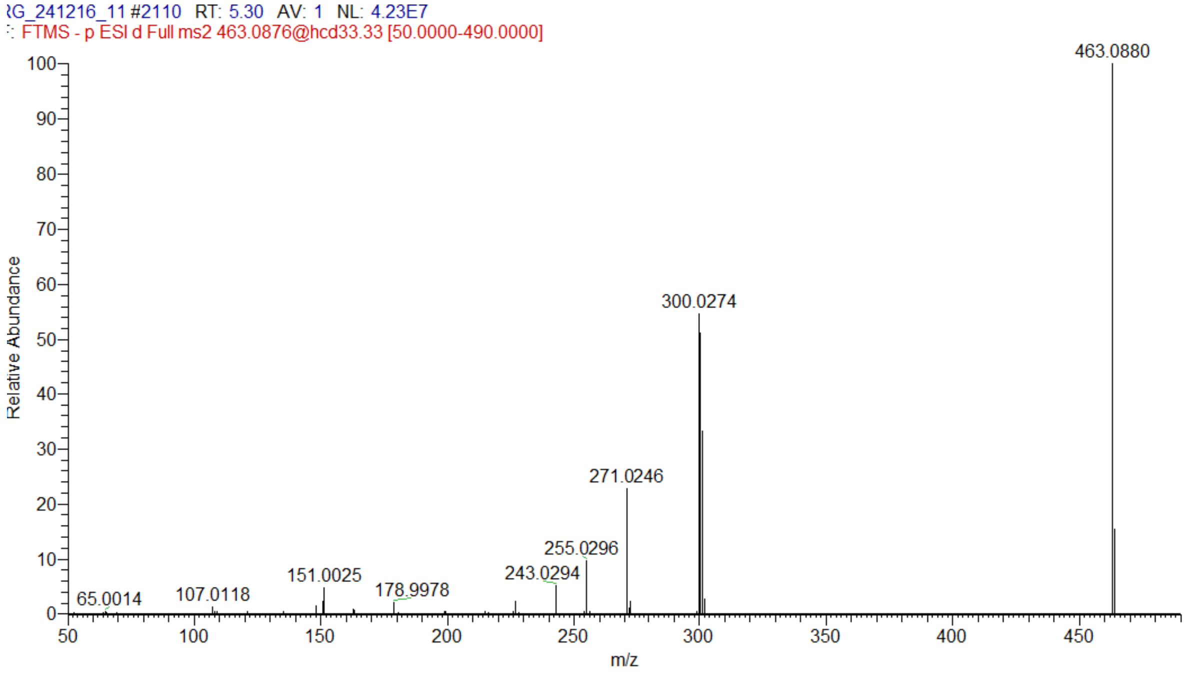

Figure S21. (-) ESI-MS/MS spectrum of hyperoside (**77**) at *m/z* 463.0886 (463.0886-463.0909) (mass tolerance 5 ppm) (for numbers and fragmentation patterns, see Table S1).

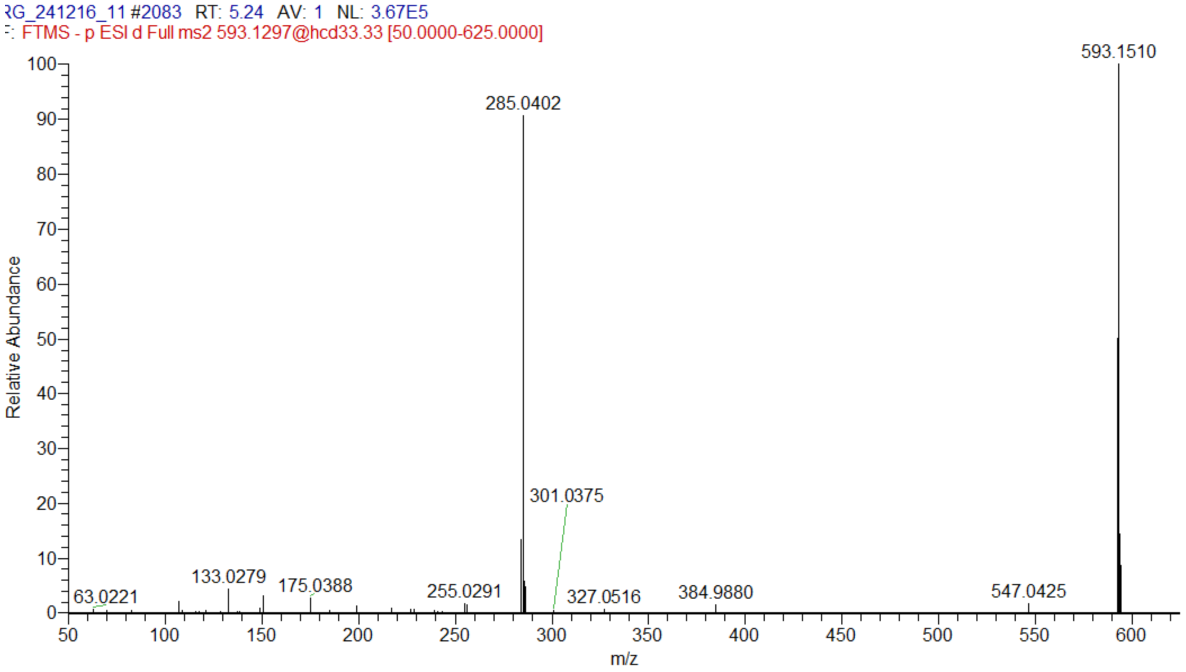

Figure S22. (-) ESI-MS/MS spectrum of luteolin 7-*O*-glucoside (**79**) at m/z 447.0933 (447.0911-447.0955) (mass tolerance 5 ppm) (for numbers and fragmentation patterns, see Table S1).

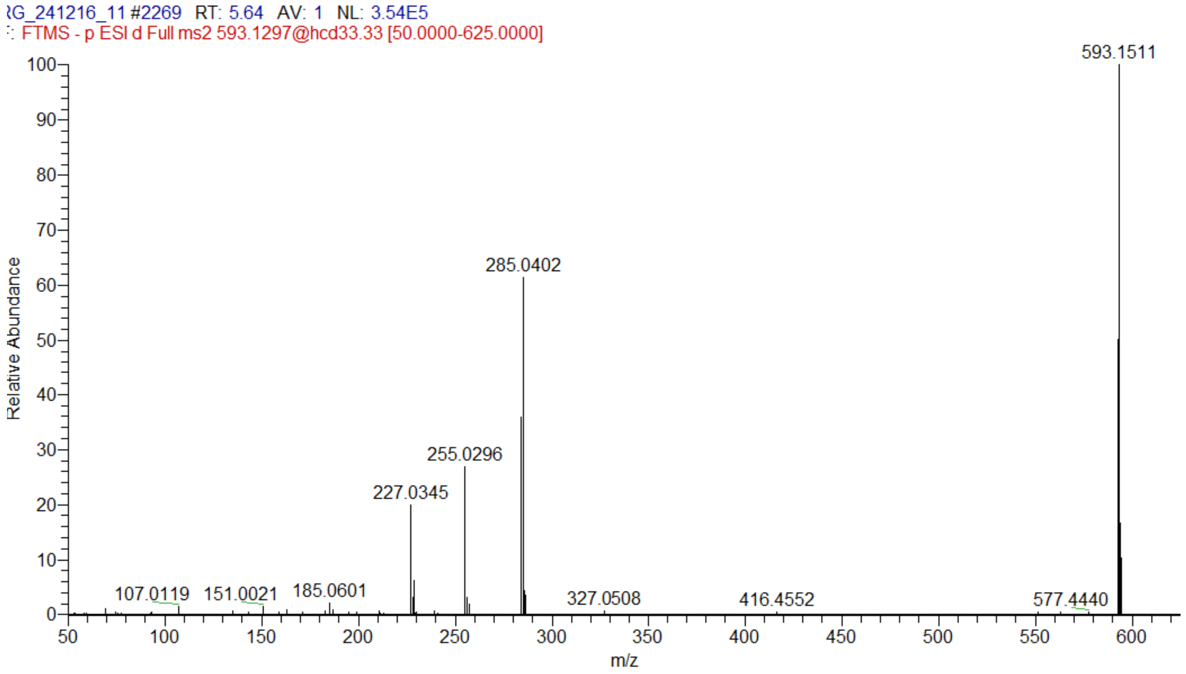

Figure S23. (-) ESI-MS/MS spectrum of kaempferol 3-*O*-rutinoside (**83)** at *m/z* 593.1512 (593.1482-593.1542) (mass tolerance 5 ppm) (for numbers and fragmentation patterns, see Table S1).

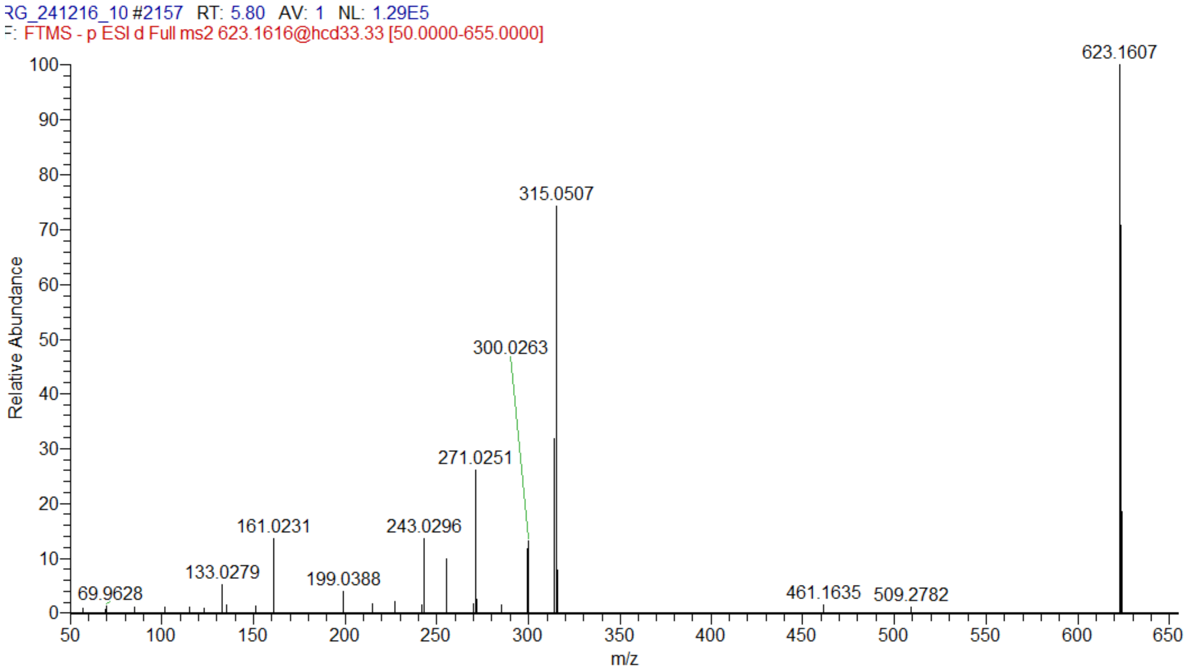

Figure S24. (-) ESI-MS/MS spectrum of isorhamnetin 3-*O*-rutinoside (**85)** at *m/z* 623.1618 (623.1587-623.1649) (mass tolerance 5 ppm) (for numbers and fragmentation patterns, see Table S1).

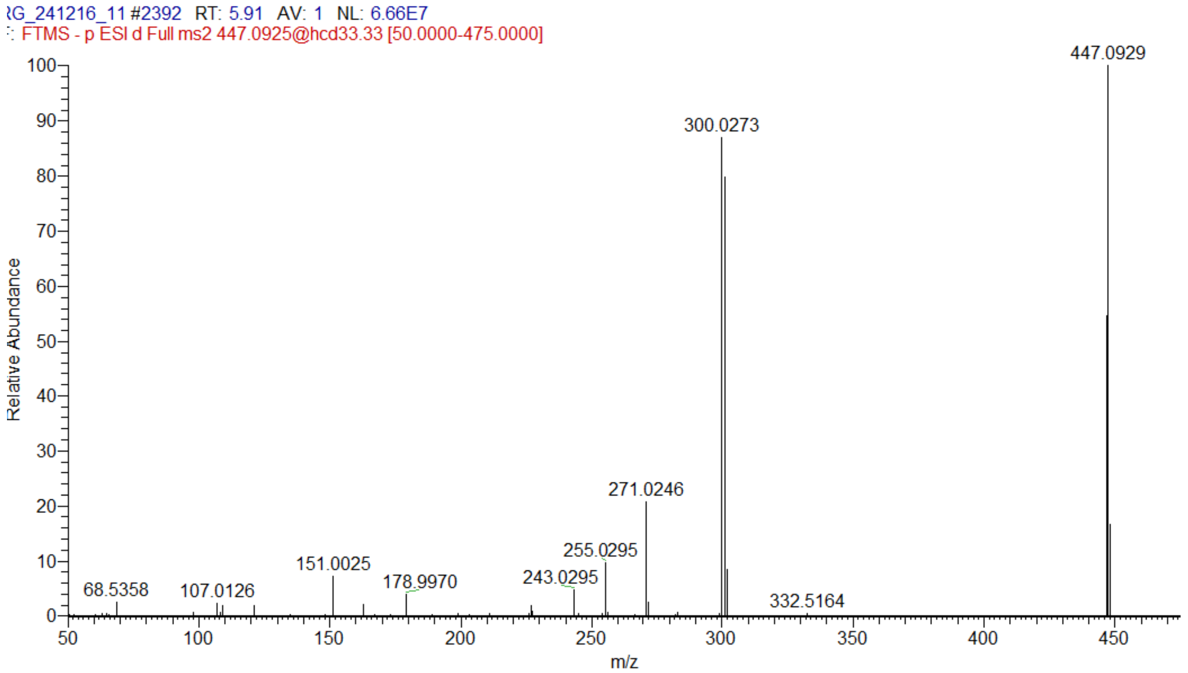

Figure S25. (-) ESI-MS/MS spectrum of quercitrin (**86)** at *m/z* 447.0933 (447.0911-0955) (mass tolerance 5 ppm) (for numbers and fragmentation patterns, see Table S1).

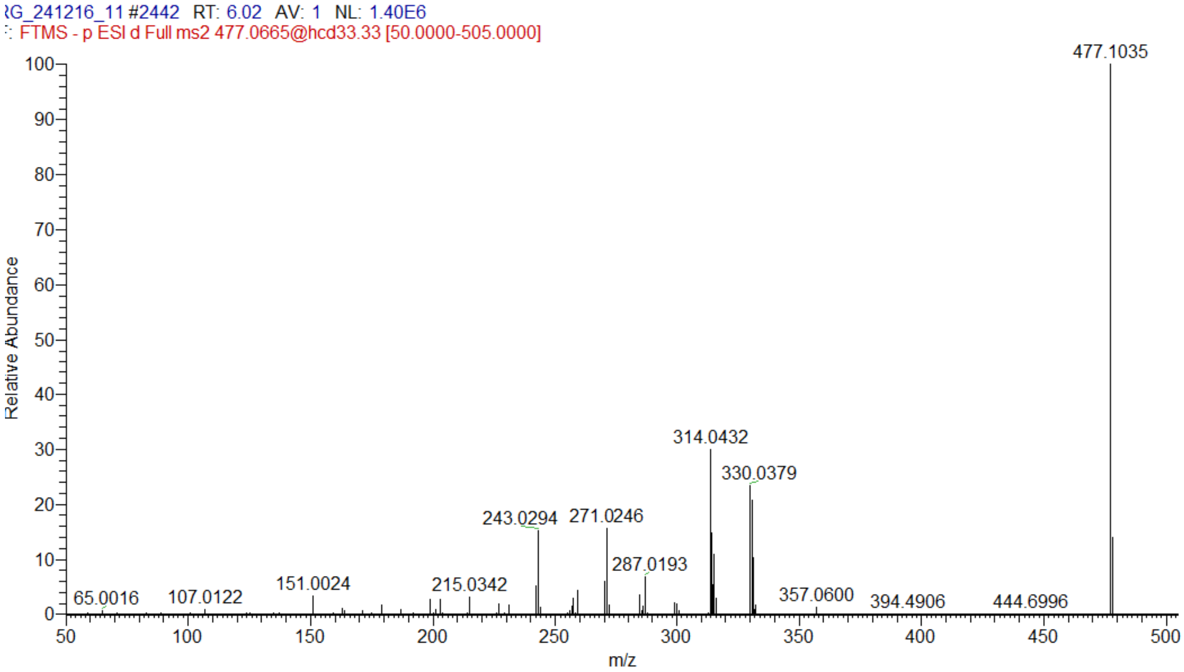

Figure S26. (-) ESI-MS/MS spectrum of isorhamnetin 3-*O*-glucoside (**87)** at *m*/*z* 477.1044 (477.1020-477.1068) (mass tolerance 5 ppm) (for numbers and fragmentation patterns, see Table S1).

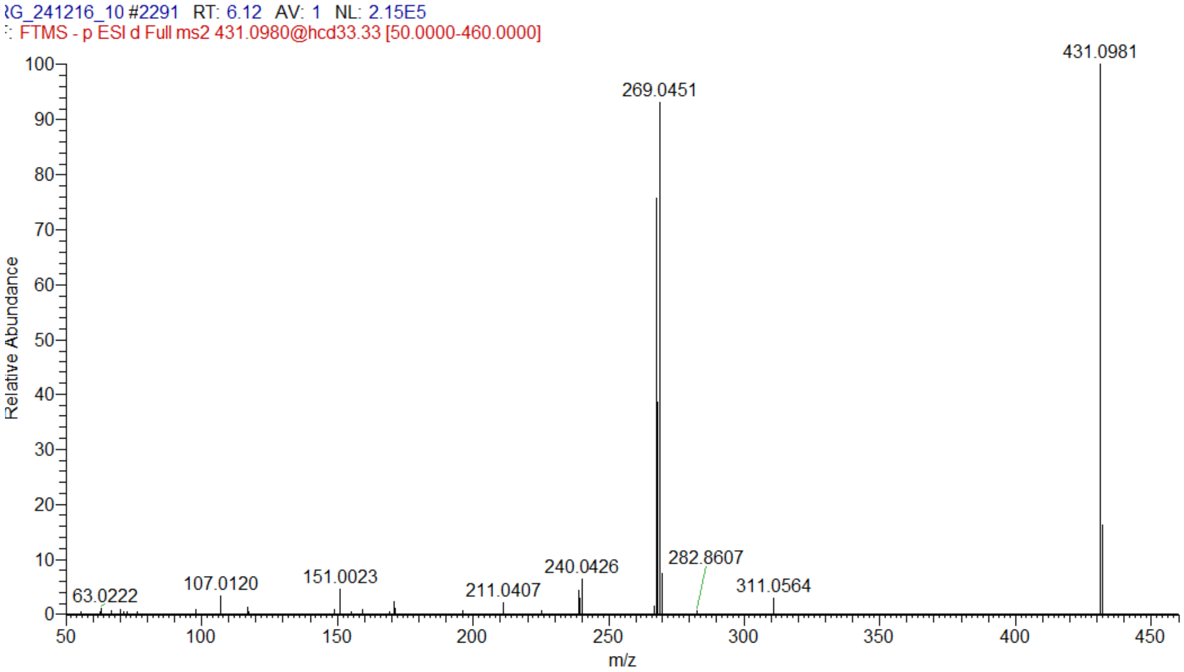

Figure S27. (-) ESI-MS/MS spectrum of apigenin 7-*O*-glucoside (**89**) at *m*/*z* 431.0983 (431.0961-431.1005) (mass tolerance 5 ppm) (for numbers and fragmentation patterns, see Table S1).

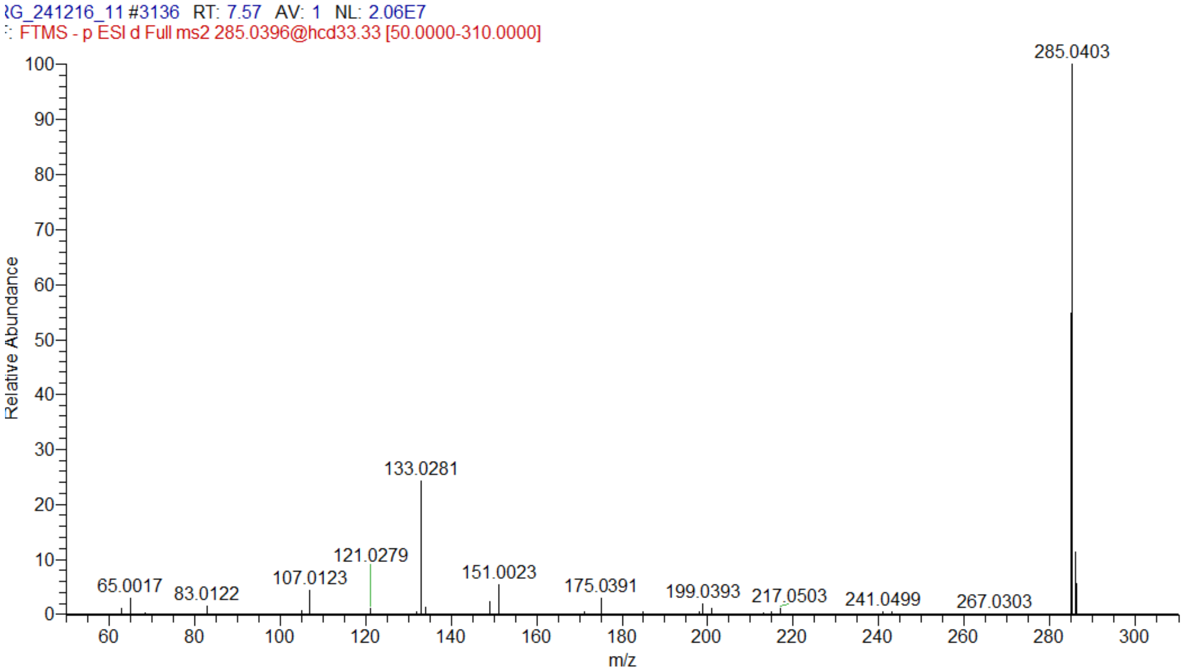

Figure S28. (-) ESI-MS/MS spectrum of luteolin (**98)** at *m/z* 285.0405 (285.0391-285.0419) (mass tolerance 5 ppm) (for numbers and fragmentation patterns, see Table S1).

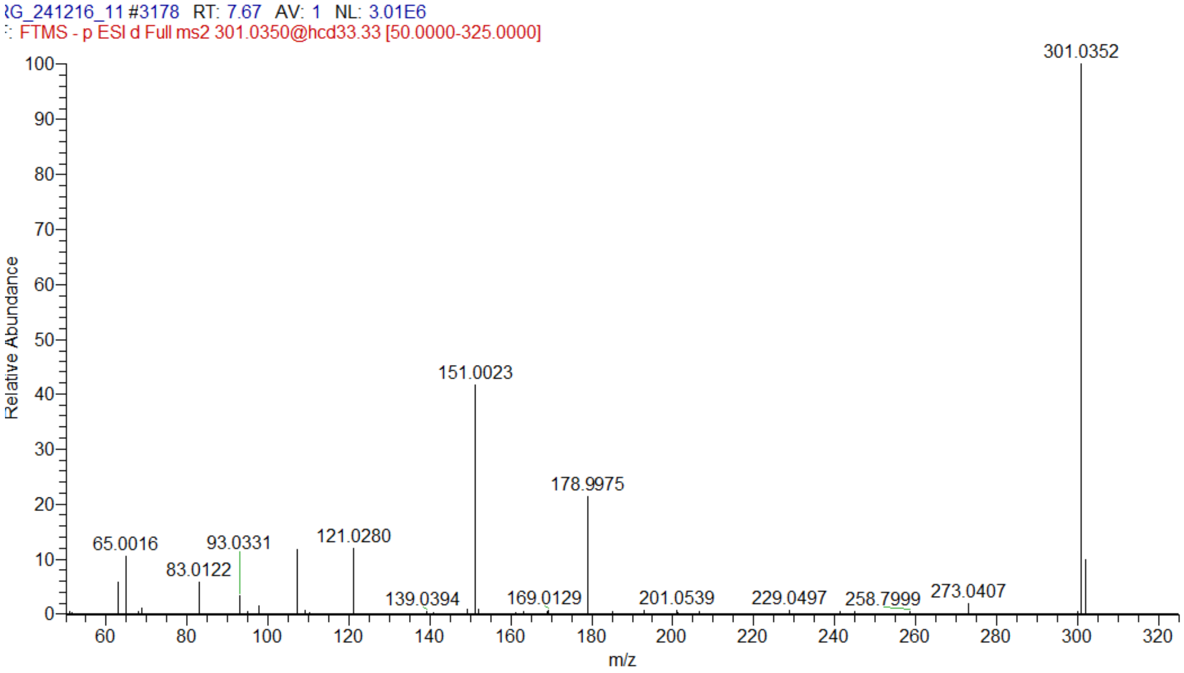

Figure S29. (-) ESI-MS/MS spectrum of quercetin (**99)** at *m/z* 301.0354 (301.0339-301.0369) (mass tolerance 5 ppm) (for numbers and fragmentation patterns, see Table S1).

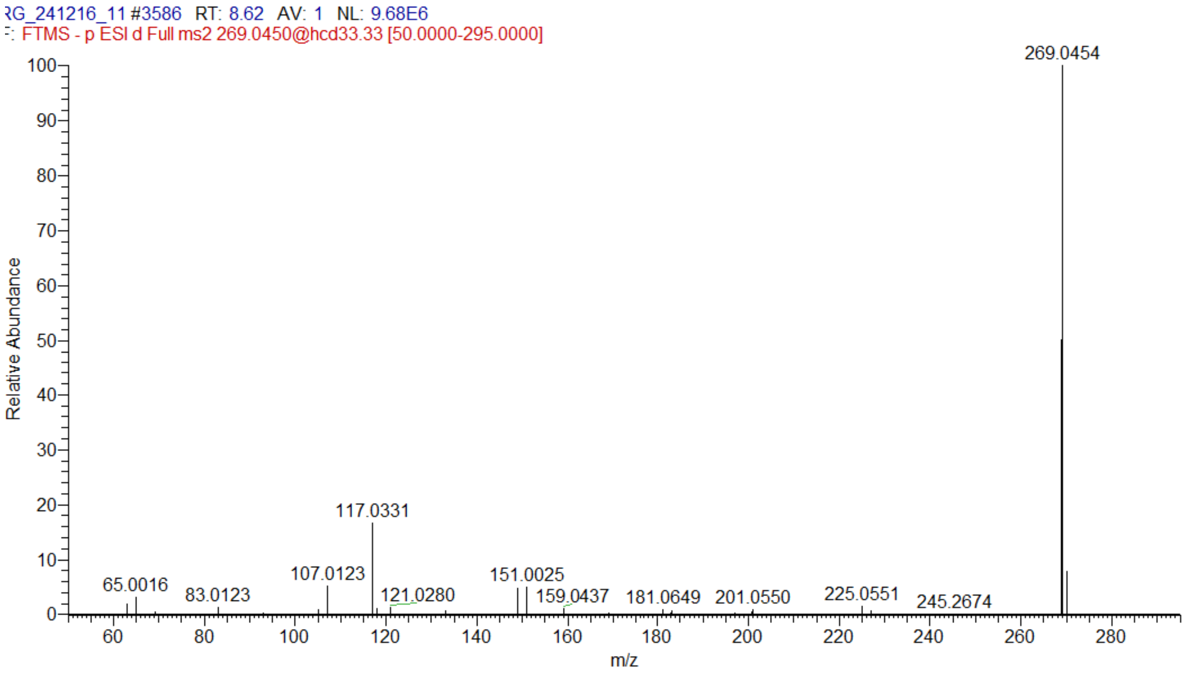

Figure S30. (-) ESI-MS/MS spectrum of apigenin (**100)** at *m*/*z* 269.0457 (269.0440-269.0470) (mass tolerance 5 ppm) (for numbers and fragmentation patterns, see Table S1).

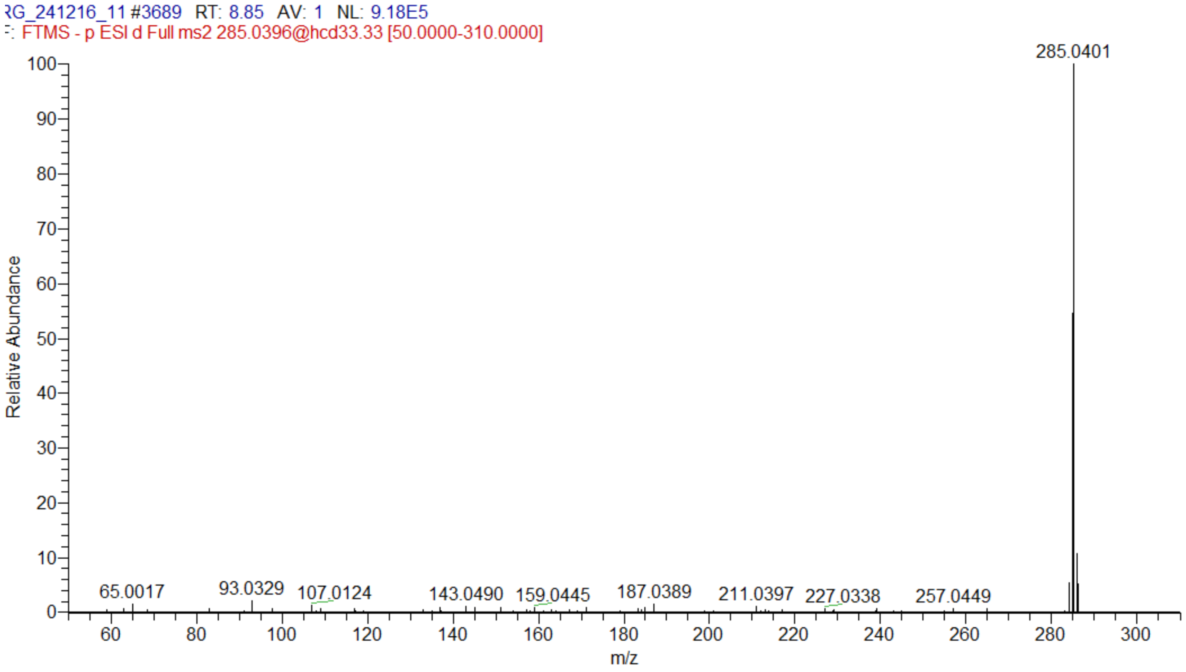

Figure S31. (-) ESI-MS/MS spectrum of kaempferol (**101)** at *m*/*z* 285.0405 (285.0391-285.0419) (mass tolerance 5 ppm) (for numbers and fragmentation patterns, see Table S1).

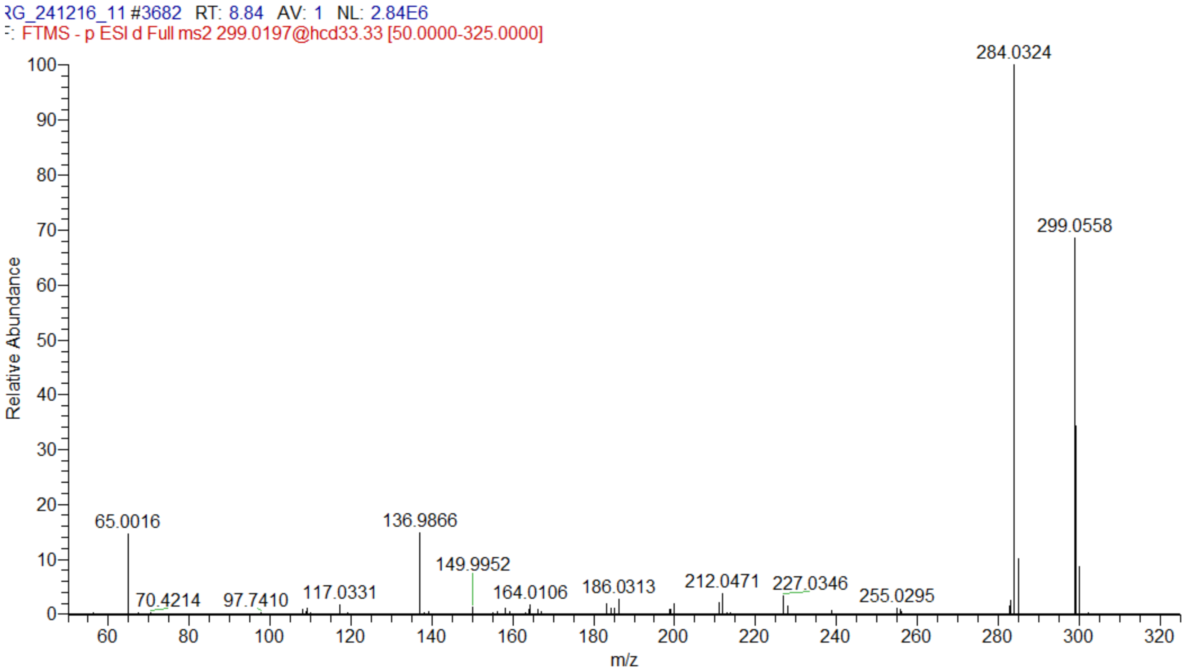

Figure S32. (-) ESI-MS/MS spectrum of hispidulin (**102**) at *m/z* 299.0563 (299.0548-299.0578) (mass tolerance 5 ppm) (for numbers and fragmentation patterns, see Table S1).

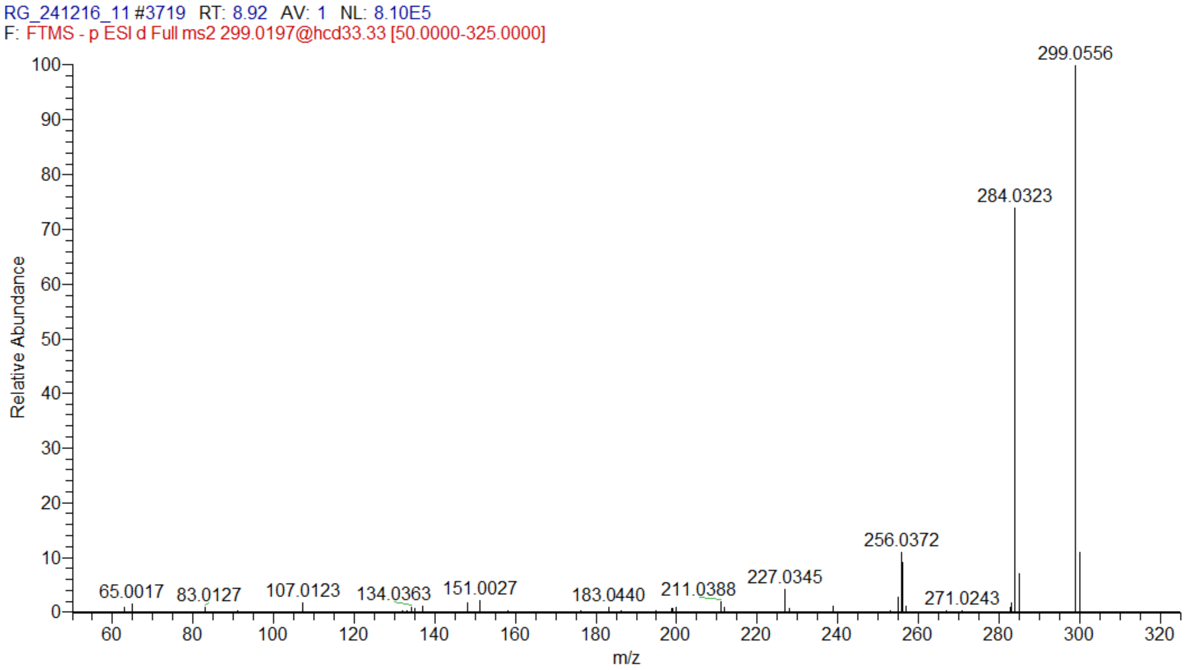

Figure S33. (-) ESI-MS/MS spectrum of chrysoeriol (**104**) at *m/z* 299.0563 (299.0548-299.0578) (mass tolerance 5 ppm) (for numbers and fragmentation patterns, see Table S1).

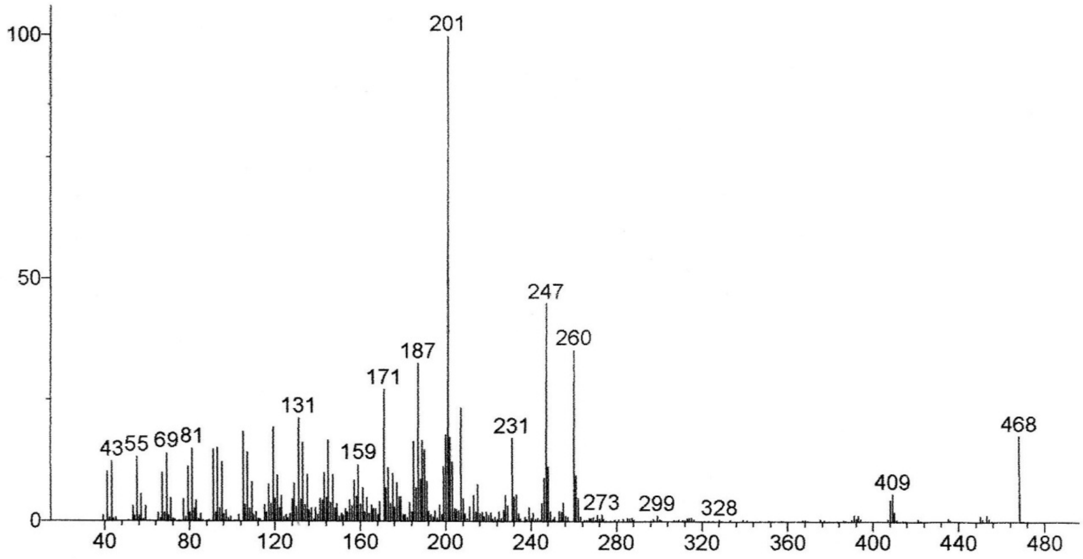

Figure S34. The mass spectrum of the unidentified triterpenoid acid, tentatively identified as micrometric acid methyl ester.

Table S1. Secondary metabolites in *E. spiculifolia* methanol-aqueous extracts.

№	Identified/tentatively annotated compound	Molecular formula	Exact mass [M-H]-	Fragmentation pattern in (-) ESI-MS/MS	t_R (min)	Δ ppm
**Acylquinic acids**						
1.	neochlorogenic acid[a]	$C_{16}H_{18}O_9$	353.0878	353.0878 (50.2), 191.0552 (100), 179.0340(74.8), 161.0237 (6.9), 135.0438 (56.4), 93.0328 (3.6), 85.0279 (10.0)	2.36	0.070
2.	chlorogenic acid[a]	$C_{16}H_{18}O_9$	353.0878	353.0873 (4.3), 191.0552 (100), 173.0444 (0.9), 161.0231 (1.3), 111.0435 (0.7), 93.0329 (2.4), 85.0278 (7.7),	3.18	-1.488
3.	4-caffeoylquinic acid	$C_{16}H_{18}O_9$	353.0878	353.0877 (39.8), 191.0551 (42.3), 179.0339 (67.9), 173.0444 (100), 135.0437 (56.4), 111.0438 (3.4), 93.0330 (24.4), 85.0278 (8.9)	3.35	-0.355
4.	3-feruloylquinic acid	$C_{17}H_{20}O_9$	367.1035	367.1031 (22.1), 193.0497 (100), 191.0550 (2.9), 173.0447 (4.1), 149.0595 (3.7), 135.0393 (2.4), 134.0359 (50.0), 111.0436 (0.6), 93.0329 (1.4), 85.0281 (0.3)	3.44	-0.832
5.	4-*p*-coumaroylquinic acid	$C_{16}H_{18}O_8$	337.0929	337.0924 (14.6), 191.0549 (10.6), 173.0444 (100), 163.0386 (12.3), 119.0488 (15.9), 93.0330 (17.3), 85.0280 (1.5)	3.79	-1.426
6.	5-caffeoylquinic acid isomer	$C_{16}H_{18}O_9$	353.0878	353.0876 (8.5), 191.0552 (100), 179.0340 (0.6), 161.0232 (1.9), 127.0386 (1.6), 111.0437 (1.4), 93.0331 (3.1), 85.0279 (8.8)	3.89	-0.525
7.	5-*p*-coumaroylquinic acid	$C_{16}H_{18}O_8$	337.0929	337.0929 (10.8), 191.0551 (100), 173.0444 (7.4), 163.0388 (6.4), 119.0488 (5.2), 93.0330 (16.4), 85.0278 (4.1)	3.95	-0.091

8.	4-*p*-coumaroylquinic acid isomer	$C_{16}H_{18}O_8$	337.0929	337.0927 (11.8), 191.0554 (4.4), 173.0444 (100), 163.0389 (20.7), 119.0486 (9.3), 93.0330 (25.3), 85.0279 (0.4)	4.03	-0.714
9.	5-feruloylquinic acid	$C_{17}H_{20}O_9$	367.1035	367.1030 (25.1), 193.0498 (12.9), 191.0551 (100), 173.0444 (58.8), 135.0393 (0.4), 134.0359 (14.5), 111.0436 (4.6), 93.0330 (34.5), 85.0280 (4.3)	4.39	-1.322
10.	5-*p*-coumaroylquinic acid isomer	$C_{16}H_{18}O_8$	337.0929	337.0930 (9.3), 191.0551 (100), 173.0444 (2.4), 163.0390 (1.9), 111.0439 (1.3), 93.0330 (4.8), 85.0279 (6.8)	4.61	0.117
11.	3, 5-dicaffeoylquinic acid	$C_{25}H_{24}O_{12}$	515.1195	515.1222 (21.3), 353.0876 (100), 191.0551 (97.0), 179.0339 (50.3), 161.0233 (4.7), 135.0437 (44.7), 111.0434 (1.2), 93.0330 (4.1), 85.0279 (6.1)	5.85	5.223
12.	rosmarinic acid[a]	$C_{18}H_{16}O_8$	359.0772	359.0771 (13.6), 197.0446 (27.8), 179.0340 (11.7), 161.0232 (100), 135.0437 (17.4), 133.0281 (20.7)	6.33	-0.503
			**Proanthocyanidin oligomers (PACs)**			
13.	proanthocyanidin tetramer A, B-type	$C_{60}H_{48}O_{24}$	1151.2463	1151.2465 (100), 1025.2150 (1.2), 999.1996 (4.3), 981.1963 (0.8), 863.1826 (18.4), 711.1345 (3.3), 693.1248 (2.1), 573.1039 (2.4), 447.0723 (2.6), 411.0720 (26.4), 289.0717 (14.5), 285.0403 (15.4), 243.0294 (5.3), 175.0391 (4.3), 161.0232 (9.5), 137.0230 (6.1), 125.0230 (16.7), 109.0280 (5.1)	2.72	0.185
14.	proanthocyanidin dimer B-type	$C_{30}H_{26}O_{12}$	577.1351	577.1349 (100), 451.1022 (5.8), 425.0876 (37.3), 407.0767 (30.6), 289.0715 (46.2), 287.0563 (9.3), 245.0815 (3.2), 229.0502 (2.2), 203.0702 (4.8), 179.0337 (3.9), 175.0387 (5.8), 161.0232 (14.1), 137.0229 (14.1), 151.0390 (7.4), 125.0229 (69.5), 109.0279 (12.9)	2.77	-0.432
15.	proanthocyanidin dimer B-type	$C_{30}H_{26}O_{12}$	577.1351	577.1348 (100), 451.1029 (8.6), 425.0875 (60.2), 407.0769 (42.5), 289.0716 (39.9), 287.0564 (9.5),	2.94	-0.319

				245.0818 (11.2), 203.0705 (4.0), 179.0340 (2.8), 175.0387 (6.3), 161.0229 (16.0), 151.0387 (8.0), 137.0230 (14.2), 125.0229 (58.4), 123.0436 (8.3), 109.0281 (12.1)		
16.	proanthocyanidin pentamer A, B-type	$C_{75}H_{60}O_{30}$	719.1509 $[M-2H]^{2-}$	1151.2419 (2.3), 863.1836 (14.9), 719.1509 (45.5), 656.1359 (2.4), 643.1277 (18.3), 634.1224 (16.3), 575.1196 (11.1), 411.0719 (13.4), 289.0716 (36.4), 285.0404 (14.8), 245.0450 (8.2), 243.0296 (19.1), 175.0391 (10.4), 161.0232 (23.6), 137.0230 (13.4), 125.0230 (100), 109.0280 (8.4)	3.27	0.436
17.	proanthocyanidin trimer B-type	$C_{45}H_{38}O_{18}$	865.1985	865.1989 (100), 739.1671 (4.0), 713.1509 (7.7), 695.1432 (4.6), 577.1335 (14.2), 425.0896 (1.5), 407.0775 (22.4), 289.0717 (26.6), 245.0455 (9.1), 175.0390 (12.8), 161.0233 (29.1), 125.0230 (96.4), 109.0280 (10.2)	3.25	0.373
18.	proanthocyanidin dimer B-type	$C_{30}H_{26}O_{12}$	577.1351	577.1349 (100), 451.1030 (7.5), 425.0879 (43.8), 407.0770 (46.2), 289.0717 (59.5), 287.0558 (8.9), 245.0815 (15.4), 203.0702 (7.1), 175.0388 (7.4), 161.0233 (20.4), 151.0388 (9.0), 137.0231 (15.6), 125.0229 (80.3), 109.0279 (14.7)	3.45	-0.259
19.	proanthocyanidin pentamer A, B-type	$C_{75}H_{60}O_{30}$	719.1509 $[M-2H]^{2-}$	1151.2421 (2.9), 863.1830 (19.9), 719.1515 (63.9), 643.1274 (21.4), 634.1224 (16.3), 575.1192 (23.0), 411.0722 (13.2), 289.0717 (33.1), 285.0402 (16.0), 245.0454 (9.3), 243.0294 (16.8), 175.0389 (10.7), 161.0232 (25.4), 137.0230 (13.1), 125.0230 (100), 109.0279 (9.2)	3.47	0.413
20.	proanthocyanidin tetramer A, B-type	$C_{60}H_{48}O_{24}$	1151.2463	1151.2462 (100), 1025.2218 (1.1), 999.1991 (4.6), 981.1876 (0.8), 863.1829 (19.7), 711.1371 (3.8), 693.1259 (1.9), 573.1035 (2.6), 447.0723 (2.3), 411.0719 (29.6), 407.0770 (5.2), 289.0717 (16.1),	3.53	-0.048

				285.0403 (16.6), 243.0294 (6.2), 217.0501 (2.2), 175.0390 (4.5), 161.0232 (9.6), 137.0230 (5.6), 125.0229 (17.1), 109.0280 (5.0)		
21.	proanthocyanidin trimer B-type	$C_{45}H_{38}O_{18}$	865.1985	865.1985 (100), 739.1729 (3.1), 713.1501 (8.0), 695.1439 (9.8), 577.1339 (9.8), 425.0892 (3.3), 407.0769 (20.6), 289.0717 (21.5), 245.0448 (6.8), 175.0391 (12.1), 161.0233 (29.3), 125.0229 (92.3), 109.0280 (9.6)	3.57	-0.047
22.	proanthocyanidin dimer B-type	$C_{30}H_{26}O_{12}$	577.1351	577.1348 (100), 451.1036 (8.1), 425.0880 (56.4), 407.0768 (50.1), 289.0716 (56.5), 287.0555 (5.9), 245.0818 (14.9), 203.0706 (6.6), 175.0400 (7.3), 161.0232 (21.6), 137.0230 (17.8), 125.0229 (85.4), 109.0280 (15.5)	3.58	-0.319
23.	proanthocyanidin tetramer B-type	$C_{60}H_{50}O_{24}$	576.1273 $[M-2H]^{2-}$	863.1803 (7.8), 576.1268 (42.6), 575.1194 (9.9), 500.1042 (23.0), 491.0988 (9.7), 407.0765 (13.9), 289.0716 (34.5), 287.0558 (12.7), 245.0449 (6.6), 175.0392 (0.9), 161.0230 (23.8), 151.0387 (30.2), 137.0230 (9.6), 125.0230 (100), 109.0280 (8.8)	3.79	-0.320
24.	proanthocyanidin pentamer A, B-type	$C_{75}H_{60}O_{30}$	719.1512 $[M-2H]^{2-}$	1151.2434 (3.4), 863.1833 (19.1), 719.1511 (61.5), 656.1357 (1.7), 643.1277 (21.0), 634.1226 (15.8), 575.1191 (21.2), 411.0724 (13.9), 289.0717 (35.7), 287.0559 (17.8), 285.0403 (17.7), 245.0455 (9.9), 243.0295 (19.1), 175.0390 (9.5), 161.0232 (27.8), 151.0387 (17.4), 137.0230 (12.3), 125.0229 (100), 109.0280 (9.3)	3.84	-0.102
25.	proanthocyanidin trimer A, B-type	$C_{45}H_{36}O_{18}$	863.1829	863.1826 (100), 711.1355 (22.8), 693.1263 (0.9), 573.1046 (10.6), 451.1032 (13.5), 411.0718 (37.4), 299.0565 (4.9), 289.0716 (30.3), 285.0402 (24.0), 245.0814 (9.9), 203.0705 (3.2), 175.0387 (5.9),	3.88	-0.267

				161.0233 (17.1), 149.0232 (10.1), 137.0230 (15.9), 125.0229 (34.0), 109.0279 (12.2)		
26.	Proanthocyanidin trimer A, B-type	$C_{45}H_{36}O_{18}$	863.1829	863.1829 (100), 711.1353 (22.5), 693.1255 (1.2), 573.1043 (7.7), 451.1041 (13.9), 411.0717 (30.2), 299.0555 (4.6), 289.0716 (27.2), 285.0403 (20.4), 245.0817 (7.7), 175.0391 (7.3), 161.0231 (15.8), 137.0230 (15.6), 125.0229 (26.2), 109.0279 (11.8)	4.12	0.033
27.	proanthocyanidin trimer B-type	$C_{45}H_{38}O_{18}$	865.1985	865.1984 (100), 739.1660 (3.8), 713.1519 (9.5), 695.1395 (5.0), 577.1350 (13.1), 425.0881 (2.9), 407.0768 (18.1), 289.0716 (22.1), 245.0451 (8.2), 175.0390 (10.1), 161.0232 (24.2), 125.0229 (83.5), 109.0280 (9.0)	4.18	-0.177
28.	proanthocyanidin hexamer A, B-type	$C_{90}H_{72}O_{36}$	863.1829 $[M-2H]^{2-}$	1151.2462 (4.1), 863.1822 (100), 737.1497 (0.5), 711.1353 (7.4), 575.1191 (9.2), 451.1066 (4.7), 411.0717 (24.7), 289.0717 (26.0), 245.0452 (8.0), 243.0294 (15.8), 217.0497 (4.5), 175.0389 (9.1), 161.0231 (21.3), 137.0229 (9.0), 125.0229 (59.2)	4.27	-0.808
29.	proanthocyanidin tetramer A, B-type	$C_{60}H_{48}O_{24}$	1151.2462	1151.2463 (100), 999.1993 (5.0), 981.1808 (1.3), 861.1679 (5.9), 577.1359 (1.7), 573.1049 (2.5), 529.0773 (4.8), 447.0708 (1.7), 411.0720 (17.1), 407.0768 (9.8), 289.0716 (11.4), 285.0403 (12.9), 243.0296 (6.0), 175.0390 (5.0), 161.0232 (12.2), 137.0230 (6.1), 125.0230 (16.7), 109.0280 (5.1)	4.32	0.065
30.	proanthocyanidin dimer B-type	$C_{30}H_{26}O_{12}$	577.1351	577.1346 (70.7), 451.1035 (7.2), 425.0877 (48.7), 407.0773 (31.6), 289.0717 (61.2), 287.0558 (15.8), 245.0814 (14.0), 203.0711 (5.6), 175.0389 (5.5), 161.0232 (21.9), 137.0231 (4.8), 125.0230 (30.0), 109.0280 (4.9)	4.38	-0.952
31.	proanthocyanidin tetramer B-type	$C_{60}H_{50}O_{24}$	1153.2619	1153.2612 (100), 1027.2277 (2.0), 1001.2164 (3.9), 983.2031 (2.3), 865.1989 (5.6), 863.1824 (7.4),	4.38	-0.935

				739.1644 (1.7), 713.1474 (0.2), 695.1387 (0.3), 577.1339 (6.4), 575.1192 (9.2), 451.1066 (1.5), 425.0841 (1.7), 407.0768 (13.2), 289.0716 (13.0), 287.0560 (15.5), 243.0295 (15.2), 175.0390 (8.6), 161.0232 (20.9), 151.0389 (4.9), 137.0230 (5.9), 125.0229 (66.8), 109.0279 (5.7)		
32.	proanthocyanidin pentamer B-type	$C_{75}H_{62}O_{30}$	720.1590 $[M-2H]^{2-}$	1151.2444 (7.3), 863.1821 (6.9), 720.1573 (49.4), 644.1343 (17.7), 635.1289 (8.4), 575.1196 (10.2), 407.0768 (14.9), 289.0716 (48.7), 287.0558 (20.2), 245.0814 (10.0), 175.0388 (11.4), 151.0387 (16.7), 137.0230 (9.6), 125.0229 (100), 109.0280 (8.9)	4.46	-2.317
33.	proanthocyanidin trimer B-type	$C_{45}H_{38}O_{18}$	865.1985	865.1984 (100), 739.1732 (4.0), 713.1511 (12.6), 695.1409 (2.8), 577.1340 (10.4), 425.0862 (4.1), 407.0770 (17.6), 289.0714 (17.1), 245.0455 (7.0), 175.0382 (11.0), 161.0231 (24.3), 125.0229 (84.4), 109.0279 (9.0)	4.47	-0.107
34.	proanthocyanidin pentamer B-type	$C_{75}H_{62}O_{30}$	720.1590 $[M-2H]^{2-}$	1151.2476 (7.4), 863.1838 (9.5), 720.1588 (45.5), 644.1351 (15.4), 635.1299 (12.1), 575.1190 (11.1), 407.0766 (14.1), 289.0717 (44.0), 287.0559 (19.8), 243.0293 (18.2), 175.0388 (10.1), 161.0231 (25.5), 151.0388 (15.6), 137.0231 (9.2), 125.0229 (100), 109.0280 (8.0)	4.59	-0.303
35.	proanthocyanidin tetramer B-type	$C_{60}H_{50}O_{24}$	576.1273 $[M-2H]^{2-}$	863.1793 (4.3), 576.1259 (23.1), 575.1191 (11.2), 500.1025 (10.0), 491.0983 (6.5), 407.0774 (8.7), 289.0718 (24.0), 287.0559 (9.7), 245.0451 (5.5), 203.0700 (1.3), 175.0392 (5.0), 161.0232 (14.5), 151.0388 (24.4), 137.0229 (6.8), 125.0230 (100), 109.0280 (6.9)	4.60	-1.424
36.	proanthocyanidin pentamer A, B-type	$C_{75}H_{60}O_{30}$	719.1512 $[M-2H]^{2-}$	1151.2510 (4.7), 861.1659 (12.9), 719.1492 (47.3), 656.1356 (07), 643.1275 (20.3), 634.1223 (8.9),	4.68	-2.813

				575.1192 (13.9), 411.0720 (34.5), 289.0717 (22.7), 285.0403 (27.9), 245.0450 (9.3), 175.0390 (8.8), 161.0232 (22.9), 151.0388 (13.8), 137.0229 (8.2), 125.0229 (100), 109.0280 (8.0)		
37.	proanthocyanidin dimer A-type	$C_{30}H_{24}O_{12}$	575.1195	575.1191 (100), 449.0870 (5.3), 423.0709 (5.1), 407.0767 (4.8), 289.0716 (16.1), 285.0403 (26.7), 245.0820 (3.7), 241.0509 (1.8), 229.0517 (0.7), 217.0503 (1.7), 175.0392 (3.6), 163.0025 (5.9), 125.0229 (19.9), 109.0280 (7.2)	4.74	-0.625
38.	proanthocyanidin tetramer A, B-type	$C_{60}H_{48}O_{24}$	575.1195 $[M-2H]^{2-}$	1025.2096 (3.5), 863.1752 (2.1), 861.1694 (4.3), 575.1191 (78.0), 499.0959 (15.1), 490.0891 (11.6), 449.0877 (8.0), 423.0719 (8.0), 407.0784 (7.7), 289.0726 (22.2), 285.0403 (19.8), 243.0294 (10.9), 217.0497 (4.9), 175.0391 (8.6), 151.0387 (22.8), 125.0229 (100), 109.0281 (7.2)	4.83	0.265
39.	proanthocyanidin heptamer A, B-type	$C_{105}H_{86}O_{42}$	1008.7241 $[M-2H]^{2-}$	1439.3157 (10.5), 1153.2573 (11.3), 1008.7236 (100), 945.7078 (5.1), 932.7009 (12.5), 923.1929 (11.4), 863.1818 (28.7), 800.1725 (0.8), 719.1514 (1.3), 577.1339 (21.1), 575.1194 (18.5), 449.0806 (11.0), 423.0723 (6.7), 407.0768 (29.3), 289.0717 (38.7), 287.0559 (49.7), 243.0294 (47.0), 217.0497 (11.4), 175.0391 (23.4), 161.0232 (55.1)	4.90	-2.725
40.	proanthocyanidin pentamer A, B-type	$C_{75}H_{60}O_{30}$	719.1512 $[M-2H]^{2-}$	1151.2349 (3.2), 861.1673 (20.6), 719.1511 (60.3), 643.1273 (30.8), 634.1229 (9.2), 577.1353 (21.1), 411.0719 (38.4), 289.0717 (74.3), 285.0403 (25.8), 245.0817 (10.6), 175.0389 (14.8), 161.0232 (31.8), 151.0388 (22.5), 137.0230 (14.3), 125.0229 (100), 109.0280 (15.3)	4.97	-0.185
41.	proanthocyanidin tetramer A, B-type	$C_{60}H_{48}O_{24}$	575.1195 $[M-2H]^{2-}$	1025.2080 (4.9), 863.1810 (0.9), 575.1187 (69.6), 499.0953 (8.9), 490.0903 (7.5), 449.0883 (7.9),	4.97	-1.372

				411.0710 (16.5), 407.0764 (6.1), 289.0718 (10.7), 285.0402 (30.1), 245.0448 (6.2), 243.0296 (8.5), 217.0500 (3.7), 175.0392 (8.8), 151.0389 (17.4), 137.0230 (5.0), 125.0229 (100), 109.0280 (6.0)		
42.	proanthocyanidin pentamer A, B-type	$C_{75}H_{60}O_{30}$	719.1512 $[M-2H]^{2-}$	1151.2501 (4.6), 863.1818 (21.8), 719.1497 (45.6), 656.1302 (1.6), 643.1263 (15.6), 634.1219 (14.9), 575.1192 (43.1), 411.0724 (15.0), 289.0716 (21.7), 285.0403 (28.9), 245.0451 (8.5), 243.0294 (19.7), 175.0390 (11.7), 161.0232 (26.1), 151.0388 (15.6), 137.0230 (7.3), 125.0230 (100), 109.0280 (7.1)	5.05	2.035
43.	proanthocyanidin trimer B-type	$C_{45}H_{38}O_{18}$	865.1985	865.1983 (100), 739.1671 (12.7), 713.1508 (23.0), 695.1404 (8.4), 577.1348 (19.5), 425.0878 (5.7), 407.0768 (56.3), 289.0716 (49.2), 245.0453 (8.8), 175.0390 (7.8), 161.0232 (30.7), 137.0230 (22.5), 125.0229 (79.0), 109.0279 (12.5)	5.17	-0.237
44.	proanthocyanidin dimer B-type	$C_{30}H_{26}O_{12}$	577.1351	577.1347 (73.9), 451.1037 (9.8), 425.0876 (52.6), 407.0771 (35.2), 289.0717 (59.4), 287.0558 (17.5), 245.0817 (14.1), 203.0705 (5.3), 161.0231 (22.1), 137.0229 (19.5), 125.0229 (100), 109.0279 (15.9)	5.20	-0.440
45.	proanthocyanidin pentamer A-type	$C_{75}H_{58}O_{30}$	718.1434 $[M-2H]^{2-}$	861.1674 (66.9), 718.1436 (28.1), 642.1182 (9.9), 575.1193 (100), 411.0718 (31.1), 289.0716 (17.7), 285.0403 (53.6), 245.0448 (5.8), 243.0294 (12.5), 227.0349 (3.0), 217.0500 (7.1), 175.0388 (11.6), 161.0231 (21.7), 151.0389 (8.2), 137.0231 (5.4), 125.0229 (100), 109.0279 (7.4)	5.23	0.351
46.	proanthocyanidin tetramerA, B-type	$C_{60}H_{48}O_{24}$	575.1195 $[M-2H]^{2-}$	575.1198 (100), 449.0875 (5.1), 423.0727 (18.3), 407.0768 (5.8), 289.0715 (14.4), 285.0405 (31.4), 245.0418 (0.6), 163.0029 (0.2), 149.0233 (2.7), 137.0230 (5.3), 125.0230 (19.0), 109.0281(8.2)	5.35	0.645

47.	proanthocyanidin pentamer A, B-type	$C_{75}H_{60}O_{30}$	1439.3096	1439.3053 (1.4), 1151.2463 (100), 999.1953 (1.2), 863.1790 (1.3), 575.1191 (99.6), 449.0875 (23.9), 423.0719 (37.9), 289.0716 (26.7), 285.0402 (42.0)	5.36	-4.363
48.	proanthocyanidin trimer A, B-type	$C_{45}H_{36}O_{18}$	863.1829	863.1830 (100), 737.1551 (2.6), 711.1302 (33.5), 693.1266 (5.7), 575.1195 (27.0), 449.0883 (10.4), 407.0772 (6.0), 407.0772 (6.0), 289.0716 (20.5), 285.0405 (29.6), 245.0815 (2.5), 203.0705 (3.2), 175.0390 (8.0), 161.0233 (10.6), 137.0226 (5.1), 125.0230 (42.5), 109.0278 (7.8)	5.58	0.093
49.	proanthocyanidin dimer A-type	$C_{30}H_{24}O_{12}$	575.1195	575.1191 (100), 449.0880 (8.7), 423.0725 (14.9), 407.0777 (11.1), 289.0717 (13.7), 285.0402 (29.5), 245.0816 (4.3), 241.0500 (3.2), 217.0502 (3.8), 163.0024 (8.4), 137.0230 (5.0), 125.0229 (25.7), 109.0279 (7.5)	5.83	-0.729
50.	proanthocyanidin tetramer A, B-type	$C_{60}H_{46}O_{24}$	1149.2306	1149.2303 (100), 997.1847 (16.5), 575.1201 (36.5), 449.0896 (9.2), 411.0713 (21.2), 289.0712 (16.1), 285.0404 (33.8), 243.0306 (5.7), 217.0508 (4.8), 175.0397 (6.3), 161.0235 (13.3), 149.0237 (4.9), 137.0231 (4.7), 125.0230 (20.3), 109.0283 (5.2)	6.22	-0.325
51.	proanthocyanidin pentamer A-type	$C_{75}H_{58}O_{30}$	718.1434 $[M-2H]^{2-}$	861.1667 (58.3), 718.1426 (49.3), 642.1199 (40.8), 575.1191 (100), 411.0719 (58.3), 289.0717 (24.8), 285.0403 (64.0), 245.0441 (6.1), 243.0293 (15.2), 217.0501 (10.3), 175.0387 (14.6), 161.0231 (39.4), 151.0387 (19.2), 137.0231 (22.4), 125.0229 (90.8), 109.0280 (13.1)	6.36	-1.098
			**Flavonoids**			
52.	gallocatechin	$C_{15}H_{14}O_7$	305.0667	305.0665 (100), 261.0771 (7.2), 219.0654 (17.1), 243.0669 (1.7), 179.0340 (25.9), 167.0338 (24.2), 137.0231 (29.6), 125.0230 (98.8)	1.79	-0.577

53.	(+) catechin[a]	$C_{15}H_{14}O_6$	289.0718	289.0717 (100), 245.0816 (40.0), 203.0704 (20.5), 179.0339 (11.5), 137.0231 (10.8), 123.0437 (23.1), 109.0280 (35.5)	3.12	-0.247
54.	naringenin 6, 8 di*C*-hexoside	$C_{27}H_{32}O_{15}$	595.1678	595.1667 (100), 505.1345 (0.8), 475.1243 (2.7), 415.1037 (8.1), 385.0927 (31.2), 355.0822 (33.4), 271.0617(0.7), 235.0247 (2.4), 151.0022 (1.0), 119.0488 (13.7), 107.0122 (3.1)	3.64	-0.157
55.	eryodictiol *O*-hexoside 1	$C_{21}H_{22}O_{11}$	449.1089	449.1090 (56.1), 287.0559 (99.8), 259.0609 (100), 243.0659 (7.4), 178.9976 (15.7), 151.0024 (16.6), 125.0229 (39.9), 107.0123 (7.4)	3.73	-0.233
56.	gossypetin *O*-hexoside 1	$C_{21}H_{20}O_{13}$	479.0831	479.0825 (100), 317.0289 (27.7), 316.0222 (68.9), 287.0195 (19.8), 271.0246 (18.0), 259.0241 (3.3), 243.0290 (4.4), 227.0344 (2.2), 165.9896 (5.1), 139.0029 (7.8), 109.9995 (5.2)	3.90	-1.365
57.	epicatechin	$C_{15}H_{14}O_6$	289.0718	289.0717 (100), 245.0815 (39.7), 203.0705 (20.7), 179.0340 (12.1), 137.0231 (16.4), 123.0437 (36.1), 109.0280 (48.4)	3.90	-0.247
58.	gossypetin *O*-rutinoside	$C_{27}H_{30}O_{17}$	625.1410	625.1406 (100), 317.0294 (25.9), 316.0222 (53.3), 287.0192 (11.9), 271.0248 (11.2), 243.0293 (3.2), 165.9996 (6.2), 139.0029 (10.2), 109.9994 (4.24)	4.01	-0.740
59.	eryodictiol *O*-hexoside 1	$C_{21}H_{22}O_{11}$	449.1089	449.1087 (100), 287.0558 (27.4), 269.0454 (42.1), 259.0609 (51.1), 243.0659 (3.8), 178.9975 (9.8), 151.0022 (11.9), 125.0228 (53.9), 107.0123 (4.4)	4.02	-0.552
60.	gossypetin *O*-hexoside 2	$C_{21}H_{20}O_{13}$	479.0831	479.0827 (100), 317.0290 (30.1), 316.0222 (73.4), 287.0194 (17.3), 271.0246 (15.0), 243.0296 (4.2), 165.9894 (3.9), 139.0023 (6.7), 109.9993 (6.7)	4.04	-0.801
61.	gossypetin *O*-pentoside 1	$C_{20}H_{18}O_{12}$	449.0725	449.0722 (100), 317.0291 (27.4), 316.0222 (63.8), 287.0197 (18.8), 271.0247 (17.60), 243.0291 (4.5), 199.0392 (2.0), 178.9975 (4.2), 139.0023 (8.3), 109.9995 (4.7)	4.18	-0.688
62.	gossypetin *O*-pentoside 2	$C_{20}H_{18}O_{12}$	449.0725	449.0722 (100), 317.0287 (15.0), 316.0222 (62.5), 299.0222 (0.6), 287.0196 (17.0), 271.0247 (15.0),	4.24	-0.777

				227.0346 (1.4), 199.0394 (1.6), 178.9976 (3.4), 165.9896 (5.0), 139.0022 (7.9), 109.9993 (4.6)		
63.	galangin methyl ether *O*-hexoside	$C_{23}H_{24}O_{12}$	491.1195	491.1194 (13.9), 445.1136 (21.9), 283.0609 (100), 268.0376 (6.9), 253.0512 (0.3), 240.0424 (11.8), 211.0393 (1.2)	4.36	-0.100
64.	quercetin *O*-dihexoside	$C_{27}H_{30}O_{17}$	625.1410	625.1407 (100), 463.0896 (2.1), 301.0333 (20.3), 300.0274 (96.2), 271.0247 (17.6), 255.0296 (10.0), 243.0296 (9.0), 227.0345 (2.1), 178.9075 (3.9), 151.0023 (5.2), 107.0125 (2.1)	4.40	-0.532
65.	saponarin[a]	$C_{27}H_{30}O_{15}$	593.1512	593.1512 (100), 473.1068 (5.4), 431.0989 (8.5), 341.0676 (3.6), 311.0563 (26.5), 297.0404 (15.4), 283.0609 (7.9), 269.0457 (2.1), 117.0330 (5.4)	4.40	0.096
66.	gossypetin *O*-pentoside 3	$C_{20}H_{18}O_{12}$	449.0725	449.0721 (100), 317.0296 (41.0), 316.0221 (51.6), 287.0196 (14.6), 271.0247 (14.3), 243.0293 (4.4), 227.0353 (1.6), 199.0395 (2.4), 165.9897 (7.6), 139.0023 (14.7), 109.9994 (7.7)	4.50	-0.911
67.	myricetin O-hexoside 1	$C_{21}H_{20}O_{13}$	479.0831	479.0826 (100), 317.0283 (19.4), 316.0222 (88.4), 287.0194 (13.3), 271.0245 (19.8), 259.0251 (4.7), 243.0298 (3.1), 178.9973 (3.1), 151.0024 (3.8), 107.0123 (1.0)	4.51	-0.989
68.	myricetin *O*-hexoside 2	$C_{21}H_{20}O_{13}$	479.0831	479.0826 (100), 317.0283 (17.6), 316.0222 (74.0), 287.0196 (11.8), 271.0246 (20.3), 259.0244 (4.6), 178.9973 (3.4), 151.0022 (3.6), 107.0119 (1.2)	4.59	-1.052
69.	luteolin *O*-hexosyl-*O*-hexuronide	$C_{27}H_{28}O_{17}$	623.1254	623.1243 (69.8), 447.0935 (2.3), 285.0402 (100), 267.0296 (0.4), 241.0493 (1.0), 199.0393 (1.9), 175.0389 (2.3), 151.0023 (3.9), 133.0282 (6.6), 107.0121 (2.6)	4.62	-1.673
70.	myricetin *O*-pentoside 1	$C_{20}H_{18}O_{12}$	449.0725	449.0722 (100), 317.0274 (14.1), 316.0221 (82.6), 287.0196 (14.8), 271.0246 (22.8), 259.0244 (5.6),	4.98	-0.777

				243.0291 (2.9), 178.9977 (2.2), 151.0023 (3.3), 107.0118 (1.3)		
71.	myricetin *O*-pentoside 2	$C_{20}H_{18}O_{12}$	449.0725	449.0721 (100), 317.0289 (27.3), 316.0221 (81.9), 287.0195 (13.7), 271.0246 (21.9), 259.0247 (5.0), 243.0292 (3.4), 178.9975 (4.0), 151.0025 (5.3), 107.0123 (1.7)	5.03	-0.911
72.	rutin[a]	$C_{27}H_{30}O_{16}$	609.1464	609.1457 (100), 301.0346 (31.6), 300.0273 (53.7), 271.0247 (20.1), 255.0297 (11.2), 243.0291 5.8), 227.0350 (1.9), 211.0391 (0.5), 199.0394 (0.3), 178.9974 (3.0), 151.0025 (4.6), 121.0280 (1.0), 107.0123 (1.9)	5.10	-0.686
73.	quercetin *O*-pentosylhexoside	$C_{26}H_{28}O_{16}$	595.1305	595.1303 (100), 463.0880 (74.2), 301.0380 (66.4), 271.0247 (32.7), 255.0297 (17.5), 243.0296 (9.2), 178.9978 (6.5), 151.0024 (17.3), 107.0124 (7.1)	5.11	-0.248
74.	isoquercitrin[a]	$C_{21}H_{20}O_{12}$	463.0886	463.0880 (100), 301.0345 (35.6), 300.0273 (71.8), 271.0246 (34.4), 255.0294 (16.6), 243.0294 (8.3), 227.0344 (3.1), 178.9975 (1.9), 151.0023 (5.8), 121.0280 (0.8), 107.0123 (2.4)	5.20	-0.473
75.	quercetin *O*-hexuronide	$C_{21}H_{18}O_{13}$	477.0675	477.0670 (91.2), 301.0351 (100), 245.0442 (2.6), 178.9978 (12.3), 151.0028 (16.9), 121.0281 (9.1), 107.0123 (4.0)	5.22	-1.056
76.	luteolin 7-*O*-rutinoside[a]	$C_{27}H_{30}O_{15}$	593.1512	593.1510 (100), 285.0402 (95.1), 255.0291 (1.8), 239.0368 (0.5), 227.0331 (0.7), 217.0499 (0.9), 199.0386 (1.4), 175.0388 (2.7), 151.0025 (3.2), 133.0279 (4.3), 107.0119 (2.1)	5.26	-0.326
77.	hyperoside[a]	$C_{21}H_{20}O_{12}$	463.0887	463.0880 (100), 300.0274 (75.7), 271.0246(35.3), 255.0296 (16.5), 243.0294 (9.2), 227.0348 (2.9), 178.9975 (2.6), 151.0025 (4.9), 121.0278 (1.5), 107.0118 (2.8)	5.30	-0.538

78.	luteolin *O*-hexuronide	$C_{21}H_{18}O_{12}$	461.0725	461.0724 (61.2), 285.0403 (100), 267.0301 (0.4), 243.0292 (1.2), 217.0502 (1.3), 199.0388 (2.0), 175.0385 (2.8), 151.0024 (5.2), 133.0281 (8.8), 107.0124 (2.0)	5.39	-0.215
79.	luteolin 7-*O*-glucoside[a]	$C_{21}H_{20}O_{11}$	447.0933	447.0929(100), 285.0401 (82.1), 256.0370 (3.1), 227.0347 (1.0), 199.0391 (1.8), 175.0388 (1.2), 151.0025 (4.9), 133.0280 (3.9), 107.0121 (2.8)	5.40	-0.860
80.	luteolin O-deoxyhexosyl-O-hexoside	$C_{27}H_{30}O_{15}$	593.1512	593.1512 (100), 447.0945 (2.3), 285.0402 (31.2), 256.0370 (1.0), 239.0344 (0.5), 227.0343 (0.7), 211.0388 (0.7), 199.0398 (0.7), 151.0025 (2.8), 133.0281 (2.7), 107.0121 (1.7)	5.44	-0.022
81.	quercetin 3-*O*-pentoside 1	$C_{20}H_{18}O_{11}$	433.0776	433.0771 (100), 301.0347 (36.6), 300.074 (56.3), 271.0247 (31.1), 255.0297 (13.5), 227.0341 (2.6), 178.9975 (2.1), 151.0028 (4.5), 121.0279 (1.3), 107.0124 (1.4)	5.52	-1.234
82.	quercetin 3-O-pentoside 2	$C_{20}H_{18}O_{11}$	433.0776	433.0771 (100), 301.0336 (27.9), 300.0273 (90.4), 271.0247 (30.6), 255.0295(13.7), 227.0340 (2.3), 178.9976 (1.7), 151.0024 (3.9), 121.0280 (0.5), 107.0123 (0.9)	5.63	-1.233
83.	kaempferol 3-*O*-rutinoside[a]	$C_{27}H_{30}O_{15}$	593.1512	593.1511 (100), 285.0402 (64.4), 255.0296 (27.9), 227.0345 (20.7), 211.0393 (0.7), 185.0601 (2.2), 151.0021 (1.6), 135.0074 (0.7), 107.0119 (1.6)	5.63	-0.225
84.	quercetin *O*-pentoside 3	$C_{20}H_{18}O_{11}$	433.0776	433.0771 (100), 301.0348 (84.5), 300.073 (77.5), 271.0247 (36.9), 255.0295 (18.3), 227.0342(3.2), 178.9975 (5.1), 151.0023 (9.7), 121.0278 (2.6), 107.0123 (3.7)	5.74	-1.119
85.	isorhamnetin 3-*O*-rutinoside[a]	$C_{28}H_{32}O_{16}$	623.1618	623.1607 (100), 315.0507 (76.7), 300.0263 (13.2), 299.0196 (11.7), 271.0257 (26.5), 243.0296 (14.3), 199.0388 (4.2), 151.0029 (1.3), 133.0279 (5.2)	5.80	-0.802

86.	quercitrin[a]	$C_{21}H_{20}O_{11}$	447.0933	447.0929 (100), 301.0348 (52.3), 300.0274 (60.6), 271.0247 (27.8), 255.0296 (12.7), 243.0294 (7.1), 227.0344 (2.5), 211.0390 (0.7), 199.0390 (0.8), 178.9974 (2.9), 163.0025 (2.0), 151.0024 (7.0), 121.0280 (1.9), 107.0123 (3.0)	5.92	-0.860
87.	isorhamnetin 3-*O*-glucoside[a]	$C_{22}H_{22}O_{12}$	477.1044	477.1035 (100), 315.0505 (9.8), 314.0432 (32.2), 299.0187 (2.0), 271.0246 (16.8), 257.0450 (3.2), 243.0294 (11.8), 199.0398 (2.6), 178.9977 (1.1), 151.0024 (1.3), 107.0122 (0.9)	6.04	-0.753
88.	luteolin *O*-hexoside	$C_{21}H_{20}O_{11}$	447.0933	477.0929 (29.4), 285.0403 (100), 271.0256 (1.7), 243.0298 (0.8), 217.0501 (1.4), 199.0391 (2.4), 175.0390 (2.6), 151.0023 (5.5), 133.0281 (9.8), 107.0122 (2.1)	6.06	-0.860
89.	apigenin 7-*O*-glucoside[a]	$C_{21}H_{20}O_{10}$	431.0983	431.0981 (100), 269.0431 (93.1), 240.0426 (6.9), 211.0407 (2.2), 151.0023 (4.6), 117.0334 (1.3), 107.0120 (3.5)	6.09	-0.603
90.	apigenin O-hexuronide	$C_{21}H_{18}O_{11}$	445.0776	445.0771 (33.0), 269.0453 (100), 225.0548 (1.9), 201.0546 (1.1), 175.0236 (17.1), 151.0024 (2.3), 117.0331 (6.9), 107.0121 (3.3)	6.13	-1.291
91.	gossypetin	$C_{15}H_{10}O_{8}$	317.0303	317.0305 (100), 299.0199 (9.2), 271.0242 (6.6), 255.0300 (2.8), 247.0611 (7.1), 178.9980 (1.6), 231.0301 (3.3), 227.0347 (2.4), 166.9975 (16.7), 165.0181 (6.0), 139.0023 (25.0), 109.0280 (10.4)	6.19	0.724
92.	chrysoeriol *O*-hexoside	$C_{22}H_{22}O_{11}$	461.1089	461.1091 (100), 446.0851 (13.3), 299.0556 (7.5), 298.0473 (9.1), 284.0305 (9.2), 283.0245 (32.1), 255.0297 (24.1), 163.0027 (5.4)	6.31	0.424
93.	quercetin *O*-caffeoylhexoside	$C_{30}H_{26}O_{15}$	625.1199	625.1196 (100), 463.0880 (33.4), 301.0349 (50.7), 300.0274 (52.4), 271.0246 (36.0), 255.0296 (15.7), 243.0293 (6.4), 178.9972 (4.8), 121.0276 (3.1), 151.0022 (10.6), 107.0123 (4.1)	6.44	-0.517

94.	kaempferol *O*-deoxyhexoside	$C_{21}H_{20}O_{11}$	431.0983	431.0979 (100), 285.0401 (73.0), 255.0296 (37.0), 227.0343 (30.2), 211.0394 (1.5), 151.0023 (0.6), 135.0070 (1.1), 107.0119 (1.1)	6.61	-1.02
95.	isorhamnetin *O*-deoxyhexoside	$C_{22}H_{22}O_{11}$	461.1089	461.1090 (100), 315.0511 (23.9), 314.0432 (49.1), 300.0251 (2.7), 285.0415 (5.5), 271.0253 (17.8), 257.0443 (5.7), 243.0293 (24.5), 227.0347 (3.2), 199.0388 (3.1), 151.0022 (2.9)	6.75	0.098
96.	luteolin *O*-acetylhexoside	$C_{23}H_{22}O_{12}$	489.1038	489.1035 (6.9), 285.0402 (100), 256.0383 (0.2), 243.0297 (0.4), 221.0687 (0.2), 217.0501 (1.4), 175.0390 (3.1), 151.0023 (5.1), 133.0280 (11.1), 107.0123 (3.0)	6.87	-0.816
97.	quercetin *O*-pentoside 4	$C_{20}H_{18}O_{11}$	433.0776	433.0771 (62.5), 301.0352 (100), 273.0411 (1.1), 229.0418 (0.5), 178.9975 (12.1), 151.0023 (33.1), 121.0280 (8.2), 107.0123 (14.0)	7.04	-1.119
98.	luteolin[a]	$C_{15}H_{10}O_6$	285.0405	285.0403 (100), 267.0303 (0.2), 241.0499 (0.5), 217.0503 (1.1), 199.0393 (2.0), 175.0391 (2.7), 151.0023 (5.1), 133.0281 (23.4), 121.0279 (1.2), 107.0123 (4.1)	7.58	-0.741
99.	quercetin[a]	$C_{15}H_{10}O_7$	301.0354	301.0352 (100), 273.0399 (2.8), 257.0452 (0.5), 245.0451 (0.9), 229.0497 (0.9), 178.9975 (20.7), 161.0227 (0.9), 151.0023 (45.7), 121.0280 (12.6), 107.0280 (16.4)	7.62	-0.617
100.	apigenin[a]	$C_{15}H_{10}O_5$	269.0457	269.0454 (100), 225.0551 (1.6), 201.050 (1.0), 181.0649 (1.0), 151.0025 (4.9), 117.0331 (16.8), 107.0123 (5.3)	8.63	-0.619
101.	kaempferol[a]	$C_{15}H_9O_7$	285.0406	285.0401 (100), 257.0449 (0.7), 239.0347 (0.8), 227.0338 (0.8), 211.0397 (1.1), 178.9911 (0.2), 151.0021 (0.8), 107.0124 (1.2)	8.83	-1.162
102.	hispidulin (scutellarein-6-methyl ether)[a]	$C_{16}H_{12}O_6$	299.0563	299.0558 (71.4), 284.0324 (100), 255.0295 (1.1), 227.0346 (3.5), 211.0394 (2.3), 165.9897 (1.0), 164.0106 (1.8), 136.9866 (15.1), 117.0331 (1.8)	8.85	-1.275

103.	quercetin O-cinnamoylhexoside1	$C_{30}H_{26}O_{13}$	593.1301	593.1296 (100), 301.0342 (22.8), 300.0274 (72.5), 271.0246 (38.3), 255.0298 (10.2), 243.0295 (10.5), 178.9977 (3.8), 163.0024 (1.4), 151.0022 (4.7), 135.0068 (0.7). 121.0277 (0.5), 107.0119 (1.5)	8.86	-0.512
104.	chrysoeriol[a]	$C_{16}H_{12}O_6$	299.0562	299.0556(100), 284.0323 (71.6), 256.0372 (10.8), 239.0347 (1.0), 227.03455 (4.1), 211.0388 (2.0), 151.0027 (2.1), 107.0123 (1.7)	8.93	-1.275
105.	quercetin O-cinnamoylhexoside2	$C_{30}H_{26}O_{13}$	593.1301	593.1299 (100), 301.0338 (21.6), 300.0274 (83.7), 271.0247 (40.7), 255.0296 (11.5), 243.0295 (11.5), 227.0345 (3.7), 199.0389 (1.1), 178.9977 (3.1), 151.0024 (5.5), 135.0071 (0.5). 107.0120 (1.9)	9.13	-0.310

[a] identified by comparison with an authentic standard

Table S2. Semiquantitative content of annotated compounds in ES1 and ES2.

№	Identified/tentatively annotated compound	Content in ES2 [%]	Content in ES1 [%]
	**Acylquinic acids**		
1.	neochlorogenic acid[a]	2.17	2.06
2.	chlorogenic acid[a]	43.29	55.00
3.	4-caffeoylquinic acid	20.72	17.73
4.	3-feruloylquinic acid	0.42	n.d.
5.	4-*p*-coumaroylquinic acid	1.72	0.89
6.	5-caffeoylquinic acid isomer	7.74	5.71
7.	5-*p*-coumaroylquinic acid	9.60	8.60
8.	4-*p*-coumaroylquinic acid isomer	3.53	2.70
9.	5-feruloylquinic acid	1.94	1.84
10.	5-*p*-coumaroylquinic acid isomer	1.70	1.58
11.	3, 5-dicaffeoylquinic acid	0.98	0.27
12.	rosmarinic acid	6.48	3.56
	**Proanthocyanidin oligomers (PACs)**		
13.	proanthocyanidin tetramer A, B-type	0.73	0.48
14.	proanthocyanidin dimer B-type	2.25	1.35
15.	proanthocyanidin dimer B-type	0.72	0.60
16.	proanthocyanidin pentamer A, B-type	0.52	n.d.
17.	proanthocyanidin trimer B-type	0.22	n.d.
18.	proanthocyanidin dimer B-type	2.93	1.90
19.	proanthocyanidin pentamer A, B-type	2.64	1.79
20.	proanthocyanidin tetramer A, B-type	0.40	0.22
21.	proanthocyanidin trimer B-type	0.75	0.48
22.	proanthocyanidin dimer B-type	7.56	5.27
23.	proanthocyanidin tetramer B-type	0.31	0.27
24.	proanthocyanidin pentamer A, B-type	0.60	0.59
25.	proanthocyanidin trimer A, B-type	4.80	4.13

26.	Proanthocyanidin trimer A, B-type	10.40	10.81
27.	proanthocyanidin trimer B-type	0.91	0.80
28.	proanthocyanidin hexamer A, B-type	0.61	n.d.
29.	proanthocyanidin tetramer A, B-type	1.07	n.d.
30.	proanthocyanidin dimer B-type	2.06	1.62
31.	proanthocyanidin tetramer B-type	0.46	0.38
32.	proanthocyanidin pentamer B-type	2.10	0.51
33.	proanthocyanidin trimer B-type	0.53	0.41
34.	proanthocyanidin pentamer B-type	1.37	1.266
35.	proanthocyanidin tetramer B-type	0.33	0.42
36.	proanthocyanidin pentamer A, B-type	0.69	0.57
37.	proanthocyanidin dimer A-type	9.60	3.00
38.	proanthocyanidin tetramer A, B-type	5.46	2.09
39.	proanthocyanidin heptamer A, B-type	0.40	0.51
40.	proanthocyanidin pentamer A, B-type	0.57	1.02
41.	proanthocyanidin tetramer A, B-type	2.08	1.87
42.	proanthocyanidin pentamer A, B-type	0.53	0.48
43.	proanthocyanidin trimer B-type	0.49	0.43
44.	proanthocyanidin dimer B-type	3.66	3.72
45.	proanthocyanidin pentamer A-type	1.76	3.02
46.	proanthocyanidin tetramerA, B-type	23.10	43.69
47.	proanthocyanidin pentamer A, B-type	0.44	0.06
48.	proanthocyanidin trimer A, B-type	3.69	3.78
49.	proanthocyanidin dimer A-type	1.98	1.891
50.	proanthocyanidin tetramer A, B-type	0.67	0.46
51.	proanthocyanidin pentamer A-type	0.31	0.24
	**Flavonoids**		
52.	gallocatechin	0.44	0.18
53.	(+) catechin	8.31	5.56

54.	naringenin 6, 8 di*C*-hexoside	0.12	0.04
55.	eryodictiol *O*-hexoside 1	0.16	0.05
56.	gossypetin *O*-hexoside 1	1.35	0.61
57.	epicatechin	13.91	12.15
58.	gossypetin *O*-rutinoside	0.05	0.02
59.	eryodictiol *O*-hexoside 1	0.38	0.15
60.	gossypetin *O*-hexoside 2	0.64	0.26
61.	gossypetin *O*-pentoside 1	0.35	0.20
62.	gossypetin *O*-pentoside 2	0.68	0.29
63.	galangin methyl ether *O*-hexoside	1.67	1.03
64.	quercetin *O*-dihexoside	0.31	0.12
65.	saponarin[a]	0.12	0.07
66.	gossypetin *O*-pentoside 3	0.92	0.43
67.	myricetin O-hexoside 1	0.30	0.18
68.	myricetin *O*-hexoside 2	0.17	0.06
69.	luteolin *O*-hexosyl-*O*-hexuronide	0.05	0.02
70.	myricetin *O*-pentoside 1	0.14	0.05
71.	myricetin *O*-pentoside 2	0.40	0.14
72.	rutin	2.85	1.60
73.	quercetin *O*-pentosylhexoside	0.05	n.d.
74.	isoquercitrin	7.59	7.89
75.	quercetin *O*-hexuronide	0.08	0.05
76.	luteolin 7-*O*-rutinside	0.07	0.04
77.	hyperoside	5.40	5.21
78.	luteolin *O*-hexuronide	0.23	0.20
79.	luteolin 7-*O*-glucoside	1.07	0.57
80.	luteolin O-deoxyhexosyl-O-hexoside	0.08	0.03
81.	quercetin 3-*O*-pentoside 1	6.14	5.33
82.	quercetin 3-*O*-pentoside 2	4.29	4.84

83.	kaempferol 3-*O*-rutinoside	0.07	0.04
84.	quercetin *O*-pentoside 3	9.91	10.27
85.	isorhamnetin 3-*O*-rutinoside	0.15	0.07
86.	quercitrin	14.48	25.20
87.	isorhamnetin 3-*O*-glucoside	0.28	0.15
88.	luteolin *O*-hexoside	2.14	1.05
89.	apigenin 7-*O*-glucoside	0.48	0.21
90.	apigenin *O*-hexuronide	0.95	0.40
91.	gossypetin	0.06	0.04
92.	chrysoeriol *O*-hexoside	0.28	0.11
93.	quercetin *O*-caffeoylhexoside	0.06	0.02
94.	kaempferol *O*-deoxyhexoside	2.19	0.90
95.	isorhamnetin *O*-deoxyhexoside	0.17	0.08
96.	luteolin *O*-acetylhexoside	1.33	0.60
97.	quercetin *O*-pentoside 4	3.54	2.83
98.	luteolin[a]	2.01	2.85
99.	quercetin[a]	2.24	2.40
100.	apigenin[a]	0.85	3.18
101.	kaempferol[a]	0.12	1.19
102.	hispidulin (scutellarein-6-methyl ether)	0.45	0.56
103.	quercetin *O*-cinnamoylhexoside1	0.09	0.07
104.	chrysoeriol	0.16	0.28
105.	quercetin *O*-cinnamoylhexoside2	0.13	0.11

n.d.-not detected

Table S3. Retention times and characteristic ions of mass spectra of steroids and triterpenoids identified in *E. spiculifolia* diethyl ether extracts.

Retention time (min)	Compound	Molecular formula/ mass	Mass spectrum *m*/*z* (relative intensity)
33.3	Campesterol	$C_{28}H_{48}O$ 400.7	400 (30), 107 (51), 105 (55), 95 (49), 83 (45), 81 (64), 71 (62), 57 (77), 55 (77), 43 (100), 41 (52)
35.9	Sitosterol	$C_{29}H_{50}O$ 414.7	414 (29), 145 (54), 107 (59), 105 (60), 95 (54), 91 (49), 81 (57), 57 (68), 55 (70), 43 (100), 41 (44)
36.1	Sitostanol	$C_{29}H_{52}O$ 420.7	416 (31), 215 (82), 109 (58), 107 (83), 95 (81), 93 (64), 81 (84), 69 (60), 57 (64), 55 (81), 43 (100)
36.4	Germanicol	$C_{30}H_{50}O$ 426.7	426 (1), 204 (100), 177 (85), 189 (75), 95 (58), 55 (46), 205 (44), 81 (42), 109 (40), 69 (39), 107 (37)
36.8	β-Amyrin	$C_{30}H_{50}O$ 426.7	426 (27), 219 (18), 218 (100), 203 (49), 189 (17), 135 (11), 109 (13), 105 (12), 95 (15), 81 (18), 69 (14)
37.6	α-Amyrenone	$C_{30}H_{48}O$ 424.7	424 (12), 219 (19), 218 (100), 203 (24), 189 (16), 135 (19), 133 (18), 122 (18), 119 (17), 95 (16), 55 (18)
38.4	α-Amyrin	$C_{30}H_{50}O$ 426.7	426 (4), 218 (100), 203 (20), 189 (36), 135 (35), 121 (32), 109 (32), 107 34), 95 (40), 81 (33), 55 (31)
39.1	Tremulone (stigmasta-3,5-dien-7-one)	$C_{29}H_{46}O$ 410.7	410 (32), 187 (27), 174 (100), 161 (37), 159 (26), 91 (28), 57 (28), 55 (37), 43 (44), 41 (28)
40.2	24-Methylenecycloartanol	$C_{31}H_{52}O$ 440.7	440 (5), 121 (60), 119 (55), 109 (62), 107 (76), 105 (57), 95 (98), 93 (64), 81 (72), 69 (99), 55 (100)
43.1	Taraxasterol	$C_{30}H_{50}O$ 426.7	426 (14), 207 (57), 189 (100), 135 (51), 121 (74), 109 (57), 107 (62), 95 (70), 93 (47), 81 (48), 67 (43)
43.6	α-Amyrin acetate	$C_{32}H_{52}O_2$ 468.7	468 (10), 219 (35), 218 (100), 203 (25),189 (28), 125 (23), 120 (24), 107 (23), 93 (23), 69 (24), 43 (28)
46.1	Oleanolic aldehyde	$C_{30}H_{48}O_2$ 440.7	440 (2), 232 (28), 207 (20), 204 (39), 203 (100), 189 (29), 105 (18), 81 (19), 69 (20), 55 (29)
48.5	Ursolic aldehyde	$C_{30}H_{48}O_2$ 440.7	440 (1), 207 (26), 204 (23), 203 (100), 133 (42), 119 (18), 105 (18), 95 (18), 81 (18), 55 (18), 43 (20)
51.0	Erythrodiol	$C_{30}H_{48}O_2$ 442.7	442 (1), 204 (17), 203 (100), 133 (7), 119 (9), 105 (8), 95 (9), 93 (8), 81 (8), 69 (9), 55 (8)
53.8	Uvaol	$C_{30}H_{48}O_2$ 442.7	442 (1), 207 (13), 204 (17), 203 (100), 133 (33), 119 (13), 105 (11), 95 (12), 81 (10), 69 (10)
			**Acids (analyzed after methylation)**
40.6	Olean-2,12-dien-28-oic acid methyl ester	$C_{32}H_{48}O_2$ 452.7	452(11), 425 (9), 263 (11), 262 (61), 221 (14), 203 (100), 190 (15), 189 (22), 133 (14), 119 (12)
42.9	Ursa-2,12-dien-28-oic acid methyl ester	$C_{32}H_{48}O_2$ 452.7	452 (12), 425 (9), 263 (20), 262 (100),221 (27), 203 (79), 190 (18), 189 (27), 133 (58), 119 (23)
43.4	3-Oxo-olean-12-en-28-oic acid methyl ester	$C_{31}H_{48}O_3$ 468.3	468 (6), 262 (32), 204 (17), 203 (100), 202 (21), 189 (29),133 (17), 119 (14), 105 (12), 55 (12)

44.9	Oleanolic acid methyl ester	$C_{31}H_{50}O_3$ 470.4	470 (1), 262 (48), 207 (13), 204 (16), 203 (100), 202 (21), 189 (22), 133 (17), 119 (13), 105 (14)
46.0	3-Oxo-urs-12-en-28-oic acid methyl ester	$C_{31}H_{48}O_3$ 468.3	468 (3), 263 (21), 262 (96), 249 (20), 204 (17), 203 (100), 189 (29), 133 (79), 119 (30), 105 (19)
48.6	Ursolic acid methyl ester	$C_{31}H_{50}O_3$ 470.4	470 (1), 263 (20), 262 (100), 207 (32), 203 (93), 189 (29), 133 (76), 119 (34), 105 (21), 95 (18)
50.3	Unidentified acid methyl ester*	$C_{31}H_{48}O_3$ 468.0	468 (18), 260 (42), 247 (50), 201 (100), 187 (31), 171 (27), 145 (21), 131 (25), 69 (18)

*tentatively identified as micromeric acid methyl ester.

References

1. Uysal, S.; Zengin, G.; Locatelli, M.; Bahadori, M. B.; Mocan, A.; Bellagamba, G.; De Luca, E.; Mollica, A.; Aktumsek, A., Cytotoxic and enzyme inhibitory potential of two Potentilla species (P. speciosa L. and P. reptans Willd.) and their chemical composition. *Frontiers in pharmacology* **2017,** 8, 290.
2. Grochowski, D. M.; Uysal, S.; Aktumsek, A.; Granica, S.; Zengin, G.; Ceylan, R.; Locatelli, M.; Tomczyk, M., In vitro enzyme inhibitory properties, antioxidant activities, and phytochemical profile of Potentilla thuringiaca. *Phytochemistry Letters* **2017,** 20, 365-372.
3. Roh, C.; Jung, U., Screening of crude plant extracts with anti-obesity activity. *International Journal of Molecular Sciences* **2012,** 13, (2), 1710-1719.
4. Kurt-Celep, I.; Nilofar; Cetiz, M. V.; Zheleva-Dimitrova, D.; Gevrenova, R.; Celep, E.; Sinan, K. I.; Yildiztugay, E.; Ferrante, C.; Zengin, G., From small-scale studies to an encompassing view: Inhibiting inflammation and clinically relevant enzymes with various extracts of Primula vulgaris using in vitro and in silico techniques. *Food Frontiers* **2025,** 6, (1), 329-359.
5. Lescano, L.; Cziáky, Z.; Kurt-Celep, İ.; Zengin, G.; Fernandes, E.; Trentin, R.; Pereira, C. G.; Custódio, L.; Rodrigues, M. J., Antioxidant activity, enzyme inhibition, photoprotection, cytotoxicity, and phytochemical profiling of sea lavender (Limonium algarvense Erben) seed extracts for dermo-cosmetic use. *Beni-Suef University Journal of Basic and Applied Sciences* **2024,** 13, (1), 112.
6. Barak, T. H.; Kurt Celep, İ.; Şentürk, T. B.; Bardakcı, H.; Celep, E., In Vitro Anti-Aging Potential Evaluation of Maclura pomifera (Rafin.) Schneider 80% Methanol Extract with Quantitative HPTLC Analysis. *Turk J Pharm Sci* **2022,** 19, (4), 400-407.
